# Early Metabolic Rewiring and Mitochondrial Remodeling in Response to Mitochondrial Impairment in a Rotenone Model of Parkinson’s Disease

**DOI:** 10.1007/s10571-026-01794-8

**Published:** 2026-08-12

**Authors:** Alissa Costantini, Marcella Bonanomi, Gessica Sala, Assunta Virtuoso, Anastasia Rella, Martina Motto, Claudio Gioia, Gabriele Taverna, Francesca Martorana, Federica Aprea, Matteo Formenti, Michele Papa, Carlo Ferrarese, Daniela Gaglio, Anna Maria Colangelo

**Affiliations:** 1 Laboratory of Neuroscience “R. Levi-Montalcini”, Department of Biotechnology and Biosciences, University of Milano-Bicocca, Piazza della Scienza 2, 20126 Milan, Italy; 2 Institute of Bioimaging and Complex Biological Systems, National Research Council (IBSBC-CNR), Via F.lli Cervi 93, 20090 Segrate, MI Italy; 3 Laboratory of Neurobiology, School of Medicine and Surgery, University of Milano-Bicocca, Monza, Italy; 4 Laboratory of Morphology of Neuronal Network, Department of Public Medicine, University of Campania “Luigi Vanvitelli”, Napoli, Italy; 5 Milan Center for Neuroscience NeuroMI, University of Milano-Bicocca, Milan, Italy

**Keywords:** Mitochondrial bioenergetics, Mitochondrial dynamics, Metabolomics, Metabolite set enriched analysis (MSEA), Oxidative stress, Parkinson’s disease

## Abstract

Mitochondrial dysfunction is a central pathogenic mechanism in Parkinson’s disease (PD), yet early mitochondrial events linking respiratory impairment to neuronal vulnerability remain incompletely defined. Here, we investigated the impact of acute and chronic exposure to subtoxic and toxic doses of the mitochondrial Complex I inhibitor rotenone on oxidative stress, mitochondrial bioenergetics/metabolism, and mitochondrial dynamics in primary cortical neurons. Using live-cell imaging, bioenergetics and metabolomic profiling, we show that nanomolar concentrations of rotenone rapidly induce accumulation of reactive oxygen species (ROS), lipid peroxidation, and loss of mitochondrial membrane potential preceding overt neuronal death. Notably, high-content morphometric analysis revealed that even subtoxic doses of rotenone trigger an early structural remodeling characterized by mitochondrial network fragmentation into small spherical puncta (0.5–3 μm) and localized organelle swelling at both neuritic and synaptic levels. Instead, at the molecular level, whole-cell biochemical degradation of mitochondrial remodeling proteins, suppression of regulated, physiological fission–fusion cycling and accumulation of the autophagy adaptor SQSTM1/p62, indicative of defective quality control, fully manifest only at higher, toxic rotenone concentrations. Furthermore, subtoxic doses elicit widespread metabolic alterations including persistent redox imbalance, impaired glutathione (GSH) biosynthesis, and downregulation of anabolic processes such as folate/one-carbon and amino sugar metabolism. Collectively, our findings newly reveal that localized mitochondrial morphometric remodeling and metabolic pathway rewiring represent early, interconnected adaptive responses to complex I impairment, contributing to the baseline thresholds of neuronal vulnerability in PD models.

**Graphical Abstract:**

**Figure:**
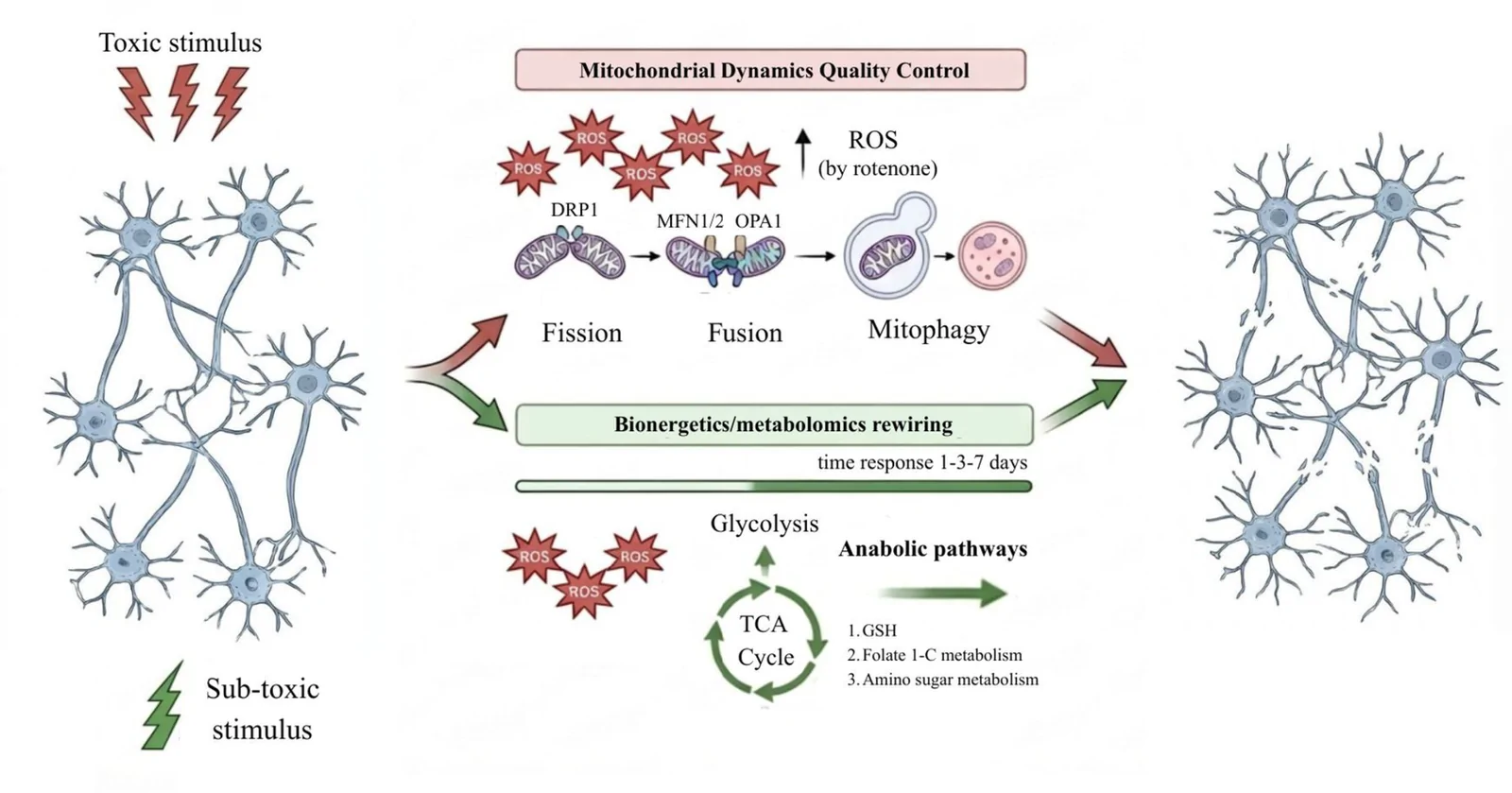

**Supplementary Information:**

The online version contains supplementary material available at https://doi.org/10.1007/s10571-026-01794-8.

## Introduction

Parkinson’s disease (PD) is a chronic and progressive neurodegenerative disorder characterized by the preferential loss of dopaminergic neurons within the nigrostriatal pathway and by the gradual involvement of additional brain regions, including the limbic cortex and frontal lobes (Navarro et al. 2009; Wang et al. 2023; Zhou et al. 2023).

Although the major pathological components underlying PD have been largely characterized, the interplay between the involved processes remains only partially resolved. Among these processes, mitochondrial dysfunction and impaired protein quality control –encompassing the ubiquitin-proteasome system (UPS) and distinct forms of autophagy– have been widely recognized as central mechanisms contributing to neuronal dysfunction and degeneration (Schapira et al. 1989; Martins-Branco et al. 2012; Shanbhag et al. 2012; Sala et al. 2016). These degradative pathways are intimately linked with mitochondrial homeostasis, ATP supply, and oxidative stress responses: their dysfunction promotes protein aggregation, including the accumulation of misfolded α-synuclein, a pathological hallmark of PD (Ashrafi and Schwarz 2013; Shanbhag et al. 2012; Xilouri et al. 2013).

Compelling evidence indicates that mitochondrial impairment plays a crucial role in PD pathogenesis. Reduced activity of mitochondrial Complex I has been reported in the *Substantia Nigra pars compacta* (SNpc) and frontal cortex of idiopathic PD patients (Schapira et al. 1989; Schapira 2007; Navarro et al. 2009). Mitochondria are essential for maintaining neuronal energy supply through oxidative phosphorylation, yet damaged mitochondria can become the major source of reactive oxygen species (ROS), particularly upon inhibition of Complex I, ultimately aggravating oxidative damage and enhancing protein aggregation (Sherer et al. 2003; Martins-Branco et al. 2012; Hattori and Sato 2024).

Mitochondria are highly dynamic organelles: their healthy state is maintained through dynamic processes involving biogenesis and fusion of mitochondria into elongated networks, trafficking along axons and dendrites, fission (i.e., segregation of damaged mitochondrial portions) and degradation by mitophagy (Archer 2013; Ashrafi and Schwarz 2013; Westermann 2010, 2012). These morpho-functional changes are controlled through the coordinated activity of the fusion proteins mitofusins1/2 (Mfn1/2) and optic atrophy-1 (Opa1) which regulate fusion of the outer and inner mitochondrial membrane, respectively; while fission is driven by dynamin-related protein-1 (Drp1), whose mitochondrial recruitment is controlled by reversible phosphorylation at Ser-616 and Ser-637 through PKA or CaMKI/calcineurin signaling (Cereghetti et al. 2008; Chang and Blackstone 2010; Westermann 2010). These remodeling events are fundamental for maintaining functional mitochondrial networks, adapting bioenergetic output to neuronal needs, and isolating damaged mitochondrial portions for mitophagic removal. Neurons are particularly reliant on efficient mitochondrial dynamics due to their polarized morphology and high synaptic energy demands. Consequently, dysregulation of mitochondrial distribution and turnover is increasingly considered an early pathogenic event in PD neurodegeneration (Archer 2013).

Recent studies have underscored how alterations in mitochondrial dynamics are intertwined with metabolic rewiring in many physiological conditions. During neuronal differentiation and brain development, mitochondrial remodeling sustains transitions in energy supply and metabolic pathways to comply with the requirements for morphological changes and the new specialized neuronal activity (Zheng et al. 2016; Goglia et al. 2024). Similar remodeling is likely triggered under pathological conditions that disrupt mitochondrial function, given the central metabolic role of mitochondria (Gielisch and Meierhofer 2015; Suciu et al. 2023). Transcriptomic analyses have revealed broad downregulation of gene sets involved in mitochondrial energy metabolism in PD models and in PD patient-derived tissues (Gielisch and Meierhofer 2015; Suciu et al. 2023; Ibanez et al. 2026), suggesting that metabolic collapse is an early feature of neurodegeneration.

Rotenone, a naturally occurring Complex I inhibitor, is widely used to model PD in vitro and in vivo. Rotenone exposure reproduces key pathological aspects of PD, including α-synuclein aggregation, mitochondrial dysfunction, increased ROS generation, and neuroinflammation (Sherer et al. 2003; Lal et al. 2024). Notably, its neurotoxicity is highly dose- and time-dependent. Primary neurons (such as dopaminergic, hippocampal or cortical neurons) exhibit vulnerability in the low nanomolar range (1–40 nM), whereas immortalized cell lines (such as SH-SH5Y neuroblastoma cells) often require micromolar concentrations (0.2–25 µM) to elicit mitochondrial dysfunction and cell death (Sala et al. 2016; Gordon et al. 2025).

We previously reported that rotenone induces dose-dependent neuronal loss and disrupts both macroautophagy and chaperone-mediated autophagy (CMA) in SH-SY5Y cells and primary cortical neurons (Sala et al. 2016). Here, we investigated the impact of acute (30 min–24 h) and chronic (3–7 days) exposure to low nanomolar (10–50 nM) or toxic doses (200–800 nM) of rotenone on oxidative stress, mitochondrial morphology, bioenergetics, and metabolic pathways in primary cortical neurons. We found that rotenone triggers early oxidative stress even at low concentrations, rapidly compromising mitochondrial function.

By combining automated high-content imaging with time-resolved metabolomics, we unveiled a spatial-temporal hierarchy in neuronal injury. Specifically, subtoxic doses of rotenone cause severe alterations in mitochondrial bioenergetics and a widespread rewiring of metabolic pathways, which occur concurrently with a significant structural remodeling of the network, characterized by network fragmentation into small 0.5–3 μm spherical puncta and localized organelle swelling at both neuritic and synaptic levels. Conversely, whole-cell dysregulation of mitochondrial remodeling proteins, and accumulation of the autophagic quality control adaptor SQSTM1/p62 fully manifest only in response to higher, toxic rotenone concentrations. As low nanomolar rotenone exposure mirrors more closely the latent, initial stages of complex I impairment in PD, the interconnected localized morphometric remodeling and the extensive metabolic adaptation observed here provide critical insights into the earliest bioenergetic thresholds governing neurotoxic vulnerability.

## Materials and Methods

### Drugs and Reagents

Neurobasal medium, B27 and bFGF were purchased from ThermoFisher Scientific Life Technologies. Penicillin/Streptomycin and L-glutamine were from Euroclone SpA, Italy. Poly-L-lysine was from SERVA Electrophoresis GmbH (Heidelberg, Germany). Oligomycin A (Olig), Rotenone (Rot), Antimycin A (AA) and Hoechst 33,342 were purchased from Sigma Aldrich/Merck, Italy. Dihydro-dichlorofluorescein diacetate (H_2_DCF-DA), MitoSOX™ Red, BODIPY™ 581/591 C11, MitoTracker Red CMXRos (MTR), MitoTracker Green FM (MTG) and Tetramethylrhodamine (TMRE) were purchased from ThermoFisher Scientific Life Technologies. Seahorse XF Cell Mito Stress Test Kit (Seahorse Bioscience) was purchased from Agilent Technologies, Inc., USA, while the NAD/NADH Quantitation Kit was from BioVision Inc., Milpitas CA, USA.

### Primary Cortical Neurons and Treatments

Cortical neurons were prepared as previously described with some modifications (Ahlemeyer and Baumgart-Vogt 2005; Cirillo et al. 2015). Animal experiments were carried out using protocols approved by the University of Milano-Bicocca Animal Care and Use Committee and by the Italian Ministry of Health (protocol number 14–2011), according to ARRIVE guidelines. Briefly, cortices were dissected from neonatal (P0-P1) C57BL/6J mice (Envigo, Italy) and carefully cleared of meninges under a dissecting microscope. Tissues were washed in dissociation medium and digested by trypsin (0.15%) with deoxyribonuclease (DNAse, 1 mg/ml, Sigma-Aldrich/Merck) at 37 °C for 20 min. After mechanical dissociation with a fire-polished Pasteur pipette, cells (1 × 10^6^/ml) were plated onto poly-L-lysine (0.1 mg/ml) coated dishes in serum-free Neurobasal medium (ThermoFisher Scientific Life Technologies) containing B27 supplement (ThermoFisher Scientific Life Technologies), bFGF 10 ng/ml (ThermoFisher Scientific Life Technologies), L-glutamine 0.5 mM (EuroClone) and antibiotics (EuroClone). Medium (half volume) was changed every other day and immediately before treatment. For all experimental settings, cultures were maintained at 37 °C in 5% CO_2_, imaged under a reversed microscope Olympus CK40-SLP equipped with Olympus camera (Olympus, Japan), and used at DIV8, the best time-point for pure neuronal metabolomics. To minimize proliferation of non-neuronal cells, AraC (1 µM) was added to the cultures after 2 days in vitro (DIV) and removed at DIV4. This well-established procedure, together with optimized culture conditions (serum-free Neurobasal/B27) consistently yields highly enriched neuronal cultures (typically > 95% purity), as previously validated by immunofluorescence using βIII-tubulin/GFAP staining (Ahlemeyer and Baumgart-Vogt 2005). Experiments were performed at DIV8, an optimized compromise to analyze differentiated neuronal bioenergetics while strictly minimizing the metabolic confounding background from residual proliferating glia.

### Cell Viability

The effect of Rotenone on cell viability was analyzed by the methylthiazolyldiphenyltetrazolium bromide (MTT) assay (Sigma-Merck) based on reduction of the yellow tetrazolium salts (MTT) to purple formazan by mitochondrial dehydrogenases in intact mitochondria. The assay was performed according to the manufacturer’s specifications. Briefly, cortical neurons (9 × 10^3^ cells/well) were plated in 96-well plates (EuroClone) pre-coated with poly-L-lysine (0.1 mg/ml). On DIV8, neurons were exposed to Rot (10–800 nM) for 6–24 h, followed by addition of tetrazolium salts (0.5 mg/ml) during the last 2 h of treatments at 37 °C in a humidified atmosphere 5% CO2/air. After incubation, MTT solubilization buffer (100 µl) was added to each well and plates were shaken for 1 h. Absorbance of samples was measured at 570 nm (630 nm reference wavelength) by using a microplate reader (Bio-Rad, Hercules, CA). MTT conversion levels were expressed as a percentage of control (CTR). Rot-induced cell death was also assessed by Trypan blue exclusion to confirm MTT results (data not shown).

#### Neuronal Soma Morphometric Analysis

Morphometric analysis of the neuronal somatic area was performed on phase-contrast images taken before and after treatments. For each experimental condition, the area of individual neuronal cell bodies (ranging from 80 to 120 neurons) was measured across multiple fields using ImageJ/Fiji software. Individual cell values were plotted to show the distribution of cell sizes within the neuronal population (Suppl. Figure 1), while statistical significance was assessed by performing a One-Way ANOVA on the mean values of three independent replicates per experimental group (*p* > 0.05).

### ROS and Lipid Peroxidation Measurement

For total ROS measurement, neurons (2 × 10^5^/well) were plated in 6-multiwell plates (EuroClone) coated with poly-L-lysine (0.1 mg/ml). On DIV8, neurons were exposed to Rot (10–800 nM) for 30 min or 3–6–24 h, in Neurobasal-B27 medium. Cells were loaded with 2’,7’-dichlorodihydrofluorescein diacetate (H_2_DCFDA) during the last 30 min of treatments, detached by trypsin, centrifuged at 600 *g*, resuspended in PBS and transferred into a black 96-multiwell plate (ThermoFisher Scientific Life Technologies). Fluorescence was measured using FLUOstar^®^Omega (BMG Labtech, Ortenberg, Germany) (excitation 485 nm, emission 520 nm), as previously reported (Goglia et al. 2024).

For mitochondrial ROS and lipid peroxidation, neurons (10^4^/well) were plated in glass bottom 96-multiwell plates (Perkin-Elmer) previously coated with poly-L-lysine (0.1 mg/ml) and used on DIV8. For ROS detection, neurons were loaded with MitoSOX red (absorption/emission: ∼396/610 nm) during the last 30 min of treatments, rinsed twice with PBS and placed in DMEM without phenol red for live imaging using Operetta^®^ CLS (Perkin-Elmer Inc., Waltham, MA, USA), as described below for mitochondrial imaging studies.

Lipid peroxidation was assessed using the lipid oxidation-sensitive probe BODIPY™ 581/591 C11. Upon oxidation, the probe shifts its fluorescence emission peak from red (∼590 nm) to green (∼510 nm). Neurons were loaded with BODIPY™ 581/591 C11 during the last 30 min of treatments, rinsed twice with PBS and placed in DMEM without phenol red for live imaging using Operetta^®^ CLS (Perkin-Elmer Inc., Waltham, MA, USA), as described below for mitochondrial imaging. The ratio of green-to-red fluorescence intensity was calculated to quantify relative lipid peroxidation levels.

### ATP Determination

Cortical neurons were seeded in complete NB-B27 medium, as described above (2 × 10^5^/well in 6-multiwell plates), and used on DIV8. After treatment with Rot (10–800 nM) for the indicated times, cells were lysed and ATP levels analyzed by using the Adenosine 5’-triphosphate (ATP) Bioluminescent Assay Kit (Sigma-Aldrich/Meck) according to manufacturer instructions. The light intensity was measured with a luminometer (Lumat LB9507, Berthold) for 5 sec and expressed as relative light units/µg of protein.

### NAD/NADH Assay

NAD^+^ and NADH levels were detected using NAD/NADH Quantitation Kit (BioVision Inc., Milpitas CA, USA) according to the manufacturer’s protocol. Briefly, cells (2 × 10^5^/well in 6-multiwell plates) were treated with Rot (10 nM) for 6–24−72 h on DIV8. After incubation, cells were washed in PBS and lysed in 400 µl of NADH/NAD extraction Buffer. After freeze/thaw cycles, samples were centrifuged at 14,000 rpm for 5 min and supernatants (50 µl) transferred into 96-well plates together with 100 µl of NAD Cycling mix (Buffer plus NAD Cycling Enzyme mix) to detect total NAD (NAD+NADH). Following 1–2 h incubation at RT and addition of Stop Solution (10 µl), colorimetric measurements were performed at OD 450 nm using a spectrophotometer (SmartSpec Plus, BioRad). To detect NADH only, samples were heated at 60 °C for 30 min to decompose NAD^+^. Total NAD and NADH levels were quantified on a standard curve (20, 40, 60, 80, and 100 pmol/well).

### Live Imaging of Mitochondrial Function and Morphology

Mitochondrial morphology (fragmented and networked) and membrane potential were assessed by Mitotracker green (MTG) and Tetramethylrhodamine (TMRE) staining, respectively. Briefly, dissected cortical neurons (10^4^/well) were plated in glass bottom 96-multiwell plates (Perkin-Elmer) previously coated with poly-L-lysine (0.1 mg/ml) and used on DIV8. For live imaging, cells were loaded with MTG (200 nM) or TMRE (100 nM) for 15 min after treatments, and counterstained with Hoechst (1 µg/ml) (Goglia et al. 2024). Neurons were then rinsed twice with PBS and placed in DMEM without phenol red for live imaging sessions. Images were acquired using Operetta^®^ CLS High-Content Analysis System (Perkin-Elmer Inc., Waltham, MA, USA) with a 63x water-immersion objective in confocal fluorescence mode. To capture the entire three-dimensional mitochondrial network, automated Z-stacks were acquired for each field. Images were processed using the dedicated Harmony^®^ software (Perkin-Elmer Inc.) to obtain quantitation of mitochondrial parameters. Automated segmentation blocks were configured to define cellular/mitochondrial regions of interest (ROIs) effectively discriminating true signal from background noise based on intensity thresholds, contrast, and precise morphological criteria. This automated workflow processed an average of 3,000 single cells per well across 49 randomly selected fields to guarantee unbiased, statistically robust data. For fluorescence quantification, the system extracted the Sum Fluorescence Intensity per field (corresponding to the area-corrected Integrated Density) from maximum intensity projection images, which was subsequently normalized to the number of nuclei tracked within the same imaging field.

For mitochondrial morphology by MTG, the software was instructed to recognize different classes of mitochondria (i.e. dispersed vs. networked). Measured parameters include number of mitochondria, total area (µm^2^) total volume (µm^3^), location in the cell (perinuclear vs. neurite region). All values were normalized by the total number of cells by Hoechst staining. Similar profiles were obtained in three independent experiments in duplicate.

For mitochondrial ROS and lipid peroxides, cells were loaded with MitoSox or Bodipy, respectively, and imaged by Operetta, as described above.

To assess the mitochondrial potential by Mitotracker^®^ Red CMXRos (MTR)/MTG staining, neurons (10^5^/well) were plated on glass bottom cell culture dishes (15 mm, NEST, China) previously coated with poly-L-lysine and exposed to Rot (10, 50, 200 nM). Neurons were loaded with MTR (50nM) and MTG (200 nM) for 15 min after treatments (Martorana et al. 2018), rinsed with PBS and placed in DMEM without phenol red. Live imaging was acquired using the Advanced Fluorescence THUNDER Imager Live Cell (LEICA Microsystem, Wetzlar, Germany) on about 200 cells for each condition in 10 random fields. Fluorescence intensity, mitochondrial areas and Integrated Fluorescence Intensities were quantified using ImageJ software. The MTR/MTG ratio allows to normalize mitochondrial membrane potential per unit of mitochondrial mass. Data, expressed as percent of CTR, are the mean ± SEM of three separate experiments, each performed in duplicate.

### Computer-Assisted Analysis of Synaptic Mitochondria

Mitochondrial fragmentation at the synaptic contacts was analyzed using the computer-assisted image analysis system MCID 7.1 (Imaging Res. Inc., Ontario, CA, USA). Valid mitochondrial targets within the selected synaptic regions of interest (ROIs) were objectively discriminated from the background using automated optical density, color, and spatial criteria within the Grain Count module of the software. To selectively quantify neurite parameters, the automated algorithm applied a digital soma-exclusion mask (conceptually designated as a non-somatic tracking block), effectively isolating neurite extensions from the main cell body. The grain count workflow automatically evaluated the Number of individual mitochondrial fragments contained within the selected ROIs, the Mean fragments Area (i.e. mean area of the grains) and the Mitochondrial Proportional Area (or Synaptic Mitochondrial Occupancy), i.e. the proportion of the synaptic ROIs that is occupied by grains. This automated, threshold-based approach ensures an unbiased evaluation of mitochondrial morphometry within the synaptic sites.

### Immunofluorescence Staining

Cortical neurons were seeded onto 12 mm poly-L-lysine-coated coverslips (2 × 10^4^ cells/well) and used on DIV8. After treatments, cells were washed with PBS, fixed with 4% paraformaldehyde, permeabilized with 0.25% Triton X100 in PBS and incubated with the blocking solution (10% normal goat serum, Cell Signaling Technologies), followed by overnight incubation with the primary antibodies rabbit Phospho(Ser616)-Drp1 (1:1000, Cell Signaling Technologies) and mouse Cox-IV (1:200, Cell Signaling Technologies). After washing with PBS, coverslips were incubated for 2 h at room temperature with goat anti-mouse Alexa 488™ and anti-rabbit Alexa 546™ conjugated antibodies (1:500; ThermoFisher Scientific Life Technologies), followed by nuclei counterstaining with Hoechst 33,342 (1 µg/ml) for 15 min. After washes, coverslips were mounted with Dako Fluorescent Mounting Medium (Dako Agilent Technologies, Santa Clara, CA, USA) and visualized under the Advanced Fluorescence THUNDER Imager Live Cell (LEICA Microsystem). Images of about 100 cells were taken in ten random fields for each condition. Images were taken with the same parameters and fluorescence intensity of cells was measured by NIH-ImageJ software and averaged to obtain the mean fluorescence intensity (MFI). Fluorescence intensity and co-localization of P(616)-Drp1(red)/CoxIV(green) by Pearson coefficient of correlation was measured on about 100 cells for each condition in 10 random fields. Data are the mean ± SEM of three independent experiments in duplicate.

### Bioenergetics by Seahorse Technology

Mitochondrial oxygen consumption rate (OCR) was determined by using a Seahorse XF96 Extracellular Flux Analyzer (Seahorse Bioscience, Copenhagen, Denmark). After dissection, cortical neurons (9 × 10^4^) were seeded on 96-XF plates in complete NB-B27 medium and used at DIV8 as described above. Cells were treated with Rot (10 or 50 nM) for 3, 6 or 24 h and then analyzed by using the Seahorse XF Cell Mito Stress Test Kit (Agilent Technologies, Inc., USA) according to manufacturer instructions. Three measurements of OCR and ECAR were taken for the baseline and after sequential injection of mitochondrial inhibitors. The ATP synthase inhibitor Oligomycin A (1 µM), the ATP synthesis uncoupler Carbonyl cyanide-4-trifluoromethoxyphenylhydrazone FCCP (0.8 µM), the complex I inhibitor Rotenone (50 nM) and complex III inhibitor Antimycin A (0.5 µM) were used to determine OCR parameters. OCR from each well were normalized by the protein content by using the Bradford assay (Bio-Rad, Hemel Hempstead, UK).

### Metabolic Profiling

Cortical neurons (5 × 10^5^/well) were plated on 6-well plates (Euroclone), previously coated with poly-L-lysine and exposed to Rot (3 or 10 nM) for 24 h, or 3 and 7 days in complete NB-B27 medium. At the end of treatments, metabolites extraction was performed as previously detailed in (Bonanomi et al. 2022; Goglia et al. 2024).

LC separation was performed using an Agilent 1290 Infinity UHPLC system equipped with an InfinityLab Poroshell 120 PFP column (2.1 × 100 mm, 2.7 μm; Agilent Technologies, Santa Clara, CA, USA). Mobile phase A consisted of water containing 0.1% formic acid (Honeywell, Wabash, IN, USA), while mobile phase B was acetonitrile with 0.1% formic acid. The injection volume was 10 µL. The gradient program was set as follows: 0–2 min, 100% A; 4 min, 99% A; 10 min, 98% A; 11–15 min, 70% A; and 16 min, return to 100% A, followed by a 2 min post-run. The flow rate was 0.2 mL/min and the column temperature was maintained at 35 °C.

Mass spectrometric detection was carried out on an Agilent 6550 iFunnel Q-TOF mass spectrometer equipped with a Dual JetStream ion source operating in negative ion mode (Agilent Technologies). Instrument settings were as follows: gas temperature 285 °C, gas flow 14 L/min, nebulizer pressure 45 psig, sheath gas temperature 330 °C, sheath gas flow 12 L/min, capillary voltage (VCap) 3700 V, fragmentor 175 V, skimmer 65 V, and octopole RF 750 V. Continuous internal mass calibration was achieved using a secondary nebulizer delivering reference masses at m/z 112.9855 and 1033.9881. Data were acquired over an m/z range of 60–1050. Along with the sample, 1 µl of reserpine 5 mg/L was injected as an internal standard.

### Metabolomics Data Analysis

Data analysis was performed with MassHunter ProFinder (Agilent Technologies) as in (Bonanomi et al. 2022; Goglia et al. 2024). Metabolomics datasets were further analyzed with MetaboAnalyst 6.0 (https://www.metaboanalyst.ca/). Features with more than 50% missing values were excluded prior to analysis. The remaining data were log-transformed (log10) and normalized using Pareto scaling. Statistical evaluation was performed through one-way ANOVA with a significance threshold set at *p* = 0.05. To correct for multiple comparisons, p values were adjusted using the Benjamini–Hochberg false discovery rate, followed by Fisher’s LSD post hoc tests. Significant metabolites were visualized using hierarchical clustering based on Ward’s method, enabling representation of normalized intensity values and grouping of both metabolites and experimental conditions according to similar metabolic profiles. The order of the columns in the heatmap was not determined arbitrarily. Instead, it is the mathematical result of an unsupervised hierarchical clustering algorithm embedded in the metabolomics analysis software. Enrichment analysis of significant metabolites was conducted using over-representation analysis against the SMPDB metabolite set library, comprising 99 metabolite sets. Over-represented pathways were identified using a hypergeometric test.

### Western Blot Analysis

Total cell extracts and western blotting were performed as previously described (Bianco et al. 2011; Martorana et al. 2018). Cell lysates were prepared by detergent lysis method. Briefly, neurons were washed in ice-cold PBS and scraped in lysis buffer [20 mM Tris pH 8.0; 137 mM NaCl; 1% Nonidet-P40; 10% glycerol; 1 mM dithiothreitol (DTT)] containing proteases inhibitors (Mini EDTA-free Protease Inhibitor Cocktail, Roche Applied Science, Sussex, UK) and phosphatases inhibitor cocktail (PhosSTOP, Roche Applied Science). After 20 min incubation on ice, cellular debris were pelleted by centrifugation at 14,000 g for 10 min at 4 °C. Protein concentration was determined by the Bradford protein assay (Bio-Rad, Hemel Hempstead, UK).

Total proteins (25 µg) were separated on 10% SDS-PAGE gels and transferred to nitrocellulose Protran™ (PerkinElmer, Waltham MA, USA). After blocking with 5% non-fat milk or BSA in Tris buffered saline with Tween (TBST) (10 mM Tris pH 7.5; 150 mM NaCl; 0.2% Tween-20), blots were probed overnight at 4 °C with the primary antibody in TBST, followed by incubation for 1 h at RT with HRP-conjugated donkey anti-rabbit or anti-mouse IgGs (1:5000; GE Healthcare Life Sciences, Buckinghamshire, UK). The following antibodies were used for immunoblots: Phospho(Ser616)-DRP1 [1:1000, rabbit monoclonal antibody (D9A1), Cell Signaling Technology (CST) #4494]; Phospho(Ser637)-DRP1 (1:1000, rabbit antibody, CST #4867), DRP1 [1:1000, rabbit monoclonal antibody (D6C7), CST #8570); Mitofusin-2 [1:100, rabbit monoclonal antibody (D2D10), CST #9482); OPA1 (1:1000, mouse antibody, BD Biosciences #612607), SQSTM1/p62 (1:1000, rabbit antibody, CST #5114). Blots were probed for β-actin (1:1000, mouse monoclonal antibody (8H10D10), CST #3700) to normalize for protein content. Detection was carried out by using the enhanced chemiluminescence system (SuperSignalTM West Pico PLUS Chemiluminescent Substrate, ThermoFisher Scientific Life Technologies). Quantification of bands was performed by densitometry using NIH-ImageJ software.

### Statistical Analysis

All data are presented as the mean ± SEM of three separate experiments with two independent samples (*N* = 6). Statistical analysis was performed by using GraphPad Prism 8.02 (GraphPad Software, La Jolla, CA, USA). All quantitative data were analyzed by one-way ANOVA and Dunnett’s multiple comparisons test for multiple treatments to assess significance differences between groups (* *p* ≤ 0.05, ** *p* ≤ 0.01, *** *p* ≤ 0.001, **** *p* ≤ 0.0001 *versus* the corresponding CTR), as indicated in the figure legends. For morphology analyses, individual images of CTR and treated neurons were assembled and the same adjustments were made for brightness, contrast and sharpness using Adobe Photoshop (Adobe Systems, San Jose, CA).

## Results

### Functional State of Mitochondria in Rotenone-Treated Cortical Neurons

Mitochondrial dysfunction is a central pathogenetic component of PD (Schapira et al. 1989) and rotenone is a well-established tool to model mitochondrial impairment through selective Complex I inhibition (Sala et al. 2016). To dissect mitochondrial events associated with neuronal dysfunction, we performed live imaging studies of mitochondria in primary cortical neurons exposed to subtoxic (10–50 nM) or toxic (200–800 nM) concentrations of Rot (Gordon et al. 2025).

**Fig. 1:**
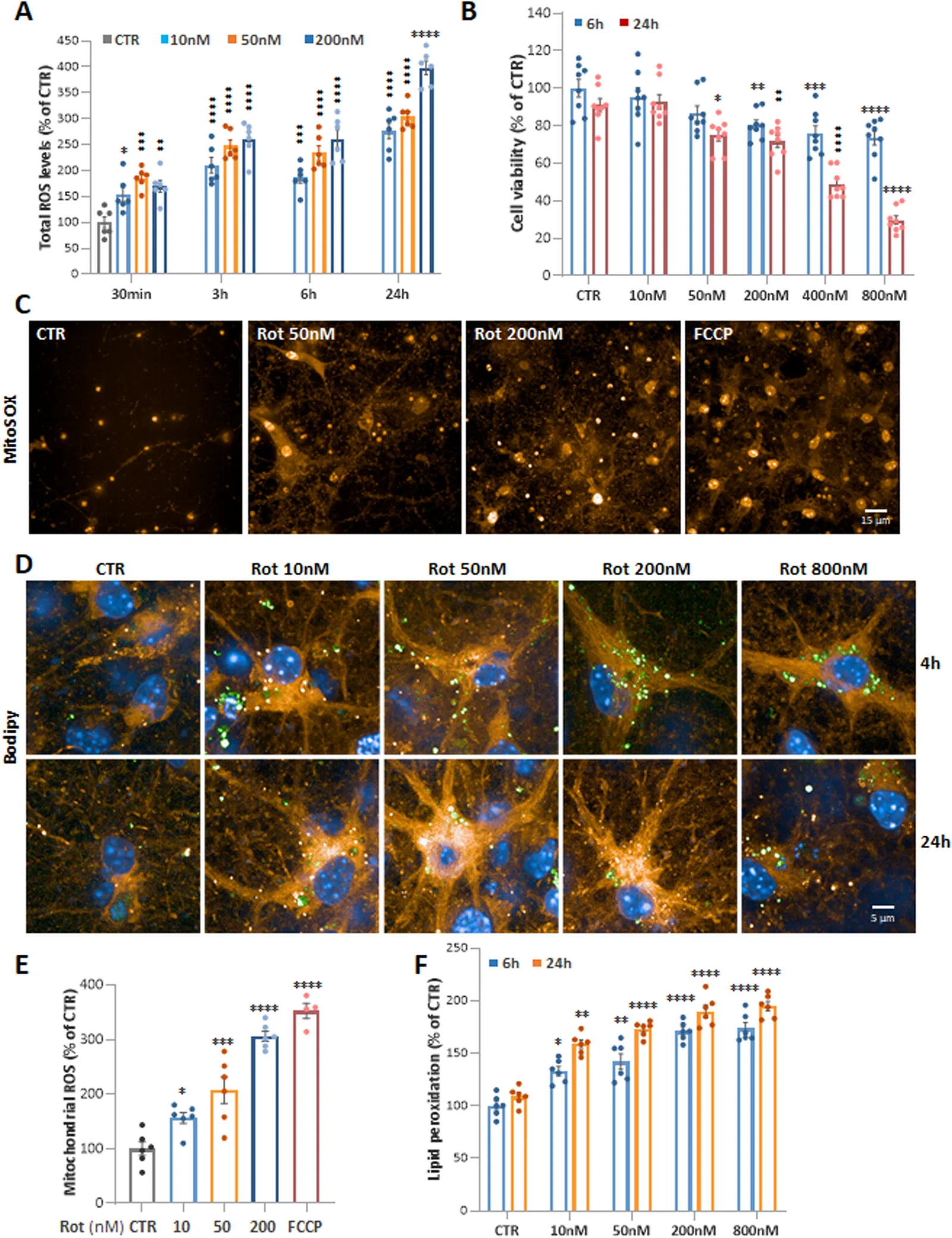
Oxidative stress in response to subtoxic and toxic Rotenone treatments: ROS and lipid peroxidation. **A** Fluorimetric determination of total ROS levels by H_2_DCFDA staining in cortical neurons treated with Rot (10, 50, or 200 nM) for 30 min or 3–6–24 h. **B** Cell viability by MTT assay in cortical neurons exposed to Rot (10, 50, 200, 400, 800 nM) for 6–24 h. **C** Live imaging of mitochondrial ROS by MitoSox staining of neurons treated with Rot (50 or 200 nM) for 4 h, as quantified in (E) (scale bar, 15 μm). FCCP (200 nM) was used as positive control. **D** Live imaging of lipid peroxidation by Bodipy staining of neurons treated with Rot (10, 50, 200 or 800nM) for 4–24 h, as quantified in (F) (scale bar, 5 μm). **E** Quantitation of mitochondrial ROS levels by MitoSox in cortical neurons treated with Rot (10, 50, or 200 nM) for 4 h (images in C). **F** Quantitation of lipid peroxides by Bodipy staining of cortical neurons treated with Rot (10, 50, 200, 800 nM) for 4–24 h (images in D). All measurements are normalized by the number of neurons in culture. Data, expressed as percent of CTR, are the mean ± SEM of three experiments with 2–6 independent samples (ROS: *N* = 2; MTT: *N* = 6; Live imaging: *N* = 2). ∗ ≤ 0.05, ∗∗ ≤ 0.01, ∗∗∗ ≤ 0.001, ∗∗∗∗ ≤ 0.0001 *versus* CTR (ANOVA and Dunnett’s multiple comparisons test)

We first quantified total and mitochondrial ROS generation. As previously reported, we found that Rot produces a dose- and time-dependent accumulation in total ROS levels as early as 30 min with all concentrations tested, including the subtoxic 10 nM (Fig. 1A), which does not reduce cell viability (Fig. 1B). In addition, all Rot concentrations (10, 50, 200 nM) also elicit a significant increase in mitochondrial ROS (Fig. 1C and E), as well as a strong rise in lipid peroxidation at 4 and 24 h, as revealed by live imaging of Bodipy staining (Fig. 1D and F), thus confirming that oxidative stress is a major early consequence of Complex I inhibition.

**Fig. 2:**
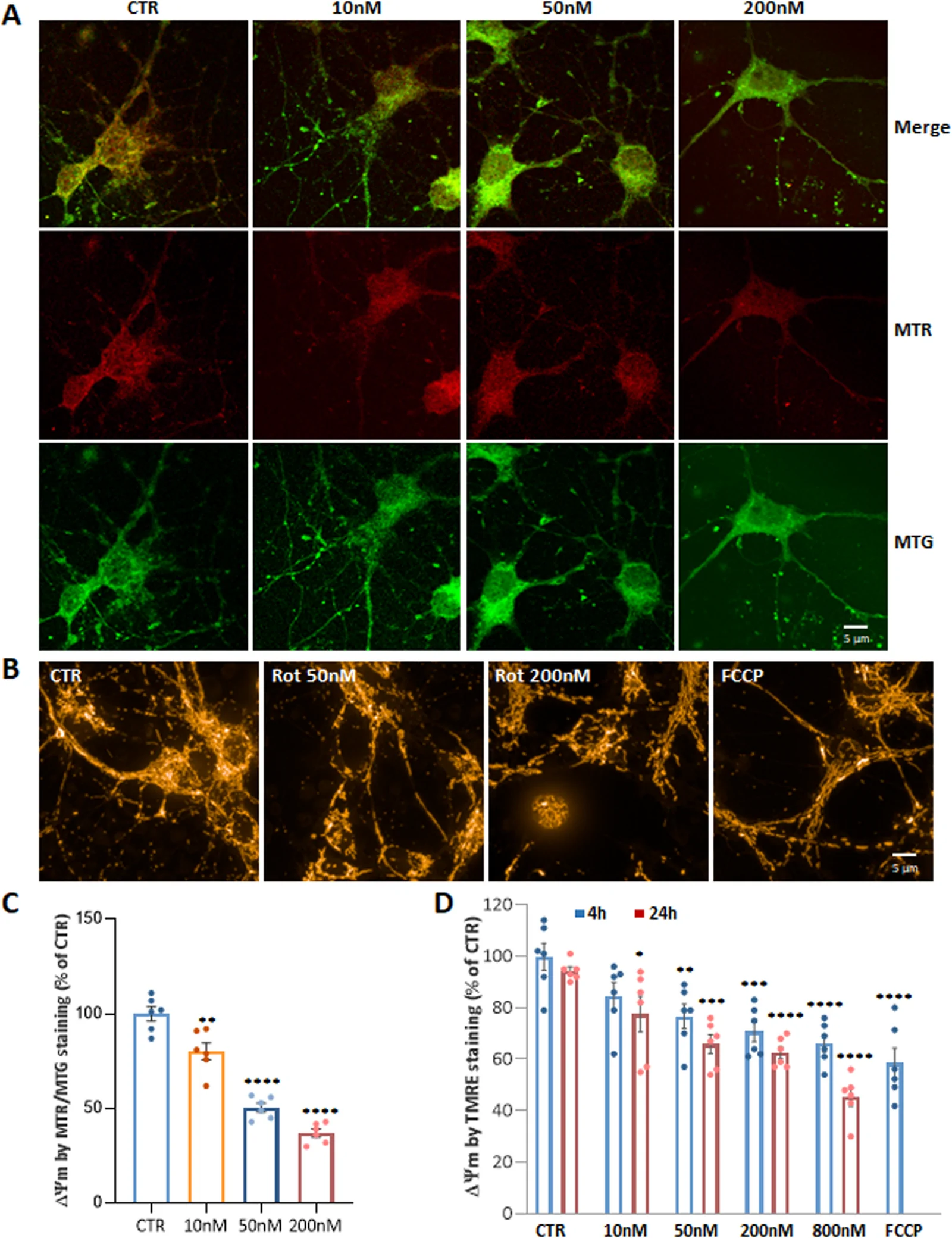
Mitochondrial membrane potential in Rotenone-treated neurons.** A** Representative images of cortical neurons exposed to Rot (10, 50, 200 nM) for 4 h, followed by MTR/MTG staining and imaging by confocal microscopy. Images were captured at 63x magnification (Plan Apo objective; 360 oil) using a motorized Nikon Eclipse 90i fluorescence microscope equipped with a CCD camera (scale bar, 5 μm). **B** Representative images of cortical neurons exposed to Rot (50 or 200 nM) for 4 h, followed by TMRE staining and live imaging using Operetta CLS (63x). Scale bar: 5 μm. FCCP (200 nM) was used as positive control. **C–D** Quantification of MTR (C) and TMRE (D) by ImageJ and Harmony software, respectively. Data, expressed as percent of CTR, are the mean ± SEM of the Integrated fluorescence Intensity/cell in three separate experiments with duplicate samples. ∗ ≤ 0.05, ∗∗ ≤ 0.01, ∗∗∗ ≤ 0.001, ∗∗∗∗ ≤ 0.0001 *versus* CTR (ANOVA and Dunnett’s multiple comparisons test)

To assess the functional state of mitochondria, we assessed the mitochondrial membrane potential using Mitotracker^®^ Red CMXRos (MTR) and Mitotracker Green (MTG) staining, to normalize mitochondrial membrane potential against total mitochondrial mass. As documented in Fig. 2, treatment of neurons with Rot for 4 h causes a significant reduction in the mitochondrial potential even at the subtoxic dose (10 nM Rot) primarily affecting mitochondria within neurites, while higher concentrations (50 and 200 nM) produce a widespread decline in the mitochondrial potential (Fig. 2A and C). Accurate morphological and quantitative analysis of mitochondrial potential was then achieved by live imaging of Tetramethylrhodamine (TMRE) stained mitochondria in neurons exposed to Rot (10, 50, 200 or 800 nM) for 4–24 h: computer-assisted quantitation revealed a dose- and time-dependent loss in mitochondrial potential (Fig. 2B and D), indicating broad impairment of mitochondrial function.

Importantly, preliminary morphometric control analysis confirmed that the average neuronal somatic area of surviving neurons remained statistically unchanged across all Rot concentrations and timepoints compared to CTR (Suppl. Figure 1), thereby ruling out cell shrinkage or swelling artifacts in our fluorescence and bioenergetic quantifications.

### Mitochondrial Bioenergetics and ATP Production in Rotenone-Treated Neurons

**Fig. 3:**
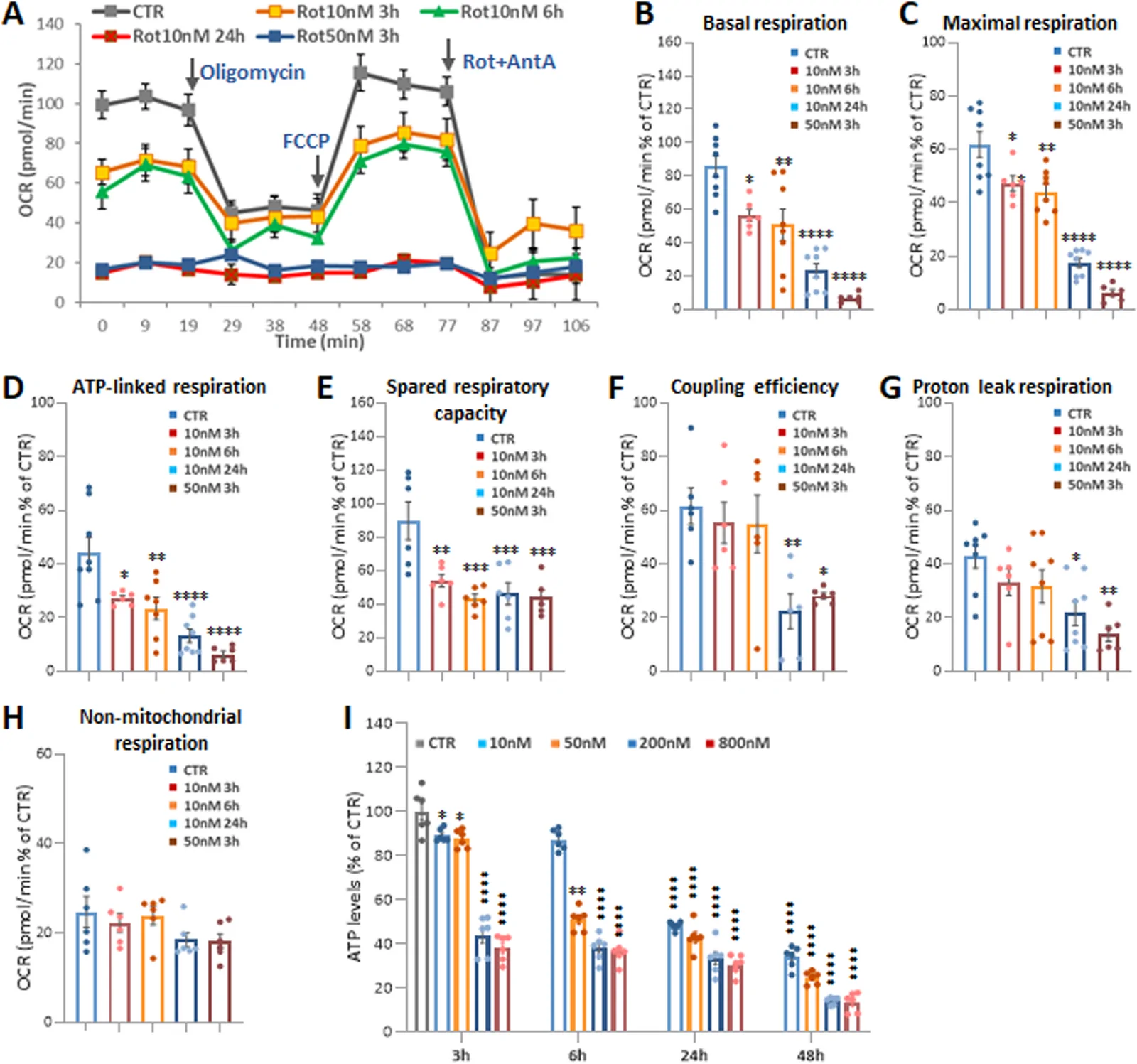
Energy-stress response in Rotenone-treated neurons.** A** Representative OCR profiles of cortical neurons exposed to subtoxic doses of Rot (10 and 50 nM) for 3, 6–24 h. Oligomycin A (0.5 µM), FCCP (0.8 µM), Rot (50 nM) plus Antimycin A (AntA, 0.5 µM) were injected at the indicated times to measure the OCR linked to ATP turnover, maximal respiration and proton leak, respectively. Data are the mean ± SEM of four samples, each normalized by the corresponding protein content. Similar profiles were obtained in six independent experiments. **B–H** Bioenergetics parameters: basal, maximal and ATP-linked respiration, spared respiratory capacity, coupling efficiency, Proton Leak and non-mitochondrial respiration in Rot-treated neurons. Data are the mean ± SEM of six separate experiments, each with 4–5 independent samples. **I** Time-course of ATP levels in cortical neurons treated with Rot (10, 50, 200, 800 nM) for the indicated times. Data, expressed as percent of CTR, are the mean ± SEM of three experiments with two independent samples. ∗ ≤ 0.05, ∗∗ ≤ 0.01, ∗∗∗ ≤ 0.001, ∗∗∗∗ ≤ 0.0001 *versus* CTR (ANOVA and Dunnett’s multiple comparisons test)

To evaluate the impact of Rotenone on mitochondrial bioenergetics, we measured the oxygen consumption rate (OCR) in neurons treated with low dose Rot (10 and 50 nM). Although viability remained unaffected (Fig. 1B), we found that 10nM Rot significantly impairs neuronal OCR at both 3 and 6 h (Fig. 3A) and leads to a dramatic reduction in all the bioenergetic parameters: basal respiration, maximal respiration, ATP-linked respiration, and spared respiratory capacity all decline sharply (Fig. 3B–E). After 24 h, OCR profiles revealed a near-complete collapse of mitochondrial respiration (Fig. 3A) and further decrease in all bioenergetic parameters (Fig. 3B–E), together with Proton Leak respiration and coupling efficiency, without significant decline in non-mitochondrial respiration (Fig. 3F–H). An early collapse of mitochondrial respiration was observed with 50 nM Rot (Fig. 3A–H) and other toxic concentrations (200–400 nM; data not shown).

Remarkably, the bioenergetic deficit was substantiated by a significant reduction in ATP production: specifically, ATP measurements showed that 10nM Rot produces a 20% reduction in ATP levels at 3–6 h; whereas prolonged (24–48 h) exposure to 10nM Rot or higher concentrations (50, 200, 800nM) produce a dramatic loss (50–60% decrease) – or nearly complete depletion – in ATP levels (Fig. 3I), suggesting that mitochondrial bioenergetics is heavily compromised.

###  Metabolomic Profiles Reveal a Transient Rewiring of Energy and Lipid Metabolism

**Fig. 4:**
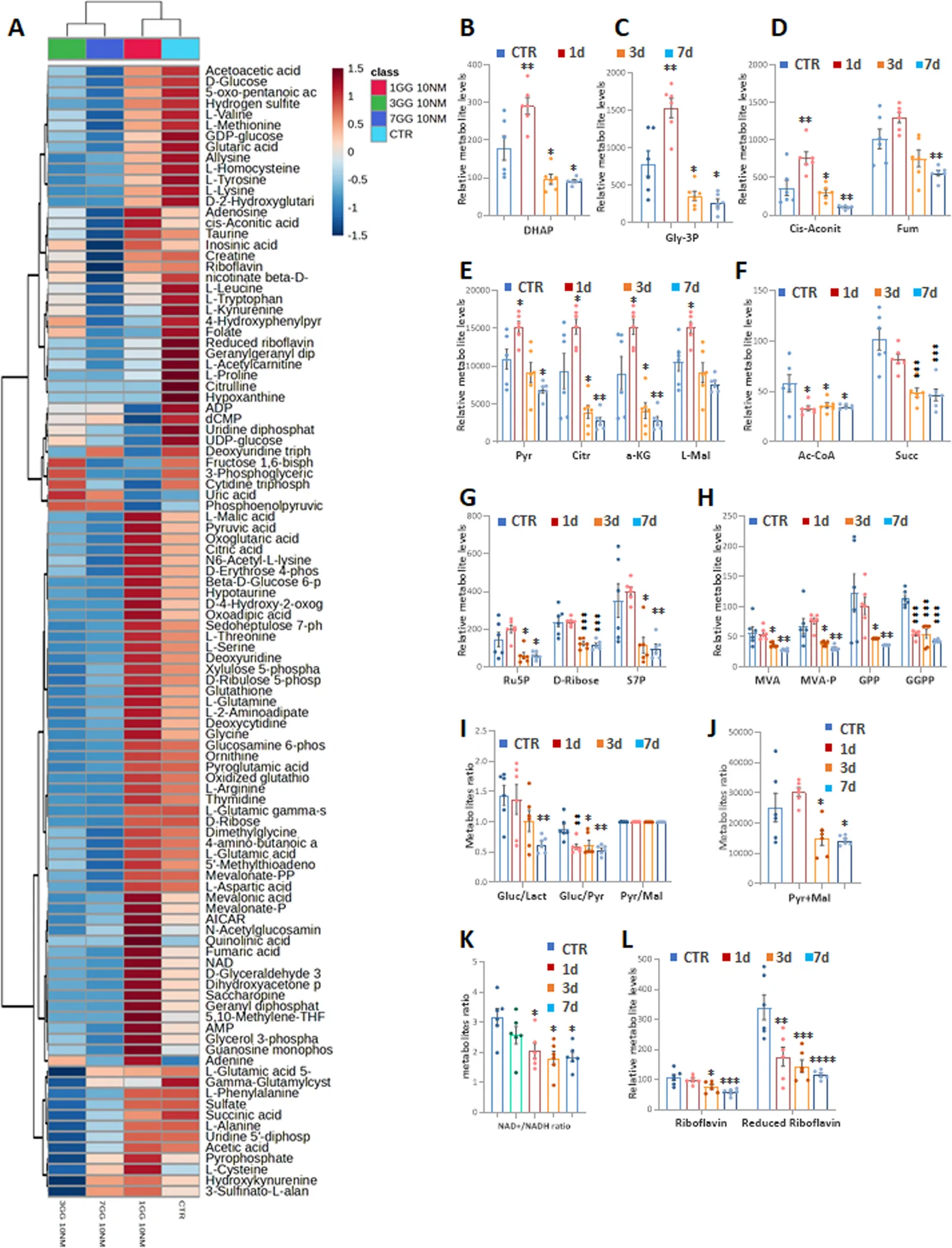
Untargeted metabolic profiling of Rotenone-treated neurons.** A** Hierarchical clustering heatmap showing significantly different intracellular metabolites by LC-MS in cortical neurons treated with Rot 10nM for 24 h or 3–7 days. Please, note that the order of columns is mathematically generated by unsupervised clustering (Color scale: from blue to red). **B–H** Relative metabolite levels of: DHAP (B), Gly-3P (C); cis-Aconitate and Fum (D); Pyr, Citr, αKG and L-Mal (E); Ac-CoA and Succ (F); Ru5P, D-Ribose and S7P (G); MVA, MVA-P; GPP and GGPP (H). **I** Metabolites ratios relative to glycolysis (Gluc/Lact, Gluc/Pyr) and TCA cycle (Pyr/Mal). **J** Sum of Pyr plus Mal levels. **K** NAD^+^/NADH ratio. **L** Relative abundance of Riboflavin and reduced riboflavin. Data are the mean ± SEM of three experiments with two independent samples. ∗ ≤ 0.05, ∗∗ ≤ 0.01, ∗∗∗ ≤ 0.001, ∗∗∗∗ ≤ 0.0001 versus their respective CTR (ANOVA and Dunnett’s multiple comparisons test). DHAP: Dihydroxyacetone phosphate; Gly-3P: Glycerol 3-phosphate; Fum: Fumarate; Pyr: Pyruvate; Citr: Citrate; αKG: α-Ketoglutarate; L-Mal: L-Malate; Ac-CoA: Acetyl-CoA; Succ: Succinate; Ru5P: D-Ribulose 5-phosphate; S7P: Sedoheptulose 7-phosphate; MVA: Mevalonate; MVA-P: Mevalonate-P; GPP: Geranyl diphosphate; GGPP: Geranylgeranyl diphosphate

**Fig. 5:**
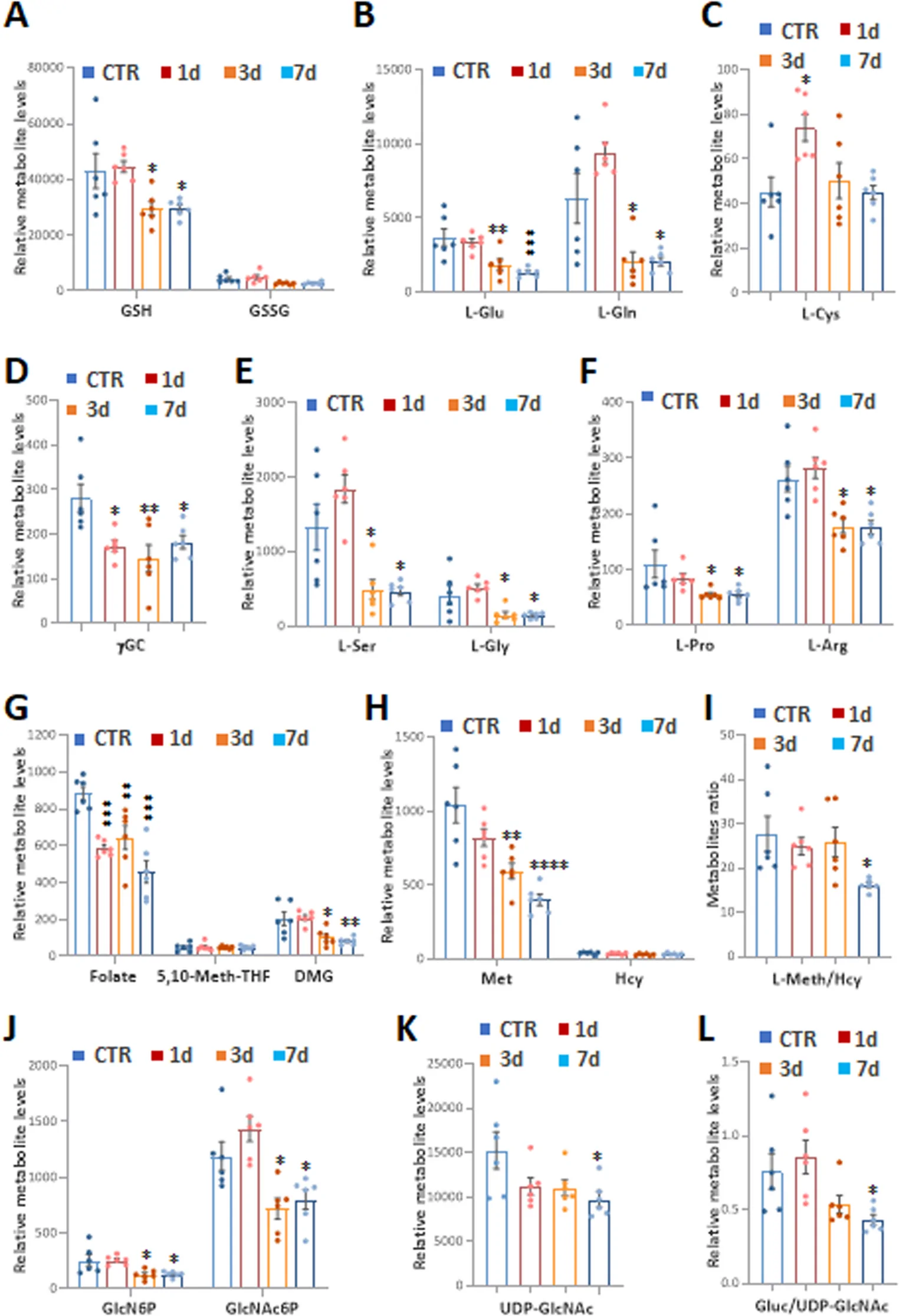
Impact of Rotenone on GSH, aminoacids and folate 1 C-metabolism. **A–E** Relative abundance of GSH and GSSG (A) and their precursors/intermediates: L-Glu and L-Gln (B), L- Cys (C), γGC (D), L-Ser and L-Gly (E). **F** Levels of L-Pro and L-Arg. **G–I** Levels of folate 1 C-metabolism intermediates: folate, 5,10-CH_2_-THF and DMG (G), L-Met and Hcy (H), and L-Met/Hcy ratio (I). **J–L** Levels of the amino sugars GlcN6P and GlcNAc6P (J), UDP-GlcNAc (K) and Gluc/UDP-GlcNAc ratio (L). Data are the mean ± SEM of three experiments with two independent samples. ∗*p* ≤ 0.05, ∗∗*p* ≤ 0.01, ∗∗∗*p* ≤ 0.001, ∗∗∗∗*p* ≤ 0.0001 *versus* their respective CTR (ANOVA and Dunnett’s multiple comparisons test). GSH: reduced glutathione; GSSG: oxidized glutathione; L-Glu: L-Glutamate; Gln: Glutamine; L-Cys: L-Cysteine; γGC: γ-glutamyl cysteine; L-Ser: L-Serine; L-Gly: L-Glycine; L-Pro: Proline; L-Arg: Arginine; 5,10-CH_2_-THF: 5,10-methylene-THF; DMG: Dimethylglycine; L-Met: L-methionine; Hcy: homocysteine; GlcN6P: Glucosamine-6-phosphate; GlcNAc6P: N-acetylglucosamine 6-phosphate; UDP-GlcNAc: Uridine diphosphate N-acetylglucosamine

To further explore the metabolic consequences of mitochondrial dysfunction, we performed metabolomic analysis after acute (24 h) and chronic (3–7 days) treatment with 10nM Rot. The heatmap revealed 136 metabolites with statistically significant changes in Rot-treated neurons (Fig. 4A). The metabolic signature at 24 h shows 23 downregulated and 19 upregulated metabolites involved in the second branch of glycolysis and in TCA cycle, together with components of the malate–aspartate shuttle, pyruvate metabolism, pentose phosphate pathway (PPP), glutamine and serine/glycine metabolism, and the mevalonate pathway (Figs. 4 and 5).

Notably, the unbiased clustering structure revealing the true metabolic relationships was not determined arbitrarily but is the mathematical result of an unsupervised hierarchical clustering algorithm embedded in the metabolomics analysis software.

Specifically, the heatmap shows a significant increase in Dihydroxyacetone phosphate (DHAP) and Glycerol 3-phosphate (Gly-3P) (Fig. 4B-C), and TCA cycle intermediates [cis-Aconitate Citrate (Citr), α-Ketoglutarate (αKG), Fumarate (Fum) and L-Malate (L-Mal)] (Fig. 4D-E), together with a modest rise (or minimal change) in PPP intermediates [D-Ribulose 5-phosphate (Ru5P), D-ribose and Sedoheptulose 7-phosphate (S7P)] (Fig. 4G) and lipid-associated components [Mevalonate (MVA), Mevalonate-P (MVA-P), Geranyl diphosphate (GPP) and Geranylgeranyl pyrophosphate (GGPP)] (Fig. 4H).

This coordinated metabolic modulation at 24 h is coherent with an early survival response to Rot exposure. The rise in metabolites of redox-related metabolic routes suggests an attempt to control the oxidative damage while maintaining energy production and mitochondrial membranes integrity during the acute phase. However, all these metabolites are significantly reduced by day-3/7 (63 and 74 downregulated metabolites, respectively) indicating that the early metabolic pattern observed at 24 h is not maintained under prolonged Rot treatment (Fig. 4A–H).

Likewise, neurons facing complex I inhibition suffer a persistent decrease in the Gluc/Pyr ratio (Fig. 4I) and the corresponding decrease in Acetyl-CoA (Ac-CoA) and Succinate (Succ) levels (Fig. 4F), together with the significant reduction in the sum Pyr + Mal (Fig. 4J), while the Gluc/Lact ratio is stable at 24 h and sharply drops by day-3/7 (Fig. 4I). These changes are indicative of altered TCA cycle control, while a stable Pyr/Mal ratio suggests no effect on the forward route of the TCA cycle (Fig. 4I). Remarkably, the rise in DHAP and Gly-3P at 24 h (Fig. 4B–C) suggests a higher utilization of the Gly-3P shuttle to regenerate NAD^+^ from NADH and foster glycolysis, while FADH_2_ (generated by the mitochondrial Gly-3P dehydrogenase) is oxidated by succinate dehydrogenase (Complex II), thus bypassing Complex I.

All conditions are characterized by alteration in the redox state, as revealed by the significant reduction in the NAD^+^/NADH ratio (Fig. 4K); Riboflavin and reduced Riboflavin [reduced Flavin Adenine Dinucleotide (FADH_2_) and Flavin Mononucleotide (FMNH_2_)] are also strongly affected (Fig. 4L).

**Fig. 6:**
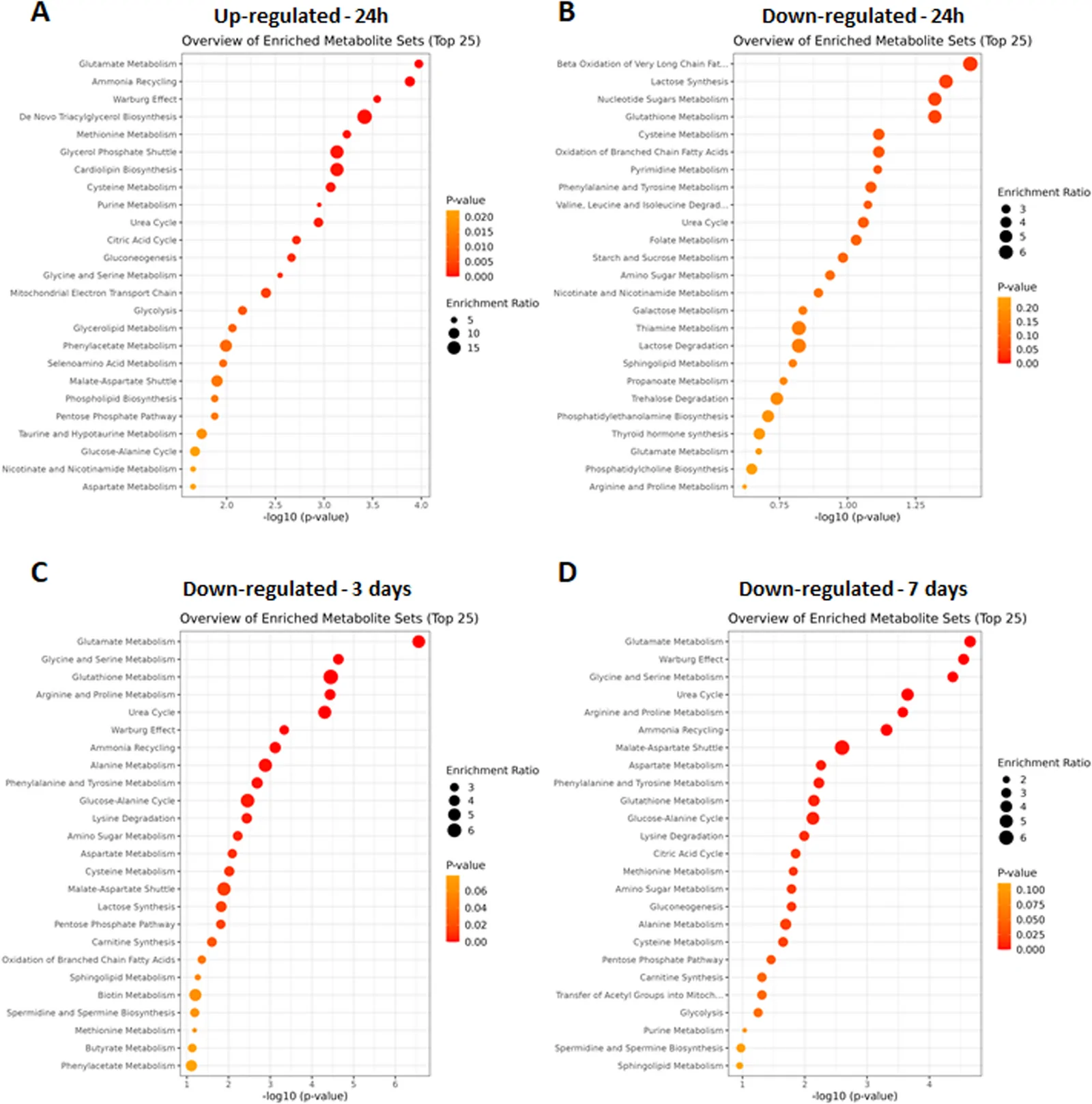
Metabolite Set Enrichment Analysis (MSEA) of Rotenone-treated neurons. **A–D** Enriched metabolites pathways in cortical neurons treated with Rot (10 nM) for 24 h (A-B, up- and downregulated, respectively) or 3–7 days (C-D, downregulated), showing the enriched metabolic pathways and the corresponding enrichment ratios (ER) and P-value (Color scale: light to dark red)

To further understand the impact of metabolites differentially changed upon complex I inhibition, we performed metabolite set enrichment analysis (MSEA) (Fig. 6) showing indeed a strong upregulation of the Gly-3P shuttle (Enrichment ratio, ER of 15 metabolites, P-value ≤ 0.001) and a modest increase in TCA cycle, glycolysis and PPP at 24 h (ER = 6–9, P-values ≤ 0.05) (Fig. 6A). These pathways are all downregulated by day-3/7, together with the malate-aspartate shuttle (Fig. 6C–D; ER = 6, *P* ≤ 0.01). Remarkably, no upregulated pathways are present on day-3/7.

Highly enriched pathways at 24 h include *De novo* Triacylglycerol biosynthesis and Cardiolipin biosynthesis (ER = 15, *P* ≤ 0.001), the major phospholipid of the inner mitochondrial membrane (Fig. 6A). Glycolipid and Phospholipid biosynthesis are upregulated as well (Fig. 6A; ER = 6–7, *P* ≤ 0.01), while downregulated metabolite sets at 24 h include oxidation of long chain and branched fatty acids (ER = 5–6, *P* ≤ 0.005), along with a persistent reduction in sphingolipid metabolism (Fig. 6B–D).

Altogether, these data suggest that neuronal exposure to subtoxic 10nM Rot for 24 h generates an initial metabolic rewiring aiming to counteract the complex I-mediated decline in aerobic respiration through the Gly-3P shuttle, while Gly-3P (backbone of glycolipids and phospholipids), together with a minimally affected Mevalonate pathway and reduced fatty acid oxidation, allow the maintenance of membranes integrity. However, by day-3/7 this initial state progresses toward a total disruption of metabolic neuronal homeostasis to an extend comparable to the highly toxic Rot doses observed in other cellular models (Suciu et al. 2023; Wang et al. 2023). A similar trend was found in neurons exposed to 3nM Rot for 1–3−7 days (Suppl. Figure 2).

### Impact of Rotenone on Glutathione and Aminoacids Metabolism

The heatmap also reveals significant changes in glutathione (GSH) metabolism (Fig. 4A), as shown by the reduction in GSH levels on day-3/7, without a corresponding increase in GSSG levels (Fig. 5A). Remarkably, metabolomics data show major changes in the three GSH aminoacids: L-Glutamate/L-Glutamine (Gln/Glu), L-Cysteine (Cys) and L-Serine/L-Glycine (Ser/Gly) levels are all decreased (Fig. 5B–C and E–F), together with a significant reduction in γ-glutamyl cysteine (γGC) at all time-points (Fig. 5D) reflecting an imbalance in the initial step of *de novo* GSH synthesis.

These changes were observed by MSEA as well: GSH metabolism is among the most downregulated pathways (ER = 6, *P* ≤ 0.01) at both 24 h and day-3/7, together with reduced Glut, Cys and Ser/Gly metabolism (Fig. 6B–D), which validate the relevance of reduced *de novo* GSH synthesis from its precursors, as previously reported by targeted metabolomics studies (Huang et al. 2023). Moreover, metabolomics data and the corresponding MSEA also display a persistent decrease in L-Aspartate and L-Arginine/L-Proline (Arg/Pro) metabolism (Figs. 5F and 6C–D; ER = 4–6 metabolites, *P* ≤ 0.01) which, together with lower Glu/Gln cycle, participate in the reduction of ammonia recycling and urea cycle (Fig. 5B–D). Altogether, these changes align with decreased antioxidant capacity, combined with decreased aminoacid metabolism and ammonia recycling.

### Impairment in Energy Production Impacts the Anabolic Metabolism

Impairment in energy homeostasis largely impacts anabolic reactions as well. In contrast with the early increase in Glycolipid/Phospholipid metabolism and stable MVA intermediates levels at 24 h (Figs. 4H and 6A) and their potential rescue of membrane stability, our data revealed a decrease in folate 1 C-metabolism. Rot-treated neurons show a significant reduction in folate levels and other intermediates [Dimethylglycine (DMG) and 5,10-methylene-THF] at both 24 h and day-3/7 (Fig. 5G), together with the reduction in L-methionine (L-Met) levels and relative accumulation in homocysteine (Hcy), as revealed by a lower L-Met/Hcy ratio on day-7 (Fig. 5H–I). These changes are evident by MSEA as well, as indicated by the significant reduction in folate metabolism at 24 h (Fig. 6B; ER = 4, *P* ≤ 0.01), and L-Met metabolism on day-3/7 (Fig. 6C–D; ER = 3, P-value ≤ 0.05).

Likewise, MSEA shows the downregulation of Pyrimidine and Nucleotide Sugar metabolism at 24 h (Fig. 6B; ER = 3–6, *P* ≤ 0.001), together with a reduction in hexosamine/Amino Sugar metabolism at both 24 h and day-3/7 (Fig. 6B–D; ER = 5, *P* ≤ 0.05). Specifically, Rot-treated neurons show a significant rise in N-acetylglucosamine 6-phosphate (GlcNAc-6P) at 24 h, followed by its reduction on day-3/7, along with reduced Glucosamine-6-phosphate (GlcN6P) and Uridine diphosphate N-acetylglucosamine (UDP-GlcNAc) levels, and lower Gluc/UDP-GlcNAc ratio (Fig. 5H–J). UDP-GlcNAc is used in O-GlcNAcylation, suggesting that 10nM Rot impairs protein glycosylation, trafficking and other processes crucial to neuronal function, such as modification of synaptic proteins (Seo et al. 2025). Altogether, these data suggest that impairment of energy-related metabolic pathways largely impacts all the anabolic routes under chronic (3/7-days) condition.

### Modification of Mitochondrial Dynamics in Rotenone-Treated Neurons

**Fig. 7:**
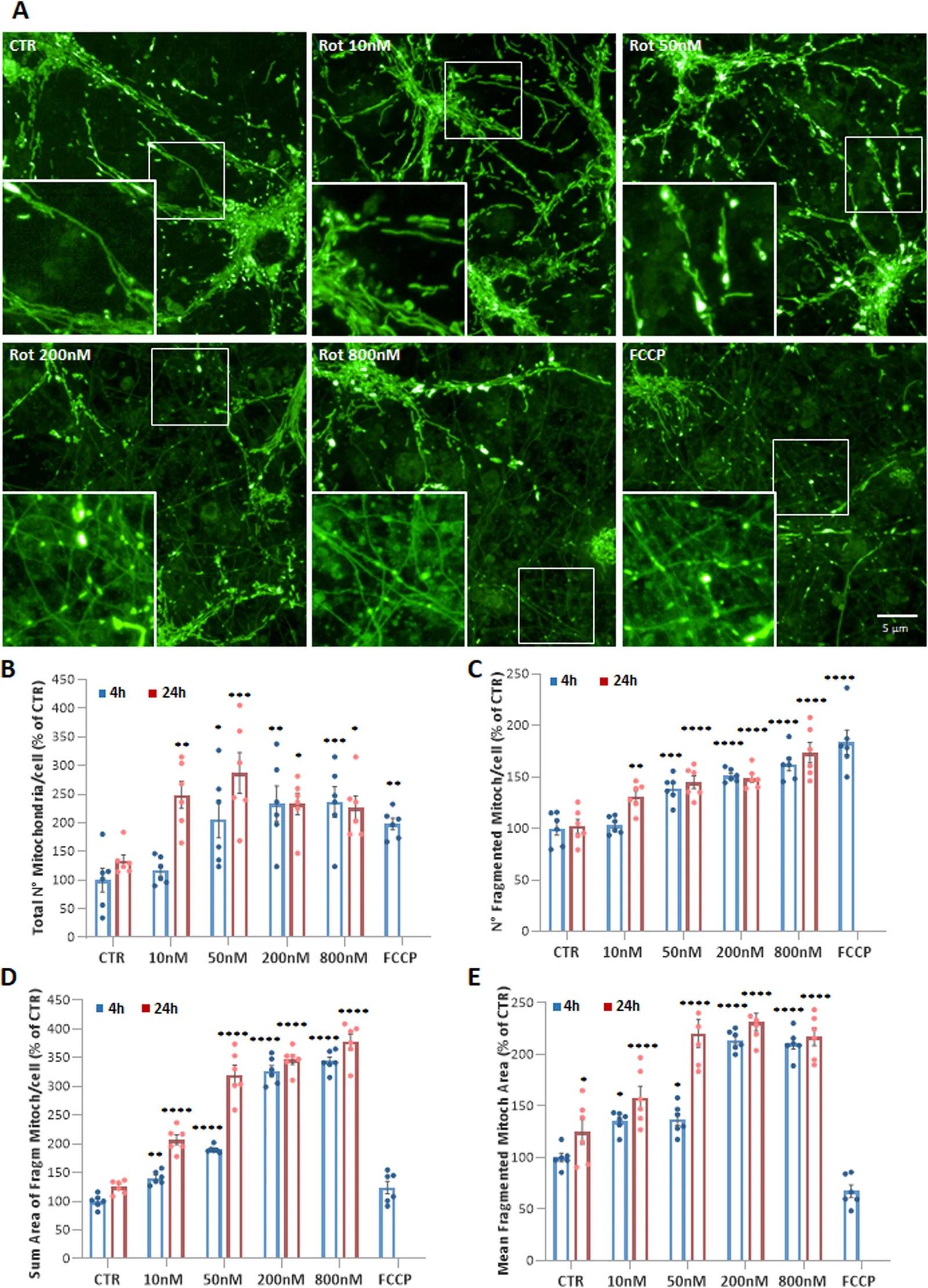
Rotenone triggers time- and dose-dependent mitochondrial fragmentation and swelling.** A** Representative images of primary cortical neurons stained with MitoTracker Green (MTG) following exposure to vehicle (CTR), FCCP (200 nM), or different concentrations of Rotenone (10–50-200–800 nM) for 4 h. MTG-stained mitochondria were observed in live-imaging using Operetta CLS^®^ (63x). High-magnification (2x) cropped insets display the progressive transition from networked (CTR) to fragmented “punctate and swollen” morphology (50 nM), culminating in mitochondrial loss along “ghost” neurites (200–800 nM and FCCP); here, insets brightness was linearly adjusted to better resolve ‘ghost neurites’). Scale bar, 5 μm. **B–D** Automated quantitation of B) total number of mitochondria per cell, C) number of spherical “punctate” mitochondria (0.5–3 μm), and D) Sum Area of these fragmented population per cell. **E** Morphometric evaluation of the Mean Fragmented Mitochondrial Area (calculated as Area/Number ratio) within the 0.5–3 μm subpopulation validating single mitochondria swelling. Data, expressed as percent of CTR are the mean ± SEM from three experiments with duplicate samples. ∗ ≤ 0.05, ∗∗ ≤ 0.01, ∗∗∗ ≤ 0.001, ∗∗∗∗ ≤ 0.0001 *versus* their respective CTR (ANOVA and Dunnett’s multiple comparisons test)

Changes in mitochondrial function and metabolism are intricately linked, with altered mitochondrial dynamics playing a crucial role in this context (Westermann 2010, 2012; Goglia et al. 2024). To characterize this connection in response to subtoxic or toxic Rot, we assessed mitochondrial morphology in cortical neurons exposed to Rot (10, 50, 200, 800 nM) through live-cell imaging studies using MTG staining (Fig. 7A). Quantitation revealed that Rot-treated neurons exhibit a significantly higher number of total mitochondria per cell (Fig. 7B). Specifically, while the subtoxic 10 nM concentration did not produce any alteration at 4 h, it led to a significant increase in mitochondrial number at 24 h. Conversely, higher concentrations (50, 200, and 800 nM) triggered a robust increase in total mitochondrial counts at both timepoints (Fig. 7B).

To precisely characterize mitochondria remodeling without relying on potentially biased automated tracking of long or intermediate mitochondrial networks, we focused our morphometric analysis on the small, fragmented mitochondria subpopulation (0.5–3 µm diameter). Strikingly, we observed a robust, dose- and time-dependent increase (1.5–2-fold) in the number of these spherical ‘punctate’ mitochondria in Rot-treated neurons (Fig. 7C). Furthermore, evaluating the total mass and the individual sizes within this fragmented population revealed a significant (2–3-fold) increase in the Sum Area (Fig. 7D) and, crucially, a 1.5–2-fold increase in the Mean Fragmented Mitochondrial Area (Fig. 7E, calculated as the Area/Number ratio). As expected, the positive control FCCP (200 nM for 4 h) triggered immediate network fragmentation into small punctate structures (Fig. 7A–C), without inducing the true organelle swelling phenotype observed under rotenone treatment.

Because our independent control data show that the overall neuronal somatic area remains stable under these conditions (Suppl. Figure 1), this concurrent increase in fragment number, total area, and single-fragment area confirms that the network fractures into small spherical puncta which subsequently undergo structural swelling and dispersion, a hallmark of severe bioenergetic stress, as previously shown in cortical neurons under oxygen-glucose deprivation (Anzell et al. 2021). Importantly, this structural fracturing is rapid and fully manifest within 4 h of exposure to toxic Rot concentrations (Fig. 7A), whereas the subtoxic 10 nM triggers a more progressive remodeling, producing significantly swollen and fragmented mitochondria after 24 h (Fig. 7C–E).

**Fig. 8:**
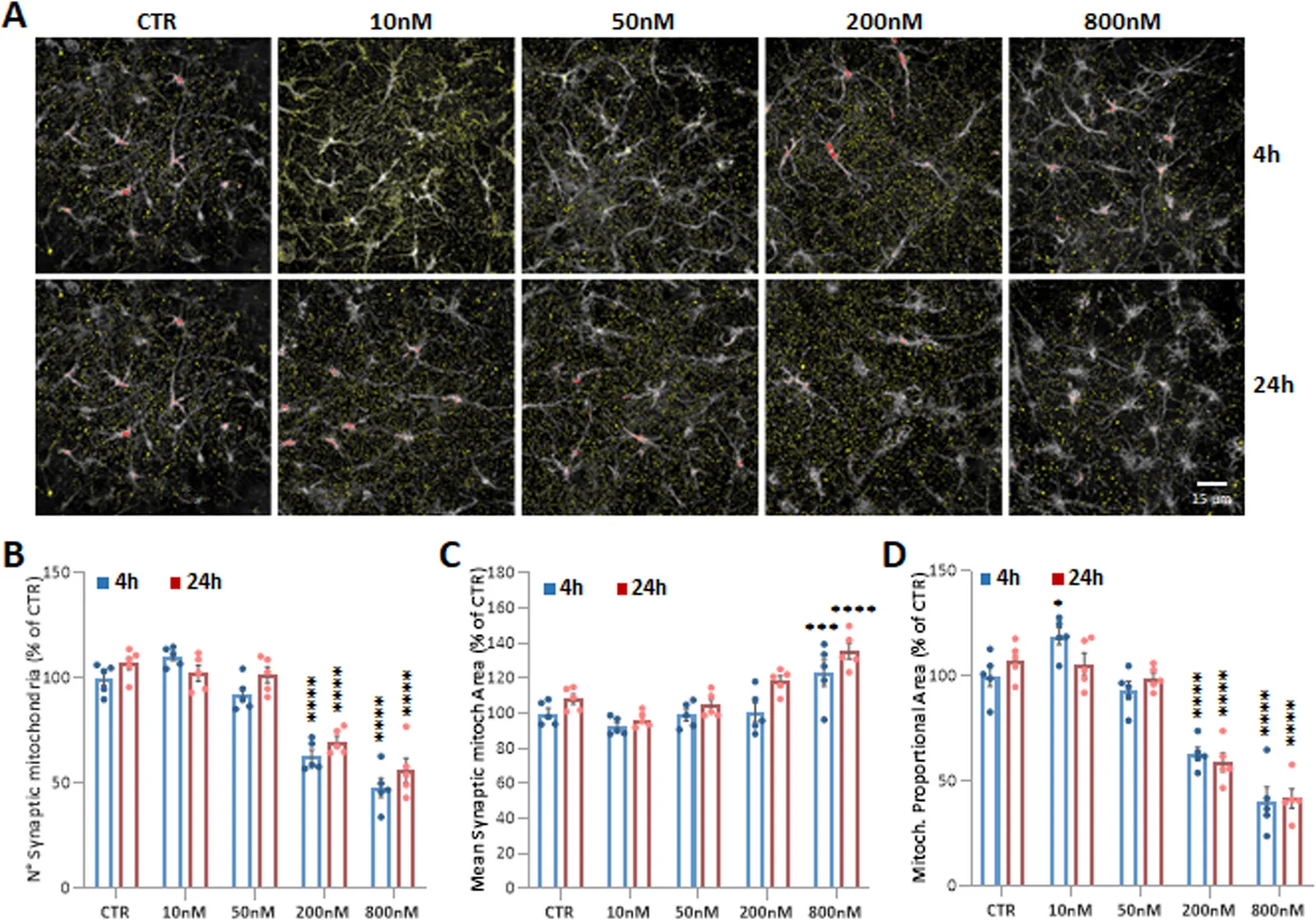
Evaluation of synaptic mitochondria in Rotenone-treated neurons.** A** Representative images of MTG-stained mitochondria and computer-assisted (image analysis system MCID 7.1) determination of synaptic mitochondria in cortical neurons treated with Rot (10, 50, 200, 800 nM) for 4–24 h. **B–D** Computation of total synaptic mitochondria (**B**), mean synaptic mitochondrial area (**C**) and Mitochondrial Proportional Area (mitochondrial occupancy) (**D**) at the synaptic contacts. Data, expressed as a percent of CTR ± SEM, are the mean of five independent samples. ∗ ≤ 0.05, ∗∗ ≤ 0.01, ∗∗∗ ≤ 0.001, ∗∗∗∗ ≤ 0.0001 *versus* their respective CTR (ANOVA and Dunnett’s multiple comparisons test)

Mitochondrial fragmentation was mostly evident along neurites and at the synaptic level. Indeed, insets in Fig. 7A display the progressive transition from an elongated, interconnected network (CTR) to a highly fragmented punctate and “swollen” morphology (50 nM), culminating (200–800 nM) in severe mitochondrial depletion along neuritic processes, which appear as ‘ghost’ neurites almost depleted of organelles (Fig. 7A). Indeed, computer-assisted count of mitochondria at synaptic contacts revealed that toxic concentrations of Rot cause a significant reduction in the number of synaptic mitochondria (Fig. 8A-B). Strikingly, within these depleted synaptic regions (ROIs), the automated evaluation of the mean fragment area showed a clear enlargement of the remaining mitochondria after 800 nM treatment (Fig. 8C). Nevertheless, due to the massive loss in total organelle numbers, this single-fragment swelling was insufficient to preserve overall synaptic coverage, resulting in a dramatic concentration-dependent reduction of the mitochondrial proportional area (mitochondrial occupancy) at the synaptic level (Fig. 8D).

To substantiate the morphological changes observed in terms of mitochondrial networks, we assessed the levels of proteins regulating mitochondrial remodeling in neurons treated with Rot 10-800nM for 3, 6 and 24 h. Surprisingly, we found that neurons treated with subtoxic 10 or 50nM Rot do not exhibit relevant changes in mitochondrial dynamics proteins (Suppl. Figure 3), suggesting that at these low doses, the localized synaptic fragmentation and mitochondrial swelling occur independent of relevant fluctuations in the total dynamics machinery.

**Fig. 9:**
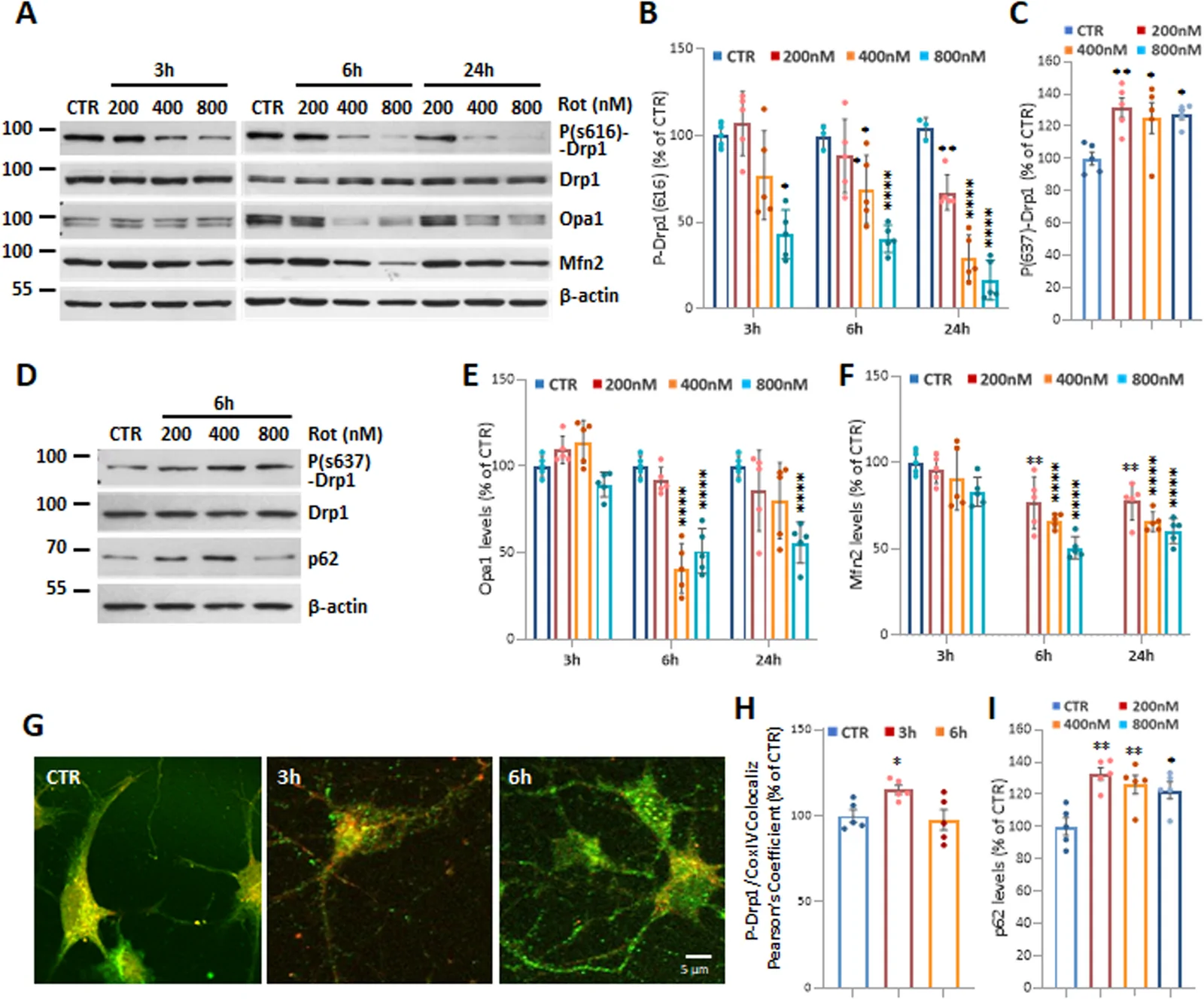
Effect of Rotenone on mitochondrial dynamics in cortical neurons.** A** Representative immunoblots for P(S616)-Drp1, Drp1, Opa1 and Mfn2 in cortical neurons treated with Rot (200, 400, 800nM) for 3–6−24 h. **B–C** Quantification of P(S616)-Drp1/Drp1 (B) and P(S637)-Drp1/Drp1 (C) levels. **D** Representative immunoblots of P(S637)-Drp1, Drp1 and SQSTM1/p62 in lysates from neurons treated with Rot (200, 400, 800 nM) for 6 h. **E–F** Quantification of Opa1 (E) and Mfn2 (F). **G** Representative images of P(S616)-Drp1 (red)/CoxIV (green) colocalization in cortical neurons exposed to Rot (200 nM) for 3–6 h. **H** Quantitation of fluorescence intensity and P-Drp1/CoxIV colocalization by the Pearson’s coefficient of correlation (*r*) measured in about 100 cells for each condition in 10 random fields. **I** Quantification of p62 levels. In all immunoblots, protein levels were normalized by the actin content. All data, expressed as % of CTR, is the mean ± SEM of three experiments with two independent samples. ∗ ≤ 0.05, ∗∗ ≤ 0.01, ∗∗∗ ≤ 0.001, ∗∗∗∗ ≤ 0.0001 *versus* their respective CTR)

Conversely, major changes were evident in neurons challenged with toxic Rot concentrations. Specifically, neurons treated with Rot 200, 400 or 800nM for 3–6−24 h show a dose- and time-dependent decrease in the levels of P(616)-Drp1 (Fig. 9A–B), indicative of reduced mitochondrial fission. These data were complemented by immunoblots showing a concomitant increase in the inhibitory P(S637)-Drp1 levels in neurons treated with Rot for 6 h (Fig. 9C–D). As phosphorylation of Ser616 increases Drp1 activity while phosphorylation of Ser637 decreases it (Archer 2013), these data are consistent with decreased mitochondrial fission. However, immunocytochemical studies revealed that Rot (200 nM) causes a significant increase in the levels of P(S616)-Drp1 localization to mitochondria, as revealed by enhanced P(S616)Drp1/CoxIV overlap (Fig. 9G–H).

Notably, while immunofluorescence microscopy successfully captured this transient, early recruitment of P-Drp1 to mitochondria at 3 h, its localized membrane-bound oligomerization is known to exhibit altered solubility profiles in standard lysis buffers due to the formation of high-molecular-weight membrane complexes (Hu and Qi 2020; Rochon et al. 2024), contributing to the apparent mismatch between in situ single-cell imaging and total protein levels.

Likewise, we found that toxic Rot (200–800 nM) also triggers a substantial reduction in the content of Mfn2 at all time points (Fig. 9A, F). In contrast, Opa1 levels exhibited a distinct, non-linear temporal profile. Densitometric analysis of total Opa1 protein expression revealed a drastic decrease at 6 h followed by a partially attenuated downregulation at 24 h across all concentrations (Fig. 9A and E). To further clarify the processing kinetics of individual pools, a refined parallel quantification was performed specifically on the 100 kDa functional long isoform (L-Opa1), which serves as the primary substrate for stress-induced OMA1 cleavage, confirming this concentration-dependent temporal stabilization (Suppl. Figure 4). This transient reduction at 6 h suggests a stress-induced proteolytic cleavage of Opa1 that impairs inner membrane fusion. The subsequent partial recovery observed at 24 h likely reflects a cell-survival selection bias: i.e., resilient neurons may maintain basal Opa1 levels due to a compensatory transcriptional attempt to restore the fusion machinery, along with a persistent autocatalytic self-cleavage of the mitochondrial protease OMA1 (Fogo et al. 2024).

We previously reported that Rot impairs both CMA and macroautophagy (Sala et al. 2016). Based on the well-established role of autophagy in clearing dysfunctional fragmented mitochondria, we assessed the level of SQSTM1/p62, a scaffold protein involved in (and degraded by) autophagy/mitophagy (Ashrafi and Schwarz 2013). Indeed, we found that neurons treated with Rot (200–800 nM) display higher p62 content at 6 h (Fig. 9D, I) suggesting an impairment of the autophagic flux. Remarkably, this effect appeared slightly attenuated at the highest concentration (800 nM), perhaps due to the onset of acute neurotoxic cellular collapse and subsequent generalized proteolysis before massive cell death, together with limited *de novo* protein synthesis and accumulation of p62.

Together, these data indicate that toxic Rot concentrations (200-800nM) drive a generalized failure of physiological, regulated fission-fusion cycles and mitochondrial quality control, leading to the accumulation of damaged mitochondria that continue to release toxic ROS, thereby exacerbating energetic failure and neuronal demise. Conversely, the biochemical stability maintained at subtoxic doses (10–50 nM) suggests a clear threshold effect, where early localized morphometric adaptations occur independent of major alterations in the core dynamics protein machinery.

## Discussion

The main novelty of the present study resides in the dissection of the early bioenergetic thresholds in primary cortical neurons exposed to subtoxic rotenone. By combining metabolomics with advanced automated High-Content Screening (HCS) morphometric imaging, we provide evidence of a spatial-temporal hierarchy in neuronal injury. Specifically, we unveil that low rotenone concentrations employ complex metabolic rewiring to compensate for bioenergetics impairment, while engaging localized mitochondrial fragmentation and single-fragment swelling at synaptic sites prior to any detectable biochemical alterations of the core fusion/fission protein machinery, offering a novel insight into the earliest latent phases of neurometabolic failure.

### Early Oxidative Stress Precedes Neuronal Death and Mitochondrial Collapse

Consistent with previous reports (Testa et al. 2005; Sala et al. 2016; Eggers et al. 2025), inhibition of mitochondrial Complex I by rotenone produced a rapid increase in total and mitochondrial ROS. Notably, oxidative stress was detectable within 30 min even at subtoxic concentrations (10 nM) that do not immediately compromise neuronal viability (Fig. 1). The concomitant increase in lipid peroxidation further supports the notion that mitochondrial ROS rapidly propagate damage to cellular membranes. Oxidative modification of lipids is particularly relevant in neurons, as membrane integrity is essential for axonal and synaptic transmission. These findings align with evidence from PD patient tissues and experimental models showing elevated lipid peroxidation and oxidative damage as early pathological features (Navarro et al. 2009; Quan et al. 2026).

Our live-imaging analysis then showed that rotenone induces an early and progressive loss of mitochondrial membrane potential (Δψm), initially affecting mitochondria in neurites and later extending to the soma (Fig. 2). Importantly, preliminary morphometric control analysis confirmed that the average neuronal somatic area remained statistically stable and unchanged across all tested rotenone concentrations and timepoints compared to control cells (Suppl. Figure 1), thereby ruling out cell shrinkage or swelling artifacts in our bioenergetics and fluorescence quantifications.

This spatial vulnerability is consistent with the high energy demand of neuronal processes and the reliance of synaptic function on local mitochondrial ATP production (Rangaraju et al. 2014; Pathak et al. 2015; Kilbride et al. 2021; Hu et al. 2025) The functional consequences of Δψm loss were confirmed by the bioenergetic profiles showing a dramatic reduction in basal respiration, maximal respiration and ATP-linked respiration (Fig. 3). Remarkably, all experimental conditions revealed a significant decrease in the spare respiratory capacity, which is a critical determinant of neuronal resilience, i.e. the capacity of cells to respond to increased energetic demands or stress: its early collapse at low rotenone concentrations indicates that neurons rapidly lose metabolic flexibility and become highly susceptible to further damage. The marked reduction in ATP levels further underscores the severity of mitochondrial impairment, in line with previous studies on PD models based on Complex I inhibition (Testa et al. 2005; Sala et al. 2016).

The temporal discrepancy between the early disruption of mitochondrial respiration and the delayed depletion of steady-state ATP pools highlights a critical homeostatic buffering phase in primary cortical neurons. Under acute Complex I restriction (50 nM for 3 h), neurons might transiently sustain total ATP levels by rapidly mobilizing cell reserves (such as phosphocreatine reserves) and accelerating cytosolic glycolytic flux. However, this metabolic buffering is temporary and becomes completely exhausted by 6 h, as evidenced by the subsequent sharp decline in ATP.

Conversely, under a milder bioenergetic challenge (10 nM), neurons maintain a temporary energetic plateau during the acute phase (3–6 h). As the insult progresses to 24 h, cumulative mitochondrial dysfunction may eventually trigger a ~ 50% drop in ATP. Crucially, this state stabilizes into a survival plateau rather than proceeding to immediate cell death, a phenomenon dynamically supported by our parallel metabolomics validation. The targeted accumulation of dihydroxyacetone phosphate (DHAP) and glycerol-3-phosphate (Gly-3P) at 24 h confirms that surviving neurons actively rewire their metabolism, engaging a compensatory upregulation of the Gly-3P shuttle and downstream glycolysis to escape acute bioenergetic collapse.

### Metabolic Rewiring Reflects an Unsuccessful Attempt to Compensate for Mitochondrial Failure

Metabolomic analysis in neurons exposed to 10nM Rot revealed a widespread disruption of energy-related pathways including glycolysis, TCA cycle, malate–aspartate shuttle and pyruvate metabolism (Figs. 4 and 5). The initial increase in TCA intermediates at 24 h may suggest an ongoing process of partial metabolic adaptation that is not maintained under chronic conditions (3–7 days). In addition, our metabolomic analysis newly indicates how the transient increase in components of the Gly-3P shuttle might be an intriguing compensatory mechanism to regenerate NAD^+^ from NADH and foster glycolysis, while bypassing Complex I inhibition through succinate dehydrogenase (Complex II)-mediated oxidation of the FADH_2_ produced by mitochondrial Gly-3P dehydrogenase. Together with the malate-aspartate shuttle, the Gly-3P shuttle has been recently reported as a system preserving metabolic flexibility in hippocampal neurons (Dhoundiyal et al. 2022).

While reduction of glycolysis and TCA cycle have been detected in other cellular models based on higher rotenone concentrations (Suciu et al. 2023), the involvement of the Gly-3P shuttle observed in our neuronal model of chronic low nanomolar rotenone might be regarded as an interesting compensatory mechanism that is likely to occur at early stages of the pathological context observed in PD (Schapira et al. 1989). On the other hand, the transient metabolic shift to glycolysis suggests a transition toward less efficient, partially anaerobic energy production, reminiscent of metabolic changes reported in PD brains and cellular models (Chen et al. 2025; Shen et al. 2025). However, this adaptation response appears insufficient to restore ATP levels, indicating that metabolic rewiring cannot fully overcome mitochondrial dysfunction.

In addition to its role in energy production, the transient rise in Gly-3P might be also linked to its fundamental role as building block for the synthesis of glycolipids and phospholipids which, together with the MVA pathway, are likely involved in the temporary preservation of membrane structure and integrity but lost after extended Rot treatments.

Moreover, we newly show that complex I inhibition and reduced ATP production elicit a persistent downregulation of all anabolic processes, as indicated by the decrease in amino sugar metabolism and folate/1C-metabolism with the significant decrease in L-serine/L-glycine, together with reduced L-methionine levels and L-methionine/homocysteine ratio (Fig. 6). Increased homocysteine levels have been found to correlate with cortical structural damage in PD subjects and suggested to be relevant in the development and progression of PD (Fan et al. 2020; Sampedro et al. 2022).

### Redox Imbalance and Impaired Glutathione Synthesis Exacerbate Neuronal Vulnerability

Our data also revealed substantial alterations in redox homeostasis, including a persistent reduction in the NAD⁺/NADH ratio, as previously reported (Sarkar et al. 2023), as well as riboflavin levels. Particularly striking is the dysregulation of glutathione (GSH) metabolism. GSH metabolism is the most downregulated pathway at all timepoints (Fig. 5). Moreover, GSH levels decline without a corresponding increase in oxidized GSSG, suggesting a potential impairment in *de novo* synthesis. The consistent reduction in γ-glutamyl cysteine, the rate-limiting intermediate in GSH synthesis, strongly supports this interpretation and aligns with previous metabolomic studies in PD models (Huang et al. 2023).

Alterations in glutamate and cysteine metabolism also suggest impaired cystine uptake and might be indicative of altered system xc−, the antiporter exchanging intracellular glutamate (1:1) with extracellular cystine (then intracellularly reduced to cysteine), essential in maintaining the redox balance and GSH synthesis. Reduction in the efficacy of the system xc − due to its degradation by calpains and mechanisms of oxidative glutamate toxicity have been previously reported in SH-SY5Y neuroblastoma cells (Knaryan et al. 2014) and other models of neurodegeneration (Cirillo et al. 2011).

Finally, the strong downregulation in glutamate and Proline/Arginine metabolism at 3–7 days are connected to reduced ammonia recycling and urea cycle (Fig. 5), mechanisms to detoxify the ammonia produced during neurotransmitter metabolism, as previously reported in other models of neurodegeneration (Leke et al. 2011). Moreover, alteration in the Urea cycle has been recently associated with neuronal dysfunction and metabolic stress in PD (Zhang et al. 2025).

### Disruption of Mitochondrial Dynamics and Quality Control Amplifies Mitochondrial Dysfunction

Metabolic rewiring is tightly connected to mitochondrial dynamics (Westermann 2010, 2012; Chen et al. 2023; Goglia et al. 2024). Beyond functional and metabolic impairments, rotenone severely altered mitochondrial morphology and distribution.

High content imaging and automated morphometric quantitation revealed that Rot-treated neurons exhibit an increase in spherical ‘punctate’ mitochondria (0.5–3 µm diameter) (Fig. 7), indicating that mitochondrial networks fracture into small spherical puncta which undergo swelling and dispersion, a hallmark of severe bioenergetic stress (Anzell et al. 2021). Importantly, this structural disintegration is extremely rapid (4 h) under toxic rotenone concentrations (Fig. 7A), whereas the subtoxic 10 nM Rot produces swollen mitochondria through a delayed, progressive process that manifests only at 24 h (Fig. 7C–E). Moreover, this structural remodeling occurred primarily along neurites and at the synaptic regions, producing ‘ghost’ neurites emptied of mitochondria (Fig. 7A), along with a concurrent loss of synaptic organelle occupancy (Fig. 8D), in agreement with the notion that synaptic dysfunction is an early event in PD pathogenesis.

At the molecular level, subtoxic rotenone doses did not cause significant changes in the total expression of proteins regulating mitochondrial dynamics (Suppl. Figure 3), consistent with a concentration-dependent threshold effect where biochemical degradations may occur later in the pathological cascade. This discrepancy highlights a threshold-dependent spatial and temporal hierarchy in neuronal injury: low doses (10–50 nM) trigger immediate functional bioenergetic stress (Fig. 3) that drives metabolic rewiring (Figs. 4, 6 and 5) and local, sub-cellular structural remodeling (i.e., network fragmentation into small spherical puncta and synaptic organelle depletion) without affecting the cellular pools of dynamics proteins, suggesting that these localized morphometric adaptations may precede whole-cell biochemical degradation.

Crucially, the observation that low nanomolar rotenone (10–50 nM) triggers localized mitochondrial network fragmentation and single-fragment swelling without altering the total whole-cell protein levels of P-Drp1, Opa1, or Mfn2 suggests a dual mechanism operating at subtoxic thresholds. On one hand, the initial bioenergetic failure, ATP depletion, and local ROS propagation can directly disturb the organelle’s osmotic balance, promoting matrix swelling (Zorov et al. 2014); this physical distension can induce passive, Drp1-independent fragmentation of the fragile neuritic network, as observed when post-mitotic neurons undergo localized oxidative stress driven by elevated mitochondrial ROS production (Kageyama et al. 2012; Yamashita et al. 2016), ultimately leading to the structural swelling cascades described during early neurometabolic insults in cortical neurons (Anzell et al. 2021). On the other hand, this delayed kinetic profile and the preservation of the core dynamics machinery suggest the activation of a mitohormesis-like response. At these subtoxic concentrations, the mild and compartmentalized neurometabolic stress may act as an adaptive, compensatory stimulus, driving localized mitochondrial remodeling to temporarily optimize metabolic resilience and maintain neuronal survival.

Instead, severe dysregulation of fission-fusion cycles and mitochondrial quality control becomes detectable only in response to toxic rotenone concentrations (starting from 200 nM and fully manifesting at 400–800 nM). Toxic doses reduced phosphorylation of Drp1 at Ser616 (Fig. 9B) while increasing the inhibitory P(Ser637)-Drp1 (Fig. 9C-D), suggesting a suppression of regulated mitochondrial fission. Remarkably, however, immunofluorescence microscopy successfully captured a transient recruitment of P-Drp1 to the mitochondrial network at 3 h (Fig. 9G-H), thus revealing an apparent mismatch between in situ single-cell imaging and total protein levels. This effect is likely due to the altered solubility of localized membrane-bound P-Drp1 oligomers in standard lysis buffers, where they form high-molecular-weight complexes (Hu & Qi 2020; Rochon et al. 2024).

Intriguingly, toxic rotenone also caused a reduction in the mitochondrial fusion proteins Mfn2 (Fig. 9F) and Opa1 (Fig. 9E). Opa1 levels in particular exhibited a distinct temporal profile, showing a robust, concentration-dependent downregulation that reached its maximum during the acute bioenergetic crisis at 6 h (Fig. 9A and E). Conversely, this mitochondrial collapse appeared partially attenuated at 24 h across toxic concentrations (Fig. 9E), a kinetic trend systematically validated when focusing specifically on the 100 kDa functional long isoform (Opa1) pool (Suppl. Figure 4).

Notably, the concurrent downregulation of both key fusion mediators, Mfn2 and Opa1, suggests a generalized collapse of physiological, regulated mitochondrial dynamics. Rather than a selective, active activation of the fission machinery, this simultaneous suppression of both fusion and fission pathways appears to trigger a passive disgregation of the network. Under these conditions, individual mitochondria lack the capacity to engage in dynamic remodeling, thereby succumbing to the localized fragmentation and osmotic swelling observed in our live-imaging screens.

This coordinated failure might prevent the homeostatic maintenance of the network, trapping dysfunctional organelles in an un-recyclable state. Because balanced fission–fusion cycles are strictly required to isolate damaged mitochondria for targeted mitophagic removal, this structural “freeze” may critically impair mitochondrial quality control, promoting the retention of damaged mitochondria that continue to produce ROS, exacerbate energetic failure, and drive final neuronal demise.

Consistent with this interpretation, we observed a marked accumulation of p62 (Fig. 9I), formalizing a severe impairment in downstream autophagic/mitophagic clearance. These findings align with our previous evidence demonstrating that rotenone disrupts the coordination between CMA and macroautophagy (Sala et al. 2016). Intriguingly, p62 expression exhibits a distinctly non-linear concentration profile at the highest neurotoxic threshold. This slight compression or plateau of steady-state p62 levels at 800 nM may reflect a complex intersection of competing proteolytic networks operating under extreme bioenergetic stress. Just as Opa1 temporal kinetics are shaped by the fine-tuned autocatalytic feedback of OMA1 (Fogo et al. 2024), steady-state p62 pools are dynamically regulated by the concurrent activity of macroautophagy and the ubiquitin-proteasome system (UPS) (Shin et al. 2020). Crossing the 800 nM threshold likely triggers shifting homeostatic waves where these distinct degradative pathways overlap and collapse non-linearly. This metabolic collapse may also shut down *de novo* protein synthesis, while simultaneously activating executioner proteases (such as calpains and caspases) during the early phases of cell death. This cross-talk amplifies the biological heterogeneity of steady-state protein levels, which characteristically oscillate between persistent accumulation and partial catabolic breakdown, without representing a functional reactivation of regulated autophagic flux. Collectively, these findings move far beyond a descriptive analysis of mitochondrial injury, establishing a highly refined molecular blueprint of the active defense phases and terminal execution cascades of primary cortical neurons.

### Implications for Parkinson’s Disease Pathogenesis

Altogether, our data supports a model in which mitochondrial Complex I inhibition initiates a cascade of events involving oxidative stress, metabolic rewiring (or collapse), impaired mitochondrial dynamics, and defective quality control. Considering that low nanomolar rotenone may reproduce more closely the initial stages of mitochondrial impairment in PD, the extensive metabolic rewiring observed here is likely to mirror alterations reported in PD patient tissues, including impaired glucose metabolism, redox imbalance and altered amino acid homeostasis. These similarities reinforce the relevance of our findings to PD pathophysiology and suggest that targeting mitochondrial metabolism, redox balance, and mitochondrial dynamics may represent effective strategies to delay or prevent neuronal degeneration.

In conclusion, our study reveals that rotenone-induced mitochondrial dysfunction extends far beyond simple respiratory inhibition, encompassing coordinated alterations in metabolism, redox homeostasis, and mitochondrial architecture. These interconnected defects likely cooperate to drive neuronal vulnerability and degeneration, providing insight into early pathogenic mechanisms in Parkinson’s disease.

### Study Limitations and Translational Relevance

Finally, some limitations of the present study must be acknowledged. First, our experiments were conducted on primary cortical neurons. While these cultures represent a well-established and robust model to dissect intrinsic intracellular and mitochondrial pathways under controlled conditions, their neurochemical and metabolic profile differ significantly from mature, adult neurons. Furthermore, in Parkinson’s Disease pathology, dopaminergic neurons of the substantia nigra exhibit a unique vulnerability to oxidative stress and complex I inhibition due to pacemaking activity and dopamine metabolism. Therefore, while our findings on localized mitochondrial swelling and transient glycolytic compensation provide fundamental insights into early neurotoxic thresholds, caution should be exercised when directly extrapolating these neurometabolic outcomes to long-term, cell-specific neurodegenerative processes in adult human patients. Future studies exploiting mature human iPSC-derived dopaminergic platforms will be essential to translate these bioenergetic cascades into clinical relevance.

Furthermore, it must be acknowledged that the present study is deliberately restricted to Complex I inhibition via a rotenone paradigm. While comparing these outcomes with Complex II or Complex III blockers (e.g., 3-nitropropionic acid or antimycin A) would offer broad comparative bioenergetic insights, different respiratory chain inhibitors possess deeply distinct intra-neuronal uptake kinetics, mechanisms of action, and enzymatic inhibition constants (Ki). Consequently, establishing biologically equivalent respiratory restrictions across different complexes would require an independent, large-scale kinetic and dose–response calibration screening, which falls beyond the specific scope of the present work.

## Supplementary Information

Below is the link to the electronic supplementary material.

Supplementary Material 1

## Data Availability

The data that support the findings are available from the corresponding author upon reasonable request.

## Abbreviations

Ac-CoA: Acetyl-CoA
αKG: α-Ketoglutarate
BODIPY: BODIPY™ 581/591 C11
Citr: Citrate
CMA: Chaperone-mediated autophagy
DHAP: Dihydroxyacetone phosphate
DMG: Dimethylglycine
Drp1: dynamin-related protein 1
ER: Enriched Ratio
Fum: Fumarate
γGC: γ-glutamyl cysteine
GGPP: Geranylgeranyl diphosphate
GlcN6P: Glucosamine-6-phosphate
GlcNAc6P: N-acetylglucosamine 6-phosphate
Gly-3P: Glycerol 3-phosphate
GPP: Geranyl diphosphate
GSH: reduced glutathione
GSSG: oxidized glutathione
H2DCF-DA: Dihydro-dichlorofluorescein diacetate
Hcy: homocysteine
L-Arg: L-Arginine
L-Cys: L-Cysteine
L-Gly: L-Glycine
L-Glu: L-Glutamate
L-Gln: L-Glutamine
L-Mal: L-Malate
L-Met: L-methionine
L-Pro: Proline
L-Ser: L-Serine
Mfn2: Mitofusin-2
MitoSOX: MitoSOX™ Red
MTG: MitoTrackerTM green FM
MTR: MitoTracker red CMXRos
MVA: Mevalonate
MVA-P: Mevalonate-P
p62: SQSTM1/p62 (Sequestosome-1)
Pyr: Pyruvate
ROS: Reactive oxygen species
ROT: Rotenone
Ru5P: D-Ribulose 5-phosphate
S7P: Sedoheptulose 7-phosphate
Succ: Succinate
TMRE: Tetramethylrhodamine
UDP-GlcNAc: Uridine diphosphate N-acetylglucosamine
5,10-CH2-THF: 5,10-methylene-THF
