# Supplementary material for: Early Metabolic Rewiring and Mitochondrial Remodeling in Response to Mitochondrial Impairment in a Rotenone Model of Parkinson’s Disease
Source: Cell Mol Neurobiol. 2026 Aug 12;46:162. doi: 10.1007/s10571-026-01794-8 (PMC13745613; doi:10.1007/s10571-026-01794-8)

Early metabolic rewiring and mitochondrial remodeling in response to mitochondrial impairment in a rotenone model of Parkinson's disease

Cellular and Molecular Neurobiology

Alissa Costantini[1†], Marcella Bonanomi[2†], Gessica Sala[3,5†*], Assunta Virtuoso[4], Anastasia Rella[1], Martina Motto[1], Claudio Gioia[1], Gabriele Taverna[1], Francesca Martorana[1], Federica Aprea[1], Matteo Formenti[1], Michele Papa[4], Carlo Ferrarese[3,5], Daniela Gaglio[2], Anna Maria Colangelo[1,5*]

* Corresponding authors: Prof Anna Maria Colangelo, Laboratory of Neuroscience "R. Levi-Montalcini", Dept. of Biotechnology and Biosciences, University of Milano-Bicocca, piazza della Scienza 2, 20126 Milano, Italy. Phone (+39) 02 64483536; Email: annamaria.colangelo@unimib.it orcid.org/0000-0002-7971-4289; Dr. Gessica Sala, Laboratory of Neurobiology, School of Medicine and Surgery, University of Milano-Bicocca, Monza, Italy

Supplementary Information

Supplementary Figure 1

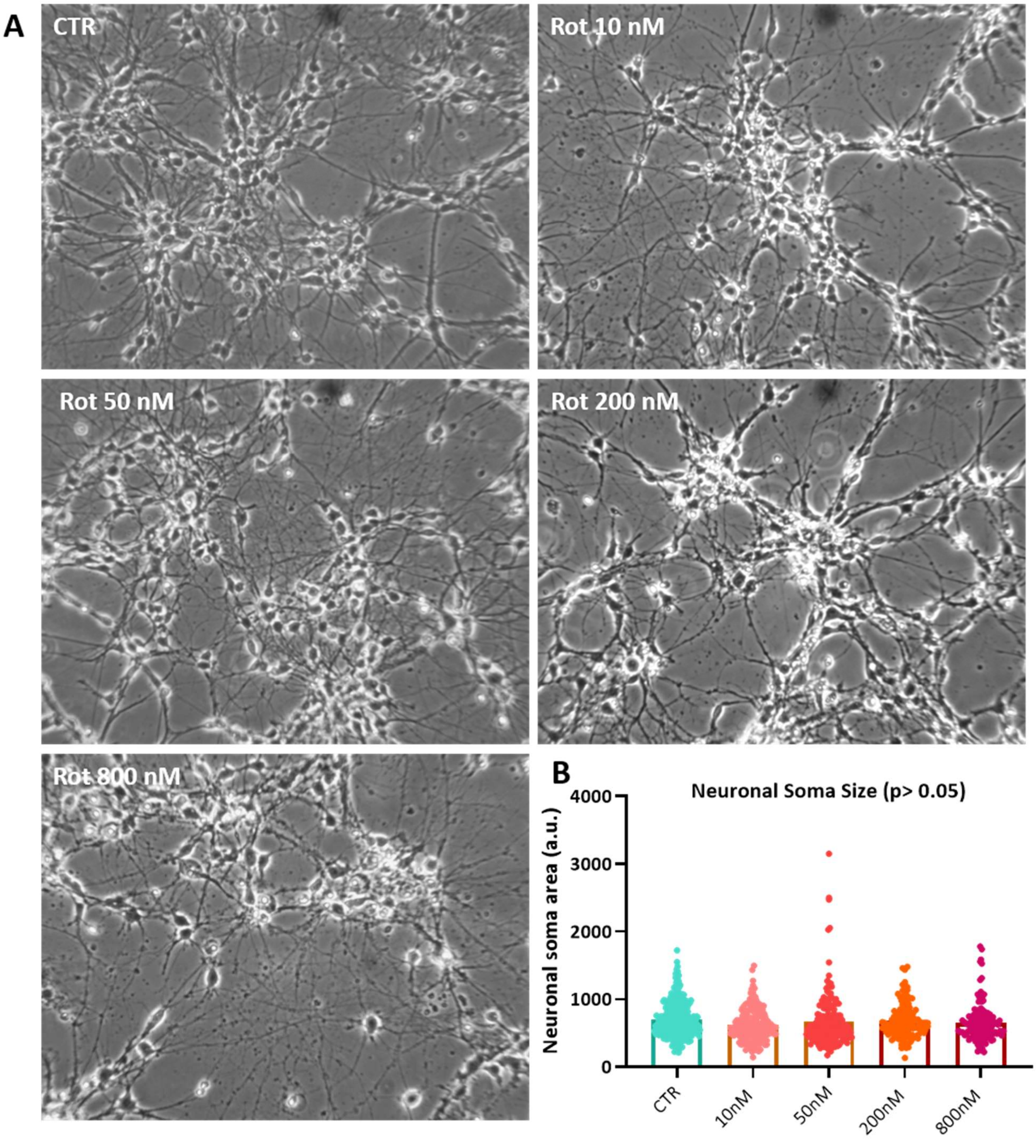

Suppl. Fig 1. Neuronal somatic area remains stable under nanomolar rotenone treatments. **A**) Representative phase-contrast images of primary cortical neurons captured before and after treatments across the different experimental conditions. **B**) Morphometric analysis of neuronal soma size. Individual neuronal cell bodies (80–120 neurons per condition) were manually traced using the free-hand selection tool in ImageJ (Fiji). Data are presented as a bar graph with individual data points representing single cells. Statistical analysis was performed using One-Way ANOVA on the means of 3-4 independent samples across all conditions, showing no statistically significant differences between control (CTR) and rotenone-treated groups ($p > 0.05$).

Supplementary Figure 2

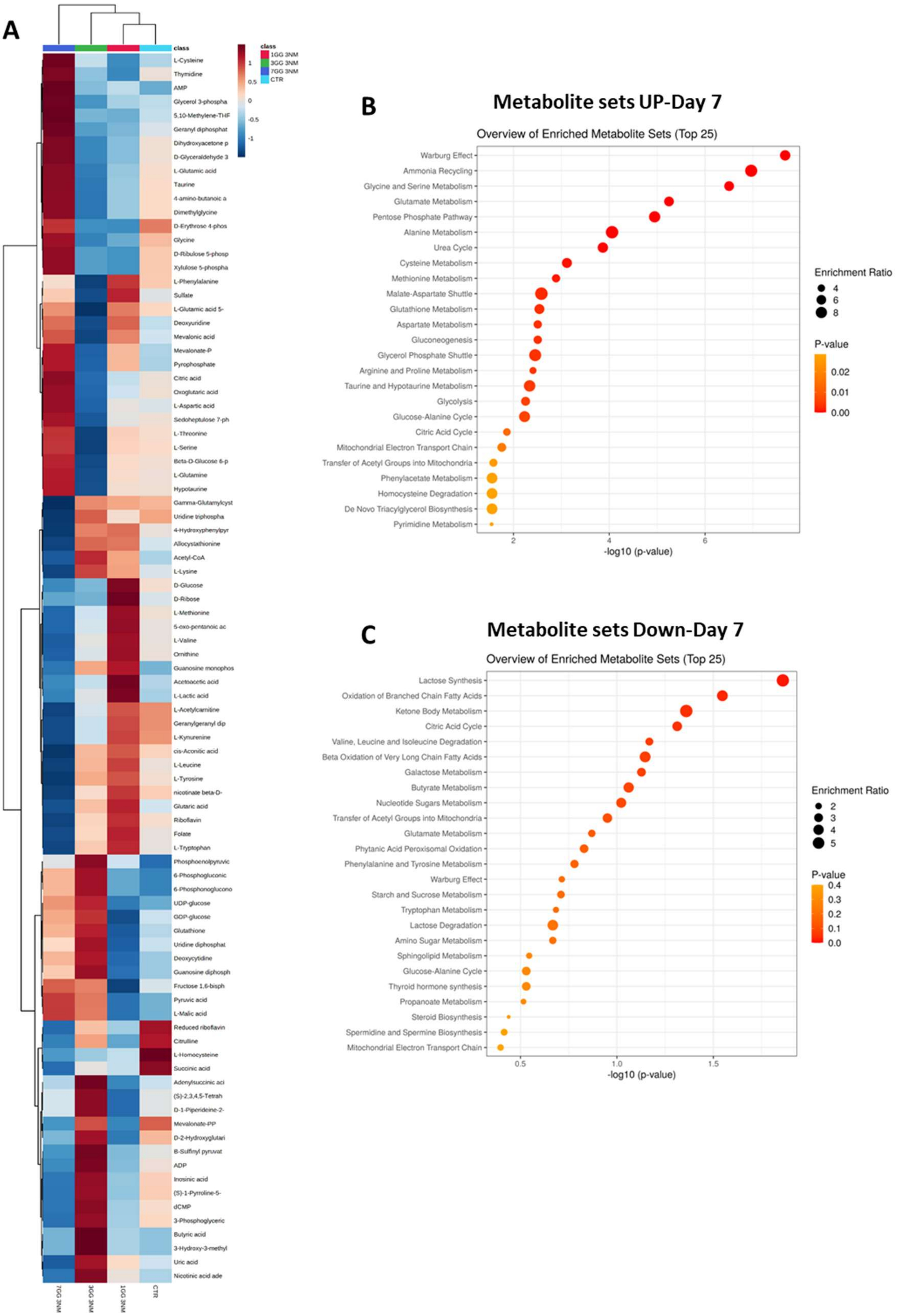

Suppl. Fig 2. Metabolic Phenotype of cortical neurons under chronic 3nM Rotenone Treatment. **A**) Hierarchical clustering heatmap showing significantly different intracellular metabolites by LC-MS in cortical neurons treated with Rot 3nM for 24h or 3-7 days (Color scale: from blue to red). **B-C**) Enriched metabolites pathways in cortical neurons treated with Rot (3 nM) on day-7 (up- and downregulated, respectively) showing

the enriched metabolic pathways and the corresponding enrichment ratios (ER) and P-value (Color scale: light to dark red).

Supplementary Figure 3

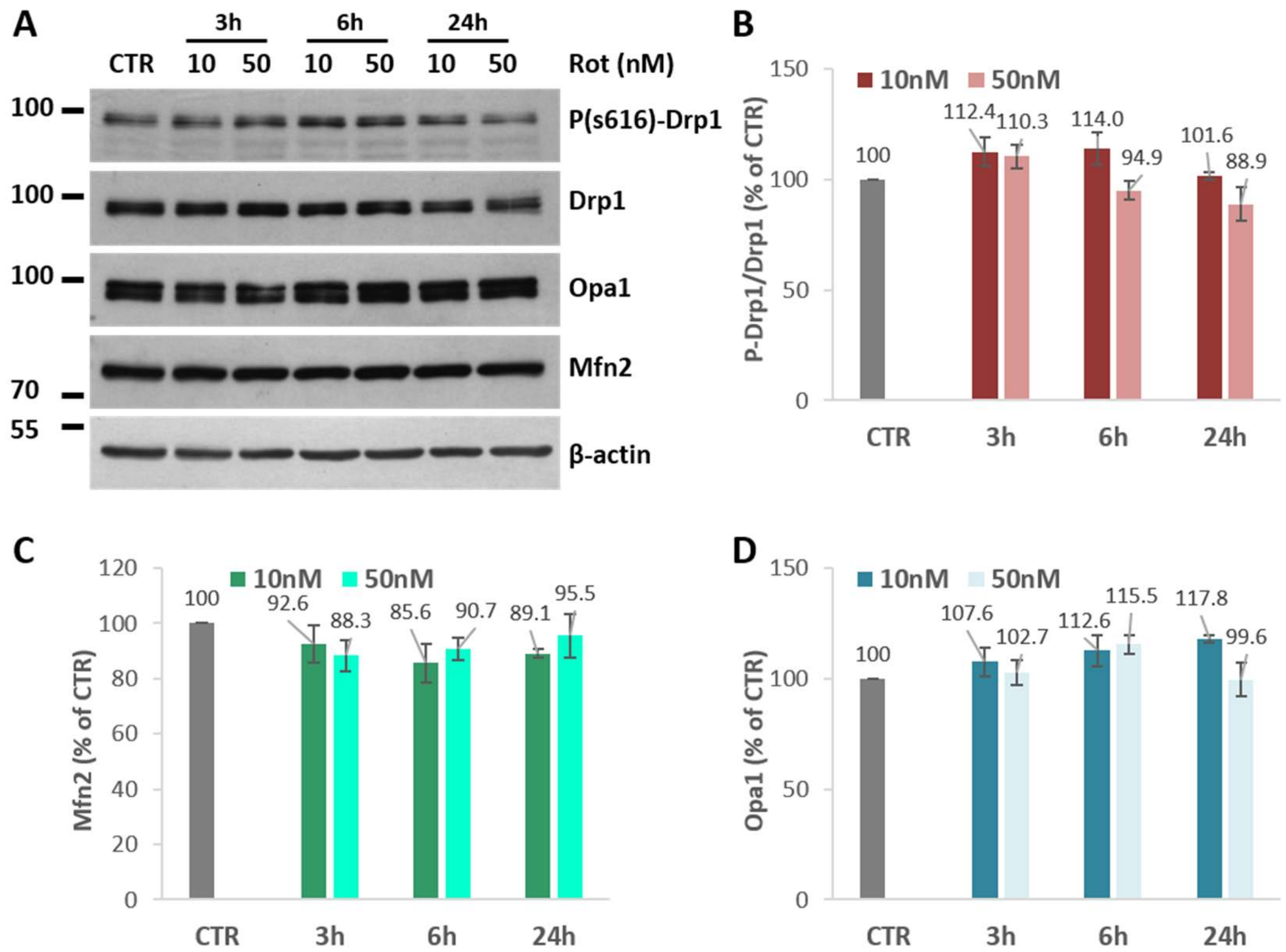

Suppl. Fig 3. Quantitative and qualitative baseline assessment of core mitochondrial dynamics proteins under sub-toxic rotenone exposure. (**A**) Representative immunoblots for P(Ser616)-Drp1, total Drp1, OPA1, and Mfn2 in primary cortical neurons treated with low nanomolar Rot (10 and 50 nM) for 3, 6, and 24h. β-actin was used as internal loading control. (**B–D**) Densitometric quantification profiles expressing individual protein targets as % of CTR. Data bars represent the mean of biological duplicates (N=2) performed as a preliminary quality control threshold screen, with error bars indicating the range (±SE). These integrated screens demonstrate a stable baseline profile of the core fusion and fission machinery under sub-toxic treatments, in contrast with the marked significant degradation cascades triggered exclusively above the neurotoxic threshold (200 nM; Figure 9).

Supplementary Figure 4

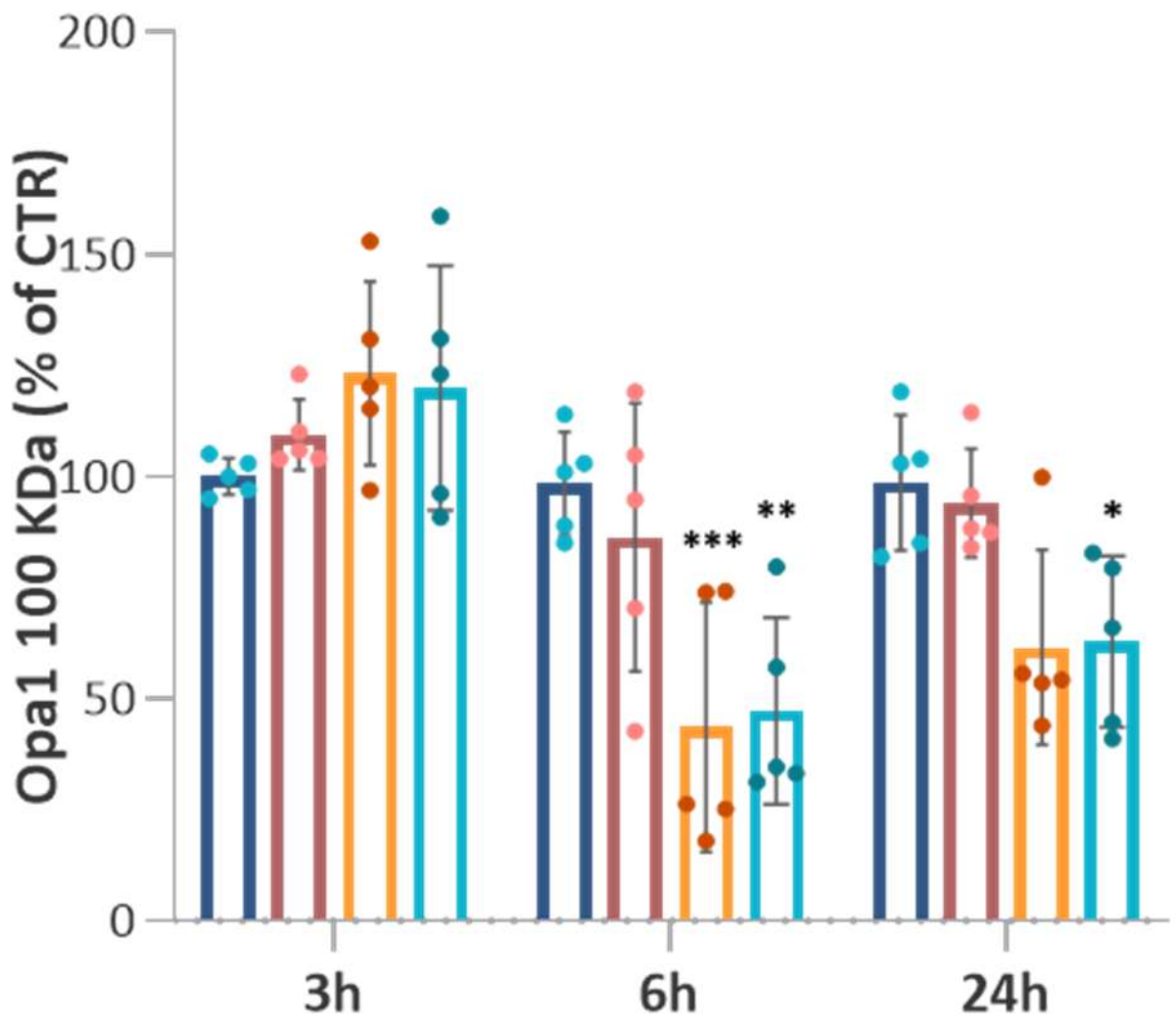

Suppl. Fig 4. Temporal and concentration-dependent profile of the 100-kDa Opa1 functional long isoform (L-Opa1) in primary cortical neurons. Densitometric quantification of the specific 100-kDa long molecular weight band (L-Opa1), corresponding to the primary cleavage substrate of stress-induced mitochondrial proteases. Primary cortical neurons were treated with vehicle (CTR) or neurotoxic concentrations of Rotenone (Rot: 200, 400, 800 nM) for 3, 6, and 24h. Data are expressed as a percentage of the respective control (% of CTR) and represent the mean ± SEM of five independent biological replicates. Statistical analysis was performed via Two-way ANOVA and Dunnet test; *p < 0.05, **p < 0.01, ***p < 0.001 *vs* CTR. The temporal profile reveals a maximal acute structural collapse of the functional L-Opa1 pool at 6h, followed by a partially attenuated, stable chronic stabilization at 24h within the resilient surviving neuronal subpopulation.

Results of Normality tests and other Statistical analysis

Statistics on cell viability by MTT assay

Normality and Lognormality Tests<br>Tabular results	A<br>CTR	B<br>10-6h	C<br>50-6h	D<br>200-6h	E<br>400-6h	F<br>800-6h	G<br>CTR 24h	H<br>10-24h	I<br>50-24h	J<br>200-24h	K<br>400-24h	L<br>800-24h
**Test for normal distribution**												
**Anderson-Darling test**												
A2*	0.3312	0.2010	0.5980	0.3844	0.3738	0.4988	0.4248	0.6592	0.3939	0.3008	0.5759	0.3428
P value	0.4169	0.8161	0.0779	0.3015	0.3217	0.1452	0.2342	0.0526	0.2843	0.5020	0.0898	0.3888
Passed normality test (alpha=0.05)?	Yes	Yes	Yes	Yes	Yes	Yes	Yes	Yes	Yes	Yes	Yes	Yes
P value summary	ns	ns	ns	ns	ns	ns	ns	ns	ns	ns	ns	ns
**D'Agostino & Pearson test**												
K2	2.132	0.4131	1.729	0.4595	3.987	5.072	0.6226	2.283	0.5555	1.350	1.655	0.9130
P value	0.3444	0.8134	0.4212	0.7947	0.1362	0.0792	0.7325	0.3194	0.7575	0.5091	0.4371	0.6335
Passed normality test (alpha=0.05)?	Yes	Yes	Yes	Yes	Yes	Yes	Yes	Yes	Yes	Yes	Yes	Yes
P value summary	ns	ns	ns	ns	ns	ns	ns	ns	ns	ns	ns	ns
**Shapiro-Wilk test**												
W	0.9109	0.9780	0.8479	0.9069	0.8954	0.8588	0.9224	0.8380	0.9070	0.9342	0.8287	0.9195
P value	0.3607	0.9521	0.0907	0.3330	0.2622	0.1169	0.4496	0.0718	0.3336	0.5551	0.0575	0.4255
Passed normality test (alpha=0.05)?	Yes	Yes	Yes	Yes	Yes	Yes	Yes	Yes	Yes	Yes	Yes	Yes
P value summary	ns	ns	ns	ns	ns	ns	ns	ns	ns	ns	ns	ns
**Kolmogorov-Smirnov test**												
KS distance	0.2083	0.1514	0.2721	0.2322	0.1928	0.2281	0.2463	0.2859	0.1915	0.1909	0.2324	0.1930
P value	>0.1000	>0.1000	0.0832	>0.1000	>0.1000	>0.1000	>0.1000	0.0531	>0.1000	>0.1000	>0.1000	>0.1000
Passed normality test (alpha=0.05)?	Yes	Yes	Yes	Yes	Yes	Yes	Yes	Yes	Yes	Yes	Yes	Yes
P value summary	ns	ns	ns	ns	ns	ns	ns	ns	ns	ns	ns	ns
**Number of values**	8	8	8	8	8	8	8	8	8	8	8	8

2way ANOVA<br>Multiple comparisons					
Within each column, compare rows (simple effects within columns)					
Number of families	2				
Number of comparisons per family	5				
Alpha	0.05				
**Dunnett's multiple comparisons test**	**Mean Diff.**	**95.00% CI of diff.**	**Significant?**	**Summary**	**Adjusted P Value**
6h					
CTR vs. Rot 10nM	4.919	-8.735 to 18.57	No	ns	0.8228
CTR vs. Rot 50nM	13.28	-0.3708 to 26.94	No	ns	0.0594
CTR vs. Rot 200nM	19.57	5.920 to 33.23	Yes	**	0.0020
CTR vs. Rot 400nM	22.96	9.303 to 36.61	Yes	***	0.0002
CTR vs. Rot 800nM	26.44	12.78 to 40.09	Yes	****	<0.0001
24h					
CTR vs. Rot 10nM	-1.761	-15.41 to 11.89	No	ns	0.9971
CTR vs. Rot 50nM	15.97	2.315 to 29.62	Yes	*	0.0157
CTR vs. Rot 200nM	19.39	5.740 to 33.05	Yes	**	0.0022
CTR vs. Rot 400nM	41.95	28.29 to 55.60	Yes	****	<0.0001
CTR vs. Rot 800nM	61.29	47.64 to 74.95	Yes	****	<0.0001

Test details	of diff.	N1	N2	q	DF
6h					
CTR vs. Rot 10nM	29	8	8	0.9230	84.00
CTR vs. Rot 50nM	29	8	8	2.492	84.00
CTR vs. Rot 200nM	29	8	8	3.673	84.00
CTR vs. Rot 400nM	29	8	8	4.308	84.00
CTR vs. Rot 800nM	29	8	8	4.960	84.00
24h					
CTR vs. Rot 10nM	29	8	8	0.3305	84.00
CTR vs. Rot 50nM	29	8	8	2.996	84.00
CTR vs. Rot 200nM	29	8	8	3.639	84.00
CTR vs. Rot 400nM	29	8	8	7.871	84.00
CTR vs. Rot 800nM	29	8	8	11.50	84.00

Statistics on total ROS

Normality and Lognormality Tests<br>Tabular results	A<br>CTR	B<br>10nM 30m	C<br>10nM 3h	D<br>10nM 6h	E<br>10nM 24h	F<br>50nM 30m	G<br>50nM 3h	H<br>50nM 6h	I<br>50nM 24h	J<br>200nM 30m	K<br>200nM 3h	L<br>200nM 6h	M<br>200nM 24h
**Shapiro-Wilk test**													
W	0.9048	0.8766	0.9017	0.9585	0.9176	0.9505	0.7981	0.8830	0.8982	0.9281	0.8717	0.8972	0.9384
P value	0.4032	0.2538	0.3840	0.8083	0.4884	0.7446	0.0565	0.2830	0.3631	0.5656	0.2331	0.3577	0.6467
Passed normality test (alpha=0.05)?	Yes	Yes	Yes	Yes	Yes	Yes	Yes	Yes	Yes	Yes	Yes	Yes	Yes
P value summary	ns	ns	ns	ns	ns	ns	ns	ns	ns	ns	ns	ns	ns
**Kolmogorov-Smirnov test**													
KS distance	0.2452	0.3069	0.2322	0.2365	0.2083	0.1918	0.2826	0.2357	0.2255	0.2478	0.2132	0.2080	0.1807
P value	>0.1000	0.0804	>0.1000	>0.1000	>0.1000	>0.1000	>0.1000	>0.1000	>0.1000	>0.1000	>0.1000	>0.1000	>0.1000
Passed normality test (alpha=0.05)?	Yes	Yes	Yes	Yes	Yes	Yes	Yes	Yes	Yes	Yes	Yes	Yes	Yes
P value summary	ns	ns	ns	ns	ns	ns	ns	ns	ns	ns	ns	ns	ns
**Number of values**	6	6	6	6	6	6	6	6	6	6	6	6	6

Ordinary one-way ANOVA<br>Multiple comparisons							
Number of families	1						
Number of comparisons per family	12						
Alpha	0.05						
**Dunnett's multiple comparisons test**	**Mean Diff.**	**95.00% CI of diff.**	**Significant?**	**Summary**	**Adjusted P Value**	**A-?**	
CTR vs. 10nM 30m	-53.32	-106.1 to -0.5610	Yes	*	0.0463	B	10nM 30m
CTR vs. 10nM 3h	-110.1	-162.8 to -57.31	Yes	****	<0.0001	C	10nM 3h
CTR vs. 10nM 6h	-85.97	-138.7 to -33.21	Yes	***	0.0002	D	10nM 6h
CTR vs. 10nM 24h	-176.2	-229.0 to -123.4	Yes	****	<0.0001	E	10nM 24h
CTR vs. 50nM 30m	-85.32	-138.1 to -32.56	Yes	***	0.0002	F	50nM 30m
CTR vs. 50nM 3h	-147.8	-200.5 to -95.02	Yes	****	<0.0001	G	50nM 3h
CTR vs. 50nM 6h	-134.2	-187.0 to -81.43	Yes	****	<0.0001	H	50nM 6h
CTR vs. 50nM 24h	-204.1	-256.9 to -151.4	Yes	****	<0.0001	I	50nM 24h
CTR vs. 200nM 30m	-69.57	-122.3 to -16.81	Yes	**	0.0038	J	200nM 30m
CTR vs. 200nM 3h	-160.1	-212.8 to -107.3	Yes	****	<0.0001	K	200nM 3h
CTR vs. 200nM 6h	-160.6	-213.4 to -107.9	Yes	****	<0.0001	L	200nM 6h
CTR vs. 200nM 24h	-301.6	-354.3 to -248.8	Yes	****	<0.0001	M	200nM 24h

Test details	Mean 1	Mean 2	Mean Diff.	SE of diff.	n1	n2	q	DF
CTR vs. 10nM 30m	100.0	153.3	-53.32	18.52	6	6	2.879	65
CTR vs. 10nM 3h	100.0	210.1	-110.1	18.52	6	6	5.944	65
CTR vs. 10nM 6h	100.0	186.0	-85.97	18.52	6	6	4.642	65
CTR vs. 10nM 24h	100.0	276.2	-176.2	18.52	6	6	9.514	65
CTR vs. 50nM 30m	100.0	185.3	-85.32	18.52	6	6	4.607	65
CTR vs. 50nM 3h	100.0	247.8	-147.8	18.52	6	6	7.980	65
CTR vs. 50nM 6h	100.0	234.2	-134.2	18.52	6	6	7.246	65
CTR vs. 50nM 24h	100.0	304.1	-204.1	18.52	6	6	11.02	65
CTR vs. 200nM 30m	100.0	169.6	-69.57	18.52	6	6	3.757	65
CTR vs. 200nM 3h	100.0	260.1	-160.1	18.52	6	6	8.643	65
CTR vs. 200nM 6h	100.0	260.6	-160.6	18.52	6	6	8.675	65
CTR vs. 200nM 24h	100.0	401.6	-301.6	18.52	6	6	16.28	65

Statistics on mitochondrial ROS by MitoSOX

Normality and Lognormality Tests<br>Tabular results	A<br>CTR	B<br>10nM	C<br>50nM	D<br>200nM	E<br>FCCP
**Shapiro-Wilk test**					
W	0.9958	0.8339	0.9637	0.9719	0.9206
P value	0.9985	0.1159	0.8476	0.9052	0.5405
Passed normality test (alpha=0.05)?	Yes	Yes	Yes	Yes	Yes
P value summary	ns	ns	ns	ns	ns
**Kolmogorov-Smirnov test**					
KS distance	0.1239	0.3142	0.1567	0.1821	N too small
P value	>0.1000	0.0649	>0.1000	>0.1000	
Passed normality test (alpha=0.05)?	Yes	Yes	Yes	Yes	
P value summary	ns	ns	ns	ns	
**Number of values**	6	6	6	6	4

ANOVA results | Multiple comparisons

Ordinary one-way ANOVA<br>Multiple comparisons								
Number of families	1							
Number of comparisons per family	4							
Alpha	0.05							
**Dunnett's multiple comparisons test**	**Mean Diff.**	**95.00% CI of diff.**	**Significant?**	**Summary**	**Adjusted P Value**	**A-?**		
CTR vs. 10nM	-56.50	-111.3 to -1.722	Yes	*	0.0419	B	10nM	
CTR vs. 50nM	-107.3	-162.1 to -52.50	Yes	***	0.0001	C	50nM	
CTR vs. 200nM	-205.7	-260.5 to -151.0	Yes	****	<0.0001	D	200nM	
CTR vs. FCCP	-252.4	-313.6 to -191.1	Yes	****	<0.0001	E	FCCP	
**Test details**	**Mean 1**	**Mean 2**	**Mean Diff.**	**SE of diff.**	**n1**	**n2**	**q**	**DF**
CTR vs. 10nM	100.0	156.5	-56.50	20.83	6	6	2.712	23
CTR vs. 50nM	100.0	207.3	-107.3	20.83	6	6	5.150	23
CTR vs. 200nM	100.0	305.7	-205.7	20.83	6	6	9.876	23
CTR vs. FCCP	100.0	352.4	-252.4	23.29	6	4	10.83	23

Statistics on lipid peroxidation by Bodipy

Normality and Lognormality Tests<br>Tabular results	A<br>CTR	B<br>10nM 4h	C<br>50nM 4h	D<br>200nM 4h	E<br>800nM 4h	F<br>CTR24h	G<br>10nM 24h	H<br>50nM 24h	I<br>200nM 24h	J<br>800nM 24h
**Shapiro-Wilk test**										
W	0.9378	0.9639	0.9656	0.8963	0.9061	0.9743	0.9496	0.8635	0.8498	0.8984
P value	0.6413	0.8491	0.8616	0.3526	0.4115	0.9197	0.7373	0.2017	0.1568	0.3648
Passed normality test (alpha=0.05)?	Yes	Yes	Yes	Yes	Yes	Yes	Yes	Yes	Yes	Yes
P value summary	ns	ns	ns	ns	ns	ns	ns	ns	ns	ns
**Kolmogorov-Smirnov test**										
KS distance	0.1667	0.1955	0.2060	0.2173	0.2424	0.1667	0.1657	0.2267	0.2604	0.2237
P value	>0.1000	>0.1000	>0.1000	>0.1000	>0.1000	>0.1000	>0.1000	>0.1000	>0.1000	>0.1000
Passed normality test (alpha=0.05)?	Yes	Yes	Yes	Yes	Yes	Yes	Yes	Yes	Yes	Yes
P value summary	ns	ns	ns	ns	ns	ns	ns	ns	ns	ns
**Number of values**	6	6	6	6	6	6	6	6	6	6

Ordinary one-way ANOVA<br>Multiple comparisons								
Number of families	1							
Number of comparisons per family	9							
Alpha	0.05							
**Dunnett's multiple comparisons test**	**Mean Diff.**	**95.00% CI of diff.**	**Significant?**	**Summary**	**Adjusted P V**	**A-?**		
CTR vs. 10nM 4h	-34.67	-66.08 to -3.263	Yes	*	0.0240	B	10nM 4h	
CTR vs. 50nM 4h	-41.01	-72.41 to -9.598	Yes	**	0.0050	C	50nM 4h	
CTR vs. 200nM 4h	-57.69	-89.10 to -26.28	Yes	****	<0.0001	D	200nM 4h	
CTR vs. 800nM 4h	-60.45	-91.86 to -29.04	Yes	****	<0.0001	E	800nM 4h	
CTR vs. CTR24h	-9.000	-40.41 to 22.41	No	ns	0.9715	F	CTR24h	
CTR vs. 10nM 24h	-42.20	-73.60 to -10.79	Yes	**	0.0037	G	10nM 24h	
CTR vs. 50nM 24h	-61.56	-92.97 to -30.15	Yes	****	<0.0001	H	50nM 24h	
CTR vs. 200nM 24h	-64.79	-96.20 to -33.38	Yes	****	<0.0001	I	200nM 24h	
CTR vs. 800nM 24h	-74.81	-106.2 to -43.40	Yes	****	<0.0001	J	800nM 24h	
**Test details**	**Mean 1**	**Mean 2**	**Mean Diff.**	**SE of diff.**	**n1**	**n2**	**q**	**DF**
CTR vs. 10nM 4h	100.0	134.7	-34.67	11.27	6	6	3.076	50
CTR vs. 50nM 4h	100.0	141.0	-41.01	11.27	6	6	3.638	50
CTR vs. 200nM 4h	100.0	157.7	-57.69	11.27	6	6	5.117	50
CTR vs. 800nM 4h	100.0	160.5	-60.45	11.27	6	6	5.362	50
CTR vs. CTR24h	100.0	109.0	-9.000	11.27	6	6	0.7984	50
CTR vs. 10nM 24h	100.0	142.2	-42.20	11.27	6	6	3.743	50
CTR vs. 50nM 24h	100.0	161.6	-61.56	11.27	6	6	5.461	50
CTR vs. 200nM 24h	100.0	164.8	-64.79	11.27	6	6	5.747	50
CTR vs. 800nM 24h	100.0	174.8	-74.81	11.27	6	6	6.636	50

Statistics on MTR-stained mitochondria

Normality and Lognormality Tests<br>Tabular results	A<br>CTR	B<br>10nM	C<br>50nM	D<br>200nM
P value	0.7735	0.5498	0.5366	0.6055
Passed normality test (alpha=0.05)?	Yes	Yes	Yes	Yes
P value summary	ns	ns	ns	ns
**Kolmogorov-Smirnov test**				
KS distance	0.2107	0.1803	0.1671	0.1610
P value	>0.1000	>0.1000	>0.1000	>0.1000
Passed normality test (alpha=0.05)?	Yes	Yes	Yes	Yes
P value summary	ns	ns	ns	ns
**Number of values**	6	6	6	6

ANOVA results | Multiple comparisons

Ordinary one-way ANOVA<br>Multiple comparisons								
Number of families	1							
Number of comparisons per family	3							
Alpha	0.05							
**Dunnett's multiple comparisons test**	**Mean Diff.**	**95.00% CI of diff.**	**Significant?**	**Summary**	**Adjusted P Value**	**A-?**		
CTR vs. 10nM	19.50	7.364 to 31.64	Yes	**	0.0016	B	10nM	
CTR vs. 50nM	49.50	37.36 to 61.64	Yes	****	<0.0001	C	50nM	
CTR vs. 200nM	63.00	50.86 to 75.14	Yes	****	<0.0001	D	200nM	
**Test details**	**Mean 1**	**Mean 2**	**Mean Diff.**	**SE of diff.**	**n1**	**n2**	**q**	**DF**
CTR vs. 10nM	100.0	80.50	19.50	4.777	6	6	4.082	20
CTR vs. 50nM	100.0	50.50	49.50	4.777	6	6	10.36	20
CTR vs. 200nM	100.0	37.00	63.00	4.777	6	6	13.19	20

Statistics on TMRE-stained mitochondria

Normality and Lognormality Tests<br>Tabular results	A<br>CTR	B<br>Rot 10nM 4h	C<br>Rot 50nM 4h	D<br>Rot 200nM 4	E<br>Rot 800nM 4	F<br>CTR 24h	G<br>Rot 10nM 24	H<br>Rot 50nM 24	I<br>Rot 200nM 24	J<br>Rot 800nM 24	K<br>FCCP 1h
**Shapiro-Wilk test**											
W	0.9430	0.9240	0.9222	0.8725	0.9745	0.9178	0.8301	0.9050	0.8750	0.9196	0.9343
P value	0.6838	0.5348	0.5214	0.2364	0.9210	0.4896	0.1077	0.4046	0.2470	0.5022	0.6141
Passed normality test (alpha=0.05)?	Yes	Yes	Yes	Yes	Yes	Yes	Yes	Yes	Yes	Yes	Yes
P value summary	ns	ns	ns	ns	ns	ns	ns	ns	ns	ns	ns
**Kolmogorov-Smirnov test**											
KS distance	0.1965	0.1721	0.2459	0.2581	0.1362	0.2633	0.2701	0.2184	0.2310	0.2272	0.2286
P value	>0.1000	>0.1000	>0.1000	>0.1000	>0.1000	>0.1000	>0.1000	>0.1000	>0.1000	>0.1000	>0.1000
Passed normality test (alpha=0.05)?	Yes	Yes	Yes	Yes	Yes	Yes	Yes	Yes	Yes	Yes	Yes
P value summary	ns	ns	ns	ns	ns	ns	ns	ns	ns	ns	ns
**Number of values**	6	6	6	6	6	6	6	6	6	6	6

ANOVA results | Multiple comparisons

Ordinary one-way ANOVA
Multiple comparisons

1	Number of families	1						
2	Number of comparisons per family	10						
3	Alpha	0.05						
4								
5	**Dunnett's multiple comparisons tes**	**Mean Diff.**	**95.00% CI of diff.**	**Significant?**	**Summary**	**Adjusted P Value**	**A-?**	
6	CTR vs. Rot 10nM 4h	14.20	-3.804 to 32.20	No	ns	0.1881	B	Rot 10nM 4h
7	CTR vs. Rot 50nM 4h	23.37	5.363 to 41.37	Yes	**	0.0050	C	Rot 50nM 4h
8	CTR vs. Rot 200nM 4h	29.37	11.36 to 47.37	Yes	***	0.0003	D	Rot 200nM 4h
9	CTR vs. Rot 800nM 4h	33.87	15.86 to 51.87	Yes	****	<0.0001	E	Rot 800nM 4h
10	CTR vs. CTR 24h	5.700	-12.30 to 23.70	No	ns	0.9597	F	CTR 24h
11	CTR vs. Rot 10nM 24h	22.53	4.530 to 40.54	Yes	**	0.0074	G	Rot 10nM 24h
12	CTR vs. Rot 50nM 24h	34.20	16.20 to 52.20	Yes	****	<0.0001	H	Rot 50nM 24h
13	CTR vs. Rot 200nM 24h	37.53	19.53 to 55.54	Yes	****	<0.0001	I	Rot 200nM 24h
14	CTR vs. Rot 800nM 24h	54.70	36.70 to 72.70	Yes	****	<0.0001	J	Rot 800nM 24h
15	CTR vs. FCCP	41.58	23.58 to 59.58	Yes	****	<0.0001	K	FCCP

**Test details**	**Mean 1**	**Mean 2**	**Mean Diff.**	**SE of diff.**	**n1**	**n2**	**q**	**DF**
CTR vs. Rot 10nM 4h	100.0	85.83	14.20	6.409	6	6	2.216	55
CTR vs. Rot 50nM 4h	100.0	76.67	23.37	6.409	6	6	3.646	55
CTR vs. Rot 200nM 4h	100.0	70.67	29.37	6.409	6	6	4.582	55
CTR vs. Rot 800nM 4h	100.0	66.17	33.87	6.409	6	6	5.284	55
CTR vs. CTR 24h	100.0	94.33	5.700	6.409	6	6	0.8894	55
CTR vs. Rot 10nM 24h	100.0	77.50	22.53	6.409	6	6	3.516	55
CTR vs. Rot 50nM 24h	100.0	65.83	34.20	6.409	6	6	5.336	55
CTR vs. Rot 200nM 24h	100.0	62.50	37.53	6.409	6	6	5.856	55
CTR vs. Rot 800nM 24h	100.0	45.33	54.70	6.409	6	6	8.535	55
CTR vs. FCCP	100.0	58.45	41.58	6.409	6	6	6.488	55

Statistics on Total N° of Mitochondria - MTG stained mitochondria

Tabular results

Normality and Lognormality Tests<br>Tabular results	A<br>CTR	B<br>Rot 10nM 4	C<br>Rot 50nM 4	D<br>ot 200nM 4	E<br>ot 800nM 4	F<br>CTR 24h	G<br>ot 10nM 24	H<br>ot 50nM 24	I<br>ot 200nM 2	J<br>ot 800nM 2	K<br>FCCP
**Shapiro-Wilk test**											
W	0.9489	0.9017	0.9151	0.9842	0.9492	0.9097	0.9550	0.9694	0.8781	0.8784	0.9573
P value	0.7317	0.3843	0.4709	0.9706	0.7341	0.4344	0.7808	0.8885	0.2603	0.2616	0.7986
Passed normality test (alpha=0.05)?	Yes	Yes	Yes	Yes	Yes	Yes	Yes	Yes	Yes	Yes	Yes
P value summary	ns	ns	ns	ns	ns	ns	ns	ns	ns	ns	ns
**Kolmogorov-Smirnov test**											
KS distance	0.2176	0.2213	0.2265	0.1519	0.1991	0.1883	0.1612	0.1904	0.2670	0.2123	0.1738
P value	>0.1000	>0.1000	>0.1000	>0.1000	>0.1000	>0.1000	>0.1000	>0.1000	>0.1000	>0.1000	>0.1000
Passed normality test (alpha=0.05)?	Yes	Yes	Yes	Yes	Yes	Yes	Yes	Yes	Yes	Yes	Yes
P value summary	ns	ns	ns	ns	ns	ns	ns	ns	ns	ns	ns
**Number of values**	6	6	6	6	6	6	6	6	6	6	6

ANOVA results | Multiple comparisons

2way ANOVA
Multiple comparisons

Within each column, compare rows (simpl					
Number of families	2				
Number of comparisons per family	4				
Alpha	0.05				
**Dunnett's multiple comparisons test**	**Mean Diff.**	**95.00% CI of diff.**	**Significant?**	**Summary**	**Adjusted P Value**
4h					
CTR vs. 10nM	-16.55	-103.9 to 70.78	No	ns	0.9694
CTR vs. 50nM	-106.5	-193.8 to -19.17	Yes	*	0.0122
CTR vs. 200nM	-133.7	-221.0 to -46.34	Yes	**	0.0012
CTR vs. 800nM	-136.4	-223.8 to -49.11	Yes	***	0.0010
24h					
CTR vs. 10nM	-115.2	-202.5 to -27.84	Yes	**	0.0060
CTR vs. 50nM	-153.6	-241.0 to -66.31	Yes	***	0.0002
CTR vs. 200nM	-99.31	-186.6 to -11.99	Yes	*	0.0211
CTR vs. 800nM	-92.47	-179.8 to -5.140	Yes	*	0.0349

**Test details**	**Mean 1**	**Mean 2**	**Mean Diff.**	**SE of diff.**	**N1**	**N2**	**q**	**DF**
4h								
CTR vs. 10nM	100.0	116.6	-16.55	34.62	6	6	0.4780	50.00
CTR vs. 50nM	100.0	206.5	-106.5	34.62	6	6	3.076	50.00
CTR vs. 200nM	100.0	233.7	-133.7	34.62	6	6	3.861	50.00
CTR vs. 800nM	100.0	236.4	-136.4	34.62	6	6	3.941	50.00
24h								
CTR vs. 10nM	133.6	248.8	-115.2	34.62	6	6	3.327	50.00
CTR vs. 50nM	133.6	287.3	-153.6	34.62	6	6	4.438	50.00
CTR vs. 200nM	133.6	232.9	-99.31	34.62	6	6	2.868	50.00
CTR vs. 800nM	133.6	226.1	-92.47	34.62	6	6	2.671	50.00

Statistics on N° of Fragmented Mitochondria - MTG stained mitochondria

Tabular results

	Normality and Lognormality Tests<br>Tabular results	A<br>CTR	B<br>Rot 10nM 4	C<br>Rot 50nM 4	D<br>ot 200nM 4	E<br>ot 800nM 4	F<br>CTR 24h	G<br>ot 10nM 24	H<br>ot 50nM 24	I<br>ot 200nM 2	J<br>ot 800nM 2	K<br>FCCP
13												
14	**Shapiro-Wilk test**											
15	W	0.8741	0.8924	0.9685	0.9235	0.9145	0.9847	0.9446	0.9101	0.9027	0.9365	0.8801
16	P value	0.2431	0.3312	0.8825	0.5308	0.4665	0.9722	0.6961	0.4373	0.3900	0.6314	0.2697
17	Passed normality test (alpha=0.05)?	Yes	Yes	Yes	Yes	Yes	Yes	Yes	Yes	Yes	Yes	Yes
18	P value summary	ns	ns	ns	ns	ns	ns	ns	ns	ns	ns	ns
19												
20	**Kolmogorov-Smirnov test**											
21	KS distance	0.1981	0.2269	0.1783	0.1962	0.2028	0.1592	0.2159	0.2363	0.2244	0.1986	0.2855
22	P value	>0.1000	>0.1000	>0.1000	>0.1000	>0.1000	>0.1000	>0.1000	>0.1000	>0.1000	>0.1000	>0.1000
23	Passed normality test (alpha=0.05)?	Yes	Yes	Yes	Yes	Yes	Yes	Yes	Yes	Yes	Yes	Yes
24	P value summary	ns	ns	ns	ns	ns	ns	ns	ns	ns	ns	ns
25												
26	**Number of values**	6	6	6	6	6	6	6	6	6	6	6
27												

ANOVA results | Multiple comparisons

	2way ANOVA<br>Multiple comparisons					
1	Within each column, compare rows (simple effects within columns)					
2						
3	Number of families	2				
4	Number of comparisons per family	4				
5	Alpha	0.05				
6						
7	**Dunnett's multiple comparisons test**	**Mean Diff.**	**95.00% CI of diff.**	**Significant?**	**Summary**	**Adjusted P Valu**
8						
9	4h					
10	CTR vs. 10nM	-3.213	-26.28 to 19.85	No	ns	0.9900
11	CTR vs. 50nM	-38.65	-61.71 to -15.58	Yes	***	0.0004
12	CTR vs. 200nM	-51.28	-74.34 to -28.22	Yes	****	<0.0001
13	CTR vs. 800nM	-62.40	-85.46 to -39.33	Yes	****	<0.0001
14						
15	24h					
16	CTR vs. 10nM	-28.89	-51.95 to -5.822	Yes	**	0.0097
17	CTR vs. 50nM	-42.62	-65.68 to -19.56	Yes	****	<0.0001
18	CTR vs. 200nM	-46.67	-69.73 to -23.61	Yes	****	<0.0001
19	CTR vs. 800nM	-70.58	-93.64 to -47.51	Yes	****	<0.0001
20						

21									
22	**Test details**	**Mean 1**	**Mean 2**	**Mean Diff.**	**SE of diff.**	**N1**	**N2**	**q**	**DF**
23									
24	4h								
25	CTR vs. 10nM	100.0	103.2	-3.213	9.144	6	6	0.3514	50.00
26	CTR vs. 50nM	100.0	138.6	-38.65	9.144	6	6	4.227	50.00
27	CTR vs. 200nM	100.0	151.3	-51.28	9.144	6	6	5.608	50.00
28	CTR vs. 800nM	100.0	162.4	-62.40	9.144	6	6	6.824	50.00
29									
30	24h								
31	CTR vs. 10nM	102.2	131.1	-28.89	9.144	6	6	3.159	50.00
32	CTR vs. 50nM	102.2	144.8	-42.62	9.144	6	6	4.661	50.00
33	CTR vs. 200nM	102.2	148.9	-46.67	9.144	6	6	5.104	50.00
34	CTR vs. 800nM	102.2	172.8	-70.58	9.144	6	6	7.718	50.00
35									

Statistics on SUM Area of Fragmented Mitochondria - MTG stained mitochondria

Tabular results

	Normality and Lognormality Tests<br>Tabular results	A<br>CTR	B<br>Rot 10nM 4	C<br>Rot 50nM 4	D<br>ot 200nM 4	E<br>ot 800nM 4	F<br>CTR 24h	G<br>ot 10nM 24	H<br>ot 50nM 24	I<br>ot 200nM 2	J<br>ot 800nM 2	K<br>FCCP
13												
14	**Shapiro-Wilk test**											
15	W	0.9762	0.9436	0.8382	0.9526	0.9209	0.9133	0.9580	0.9749	0.9132	0.8880	0.8757
16	P value	0.9311	0.6880	0.1260	0.7613	0.5115	0.4586	0.8045	0.9234	0.4577	0.3079	0.2498
17	Passed normality test (alpha=0.05)?	Yes	Yes	Yes	Yes	Yes	Yes	Yes	Yes	Yes	Yes	Yes
18	P value summary	ns	ns	ns	ns	ns	ns	ns	ns	ns	ns	ns
19												
20	**Kolmogorov-Smirnov test**											
21	KS distance	0.1739	0.2044	0.3064	0.1521	0.2216	0.2448	0.2001	0.1566	0.2532	0.2074	0.2644
22	P value	>0.1000	>0.1000	0.0817	>0.1000	>0.1000	>0.1000	>0.1000	>0.1000	>0.1000	>0.1000	>0.1000
23	Passed normality test (alpha=0.05)?	Yes	Yes	Yes	Yes	Yes	Yes	Yes	Yes	Yes	Yes	Yes
24	P value summary	ns	ns	ns	ns	ns	ns	ns	ns	ns	ns	ns
25												
26	**Number of values**	6	6	6	6	6	6	6	6	6	6	6
27												

ANVOA results | Multiple comparisons

	2way ANOVA<br>Multiple comparisons								
1	Within each column, compare rows (simple effects within columns)								
2									
3	Number of families	2							
4	Number of comparisons per family	4							
5	Alpha	0.05							
6									
7	**Dunnett's multiple comparisons test**	**Mean Diff.**	**95.00% CI of diff.**	**Significant?**	**Summary**	**Adjusted P Value**			
8									
9	4h								
10	CTR vs. 10nM	-40.73	-73.08 to -8.373	Yes	**	0.0093			
11	CTR vs. 50nM	-90.34	-122.7 to -57.99	Yes	****	<0.0001			
12	CTR vs. 200nM	-226.7	-259.1 to -194.4	Yes	****	<0.0001			
13	CTR vs. 800nM	-243.8	-276.1 to -211.4	Yes	****	<0.0001			
14									
15	24h								
16	CTR vs. 10nM	-82.17	-114.5 to -49.82	Yes	****	<0.0001			
17	CTR vs. 50nM	-195.5	-227.9 to -163.2	Yes	****	<0.0001			
18	CTR vs. 200nM	-221.9	-254.3 to -189.6	Yes	****	<0.0001			
19	CTR vs. 800nM	-252.6	-285.0 to -220.3	Yes	****	<0.0001			
20									
21									
22	**Test details**	**Mean 1**	**Mean 2**	**Mean Diff.**	**SE of diff.**	**N1**	**N2**	**q**	**DF**
23									
24	4h								
25	CTR vs. 10nM	100.0	140.7	-40.73	12.83	6	6	3.175	50.00
26	CTR vs. 50nM	100.0	190.3	-90.34	12.83	6	6	7.043	50.00
27	CTR vs. 200nM	100.0	326.7	-226.7	12.83	6	6	17.67	50.00
28	CTR vs. 800nM	100.0	343.8	-243.8	12.83	6	6	19.00	50.00
29									
30	24h								
31	CTR vs. 10nM	124.7	206.9	-82.17	12.83	6	6	6.406	50.00
32	CTR vs. 50nM	124.7	320.2	-195.5	12.83	6	6	15.24	50.00
33	CTR vs. 200nM	124.7	346.6	-221.9	12.83	6	6	17.30	50.00
34	CTR vs. 800nM	124.7	377.3	-252.6	12.83	6	6	19.70	50.00
35									
36									

Statistics on the Ratio SUM Area/N° Fragmented Mitochondria - MTG stained mitochondria

Tabular results

Normality and Lognormality Tests<br>Tabular results	A<br>CTR	B<br>Rot 10nM 4	C<br>Rot 50nM 4	D<br>ot 200nM 4	E<br>ot 800nM 4	F<br>CTR 24h	G<br>ot 10nM 24	H<br>ot 50nM 24	I<br>ot 200nM 2	J<br>ot 800nM 2	K<br>FCCP
13											
14 **Shapiro-Wilk test**											
15 W	0.9087	0.8461	0.9810	0.9486	0.9273	0.9294	0.9403	0.9536	0.9581	0.9370	0.9039
16 P value	0.4280	0.1464	0.9566	0.7293	0.5592	0.5754	0.6618	0.7697	0.8046	0.6349	0.3974
17 Passed normality test (alpha=0.05)?	Yes	Yes	Yes	Yes	Yes	Yes	Yes	Yes	Yes	Yes	Yes
18 P value summary	ns	ns	ns	ns	ns	ns	ns	ns	ns	ns	ns
19											
20 **Kolmogorov-Smirnov test**											
21 KS distance	0.2674	0.2317	0.1798	0.2033	0.2794	0.1840	0.1913	0.1557	0.2241	0.1848	0.2051
22 P value	>0.1000	>0.1000	>0.1000	>0.1000	>0.1000	>0.1000	>0.1000	>0.1000	>0.1000	>0.1000	>0.1000
23 Passed normality test (alpha=0.05)?	Yes	Yes	Yes	Yes	Yes	Yes	Yes	Yes	Yes	Yes	Yes
24 P value summary	ns	ns	ns	ns	ns	ns	ns	ns	ns	ns	ns
25											
26 **Number of values**	6	6	6	6	6	6	6	6	6	6	6
27											

ANOVA results | Multiple comparisons

2way ANOVA<br>Multiple comparisons					
1 Within each column, compare rows (simple effects within columns)					
2					
3 Number of families	2				
4 Number of comparisons per family	4				
5 Alpha	0.05				
6					
7 **Dunnett's multiple comparisons test**	**Mean Diff.**	**95.00% CI of diff.**	**Significant?**	**Summary**	**Adjusted P Value**
8					
9 4h					
10 CTR vs. 10nM	-35.36	-65.69 to -5.032	Yes	*	0.0175
11 CTR vs. 50nM	-37.08	-67.41 to -6.747	Yes	*	0.0119
12 CTR vs. 200nM	-113.8	-144.1 to -83.48	Yes	****	<0.0001
13 CTR vs. 800nM	-110.6	-140.9 to -80.26	Yes	****	<0.0001
14					
15 24h					
16 CTR vs. 10nM	-33.36	-63.69 to -3.025	Yes	*	0.0270
17 CTR vs. 50nM	-95.63	-126.0 to -65.30	Yes	****	<0.0001
18 CTR vs. 200nM	-106.6	-136.9 to -76.28	Yes	****	<0.0001
19 CTR vs. 800nM	-92.39	-122.7 to -62.06	Yes	****	<0.0001
20					

21								
22 **Test details**	**Mean 1**	**Mean 2**	**Mean Diff.**	**SE of diff.**	**N1**	**N2**	**q**	**DF**
23								
24 4h								
25 CTR vs. 10nM	100.0	135.4	-35.36	12.03	6	6	2.941	50.00
26 CTR vs. 50nM	100.0	137.1	-37.08	12.03	6	6	3.083	50.00
27 CTR vs. 200nM	100.0	213.8	-113.8	12.03	6	6	9.464	50.00
28 CTR vs. 800nM	100.0	210.6	-110.6	12.03	6	6	9.196	50.00
29								
30 24h								
31 CTR vs. 10nM	124.9	158.2	-33.36	12.03	6	6	2.774	50.00
32 CTR vs. 50nM	124.9	220.5	-95.63	12.03	6	6	7.953	50.00
33 CTR vs. 200nM	124.9	231.5	-106.6	12.03	6	6	8.866	50.00
34 CTR vs. 800nM	124.9	217.3	-92.39	12.03	6	6	7.683	50.00
35								

Statistics on N° of Synaptic Mitochondria - MTG stained mitochondria

Tabular results

Normality and Lognormality Tests
Tabular results

		A	B	C	D	E	F	G	H	I
		CTR	10nM 4h	50nM 4h	200nM 4h	800nM 4h	10nM 24h	50nM 24h	200nM 24h	800nM 24h
13										
14	**Shapiro-Wilk test**									
15	W	0.8750	0.9199	0.8986	0.8064	0.9542	0.9159	0.9145	0.8637	0.9173
16	P value	0.2874	0.5291	0.4023	0.0913	0.7669	0.5039	0.4952	0.2417	0.5128
17	Passed normality test (alpha=0.05)?	Yes	Yes	Yes	Yes	Yes	Yes	Yes	Yes	Yes
18	P value summary	ns	ns	ns	ns	ns	ns	ns	ns	ns
19										
20	**Kolmogorov-Smirnov test**									
21	KS distance	0.2874	0.1950	0.2187	0.3316	0.2225	0.2139	0.2046	0.2427	0.2478
22	P value	>0.1000	>0.1000	>0.1000	0.0758	>0.1000	>0.1000	>0.1000	>0.1000	>0.1000
23	Passed normality test (alpha=0.05)?	Yes	Yes	Yes	Yes	Yes	Yes	Yes	Yes	Yes
24	P value summary	ns	ns	ns	ns	ns	ns	ns	ns	ns
25										
26	**Number of values**	5	5	5	5	5	5	5	5	5
27										

ANOVA results | Multiple comparisons

2way ANOVA
Multiple comparisons

1	Within each column, compare rows (simple effects within colu								
2									
3	Number of families	2							
4	Number of comparisons per family	4							
5	Alpha	0.05							
6									
7	**Dunnett's multiple comparisons test**	**Mean Diff.**	**95.00% CI of dif**	**Significant?**	**Summary**	**Adjusted P Valu**			
8									
9	4h								
10	CTR vs. 10nM	-10.41	-23.65 to 2.823	No	ns	0.1594			
11	CTR vs. 50nM	7.648	-5.589 to 20.89	No	ns	0.3987			
12	CTR vs. 200nM	37.09	23.85 to 50.32	Yes	****	<0.0001			
13	CTR vs. 800nM	52.02	38.78 to 65.26	Yes	****	<0.0001			
14									
15	24h								
16	CTR vs. 10nM	4.752	-8.485 to 17.99	No	ns	0.7707			
17	CTR vs. 50nM	5.410	-7.827 to 18.65	No	ns	0.6859			
18	CTR vs. 200nM	37.20	23.96 to 50.44	Yes	****	<0.0001			
19	CTR vs. 800nM	50.85	37.62 to 64.09	Yes	****	<0.0001			
20									
21									
22	**Test details**	**Mean 1**	**Mean 2**	**Mean Diff.**	**SE of diff.**	**N1**	**N2**	**q**	**DF**
23									
24	4h								
25	CTR vs. 10nM	100.0	110.4	-10.41	5.205	5	5	2.001	40.00
26	CTR vs. 50nM	100.0	92.35	7.648	5.205	5	5	1.469	40.00
27	CTR vs. 200nM	100.0	62.91	37.09	5.205	5	5	7.125	40.00
28	CTR vs. 800nM	100.0	47.98	52.02	5.205	5	5	9.993	40.00
29									
30	24h								
31	CTR vs. 10nM	107.2	102.4	4.752	5.205	5	5	0.9129	40.00
32	CTR vs. 50nM	107.2	101.8	5.410	5.205	5	5	1.039	40.00
33	CTR vs. 200nM	107.2	69.96	37.20	5.205	5	5	7.147	40.00
34	CTR vs. 800nM	107.2	56.31	50.85	5.205	5	5	9.770	40.00
35									

Statistics on Mean Synaptic Mitochondria Area - MTG stained mitochondria

Tabular results

Normality and Lognormality Tests<br>Tabular results	A<br>CTR 24h	B<br>10nM 4h	C<br>50nM 4h	D<br>200nM 4h	E<br>800nM 4h	F<br>10nM 24h	G<br>50nM 24h	H<br>200nM 24h	I<br>800nM 24h
13									
14 **Shapiro-Wilk test**									
15 W	0.8565	0.8190	0.9294	0.9245	0.9207	0.9454	0.8310	0.9278	0.9581
16 P value	0.2160	0.1146	0.5921	0.5594	0.5343	0.7045	0.1416	0.5815	0.7945
17 Passed normality test (alpha=0.05)?	Yes	Yes	Yes	Yes	Yes	Yes	Yes	Yes	Yes
18 P value summary	ns	ns	ns	ns	ns	ns	ns	ns	ns
19									
20 **Kolmogorov-Smirnov test**									
21 KS distance	0.2159	0.2992	0.1954	0.2548	0.2021	0.2006	0.3002	0.2431	0.2392
22 P value	>0.1000	>0.1000	>0.1000	>0.1000	>0.1000	>0.1000	>0.1000	>0.1000	>0.1000
23 Passed normality test (alpha=0.05)?	Yes	Yes	Yes	Yes	Yes	Yes	Yes	Yes	Yes
24 P value summary	ns	ns	ns	ns	ns	ns	ns	ns	ns
25									
26 **Number of values**	5	5	5	5	5	5	5	5	5
27									

ANOVA results | Multiple comparisons

2way ANOVA<br>Multiple comparisons								
1 Within each column, compare rows (simple effects within columr								
2								
3 Number of families	2							
4 Number of comparisons per family	4							
5 Alpha	0.05							
6								
7 **Dunnett's multiple comparisons test**	**Mean Diff**	**95.00% CI of dif**	**Significan**	**Summary**	**Adjusted P '**			
8								
9 4h								
10 CTR vs. 10nM	7.408	-7.136 to 21.95	No	ns	0.5090			
11 CTR vs. 50nM	0.9420	-13.60 to 15.49	No	ns	0.9993			
12 CTR vs. 200nM	-0.6740	-15.22 to 13.87	No	ns	0.9998			
13 CTR vs. 800nM	-23.15	-37.70 to -8.608	Yes	***	0.0009			
14								
15 24h								
16 CTR vs. 10nM	11.57	-2.974 to 26.11	No	ns	0.1525			
17 CTR vs. 50nM	3.488	-11.06 to 18.03	No	ns	0.9298			
18 CTR vs. 200nM	-10.37	-24.92 to 4.172	No	ns	0.2261			
19 CTR vs. 800nM	-27.53	-42.07 to -12.98	Yes	****	<0.0001			
20								
21								
22 **Test details**	**Mean 1**	**Mean 2**	**Mean Diff.**	**SE of diff.**	**N1**	**N2**	**q**	**DF**
23								
24 4h								
25 CTR vs. 10nM	100.0	92.59	7.408	5.719	5	5	1.295	40.00
26 CTR vs. 50nM	100.0	99.06	0.9420	5.719	5	5	0.1647	40.00
27 CTR vs. 200nM	100.0	100.7	-0.6740	5.719	5	5	0.1179	40.00
28 CTR vs. 800nM	100.0	123.2	-23.15	5.719	5	5	4.048	40.00
29								
30 24h								
31 CTR vs. 10nM	108.2	96.63	11.57	5.719	5	5	2.023	40.00
32 CTR vs. 50nM	108.2	104.7	3.488	5.719	5	5	0.6099	40.00
33 CTR vs. 200nM	108.2	118.6	-10.37	5.719	5	5	1.814	40.00
34 CTR vs. 800nM	108.2	135.7	-27.53	5.719	5	5	4.813	40.00
35								

Statistics on Mitochondria Proportional Area - MTG stained mitochondria

Tabular results

Normality and Lognormality Tests<br>Tabular results	A<br>CTR 24h	B<br>10nM 4h	C<br>50nM 4h	D<br>200nM 4h	E<br>800nM 4h	F<br>10nM 24h	G<br>50nM 24h	H<br>200nM 24h	I<br>800nM 24h
**Shapiro-Wilk test**									
W	0.9423	0.9057	0.9702	0.9374	0.9167	0.9056	0.9779	0.9500	0.8890
P value	0.6824	0.4420	0.8765	0.6478	0.5090	0.4417	0.9234	0.7372	0.3521
Passed normality test (alpha=0.05)?	Yes	Yes	Yes	Yes	Yes	Yes	Yes	Yes	Yes
P value summary	ns	ns	ns	ns	ns	ns	ns	ns	ns
**Kolmogorov-Smirnov test**									
KS distance	0.2579	0.2761	0.1858	0.2527	0.2578	0.2329	0.1786	0.2175	0.3228
P value	>0.1000	>0.1000	>0.1000	>0.1000	>0.1000	>0.1000	>0.1000	>0.1000	0.0965
Passed normality test (alpha=0.05)?	Yes	Yes	Yes	Yes	Yes	Yes	Yes	Yes	Yes
P value summary	ns	ns	ns	ns	ns	ns	ns	ns	ns
**Number of values**	5	5	5	5	5	5	5	5	5

ANOVA results | Multiple comparisons

2way ANOVA
Multiple comparisons

Within each column, compare rows (simple effects within cc					
Number of families	2				
Number of comparisons per family	4				
Alpha	0.05				
**Dunnett's multiple comparisons test**	**Mean Diff.**	**95.00% CI of diff.**	**Significant'**	**Summary**	**Adjusted P Value**
4h					
CTR vs. 10nM	-18.87	-35.09 to -2.639	Yes	*	0.0182
CTR vs. 50nM	6.974	-9.253 to 23.20	No	ns	0.6485
CTR vs. 200nM	37.19	20.96 to 53.41	Yes	****	<0.0001
CTR vs. 800nM	59.82	43.59 to 76.04	Yes	****	<0.0001
24h					
CTR vs. 10nM	1.858	-14.37 to 18.08	No	ns	0.9951
CTR vs. 50nM	8.680	-7.547 to 24.91	No	ns	0.4663
CTR vs. 200nM	48.80	32.57 to 65.02	Yes	****	<0.0001
CTR vs. 800nM	65.99	49.76 to 82.21	Yes	****	<0.0001

**Test details**	**Mean 1**	**Mean 2**	**Mean Diff.**	**SE of diff.**	**N1**	**N2**	**q**	**DF**
4h								
CTR vs. 10nM	100.2	119.1	-18.87	6.381	5	5	2.957	40.00
CTR vs. 50nM	100.2	93.25	6.974	6.381	5	5	1.093	40.00
CTR vs. 200nM	100.2	63.04	37.19	6.381	5	5	5.828	40.00
CTR vs. 800nM	100.2	40.41	59.82	6.381	5	5	9.375	40.00
24h								
CTR vs. 10nM	107.8	105.9	1.858	6.381	5	5	0.291	40.00
CTR vs. 50nM	107.8	99.12	8.680	6.381	5	5	1.360	40.00
CTR vs. 200nM	107.8	59.00	48.80	6.381	5	5	7.648	40.00
CTR vs. 800nM	107.8	41.81	65.99	6.381	5	5	10.34	40.00

Statistics on bioenergetics – Basal respiration

Normality and Lognormality Tests<br>Tabular results	A<br>CTR	B<br>10nM 3h	C<br>10nM 6h	D<br>10nM 24h	E<br>50nM 3h
**Shapiro-Wilk test**					
W	0.9588	0.9514	0.9249	0.8336	0.8876
P value	0.7984	0.7519	0.4710	0.0647	0.3057
Passed normality test (alpha=0.05)?	Yes	Yes	Yes	Yes	Yes
P value summary	ns	ns	ns	ns	ns
**Kolmogorov-Smirnov test**					
KS distance	0.1622	0.2327	0.1627	0.2507	0.2777
P value	>0.1000	>0.1000	>0.1000	>0.1000	>0.1000
Passed normality test (alpha=0.05)?	Yes	Yes	Yes	Yes	Yes
P value summary	ns	ns	ns	ns	ns
**Number of values**	8	6	8	8	6

Ordinary one-way ANOVA<br>Multiple comparisons								
Number of families	1							
Number of comparisons per family	4							
Alpha	0.05							
**Dunnett's multiple comparisons test**	**Mean Diff.**	**95.00% CI of diff.**	**Significant?**	**Summary**	**Adjusted P Value**	**A-?**		
CTR vs. 10nM 3h	29.26	5.663 to 52.86	Yes	*	0.0113	B	10nM 3h	
CTR vs. 10nM 6h	34.80	12.95 to 56.65	Yes	**	0.0010	C	10nM 6h	
CTR vs. 10nM 24h	62.48	40.63 to 84.33	Yes	****	<0.0001	D	10nM 24h	
CTR vs. 50nM 3h	78.78	55.18 to 102.4	Yes	****	<0.0001	E	50nM 3h	
**Test details**	**Mean 1**	**Mean 2**	**Mean Diff.**	**SE of diff.**	**n1**	**n2**	**q**	**DF**
CTR vs. 10nM 3h	85.67	56.41	29.26	9.136	8	6	3.203	31
CTR vs. 10nM 6h	85.67	50.88	34.80	8.458	8	8	4.114	31
CTR vs. 10nM 24h	85.67	23.19	62.48	8.458	8	8	7.387	31
CTR vs. 50nM 3h	85.67	6.895	78.78	9.136	8	6	8.623	31

Statistics on bioenergetics – Maximal respiration

Normality and Lognormality Tests<br>Tabular results	A<br>CTR	B<br>Rot10nM 3h	C<br>Rot10nM 6h	D<br>Rot10nM 24h	E<br>Rot50nM 24h
**Shapiro-Wilk test**					
W	0.8643	0.9230	0.9517	0.8244	0.9784
P value	0.1324	0.5270	0.7279	0.0519	0.9436
Passed normality test (alpha=0.05)?	Yes	Yes	Yes	Yes	Yes
P value summary	ns	ns	ns	ns	ns
**Kolmogorov-Smirnov test**					
KS distance	0.2155	0.2766	0.1648	0.2344	0.1473
P value	>0.1000	>0.1000	>0.1000	>0.1000	>0.1000
Passed normality test (alpha=0.05)?	Yes	Yes	Yes	Yes	Yes
P value summary	ns	ns	ns	ns	ns
**Number of values**	8	6	8	8	6

Ordinary one-way ANOVA<br>Multiple comparisons								
Number of families	1							
Number of comparisons per family	4							
Alpha	0.05							
**Dunnett's multiple comparisons test**	**Mean Diff.**	**95.00% CI of diff.**	**Significant?**	**Summary**	**Adjusted P Value**	**A-?**		
CTR vs. Rot10nM 3h	14.53	2.774 to 26.29	Yes	*	0.0116	B	Rot10nM 3h	
CTR vs. Rot10nM 6h	17.74	6.851 to 28.62	Yes	***	0.0008	C	Rot10nM 6h	
CTR vs. Rot10nM 24h	44.21	33.32 to 55.09	Yes	****	<0.0001	D	Rot10nM 24h	
CTR vs. Rot50nM 24h	55.51	43.75 to 67.27	Yes	****	<0.0001	E	Rot50nM 24h	
**Test details**	**Mean 1**	**Mean 2**	**Mean Diff.**	**SE of diff.**	**n1**	**n2**	**q**	**DF**
CTR vs. Rot10nM 3h	61.76	47.23	14.53	4.552	8	6	3.193	31
CTR vs. Rot10nM 6h	61.76	44.03	17.74	4.214	8	8	4.209	31
CTR vs. Rot10nM 24h	61.76	17.55	44.21	4.214	8	8	10.49	31
CTR vs. Rot50nM 24h	61.76	6.252	55.51	4.552	8	6	12.20	31

Statistics on bioenergetics – ATP-linked respiration

Normality and Lognormality Tests<br>Tabular results	A<br>CTR	B<br>Rot10nM 3h	C<br>Rot10nM 6h	D<br>Rot10nM 24h	E<br>Rot50nM 24h
**Shapiro-Wilk test**					
W	0.9029	0.9154	0.9519	0.8700	0.8314
P value	0.3068	0.4731	0.7470	0.1506	0.1104
Passed normality test (alpha=0.05)?	Yes	Yes	Yes	Yes	Yes
P value summary	ns	ns	ns	ns	ns
**Kolmogorov-Smirnov test**					
KS distance	0.2096	0.2006	0.1654	0.2591	0.2993
P value	>0.1000	>0.1000	>0.1000	>0.1000	0.1000
Passed normality test (alpha=0.05)?	Yes	Yes	Yes	Yes	Yes
P value summary	ns	ns	ns	ns	ns
**Number of values**	8	6	7	8	6

Ordinary one-way ANOVA<br>Multiple comparisons								
Number of families	1							
Number of comparisons per family	4							
Alpha	0.05							
**Dunnett's multiple comparisons test**	**Mean Diff.**	**95.00% CI of diff.**	**Significant?**	**Summary**	**Adjusted P Value**	**A-?**		
CTR vs. Rot10nM 3h	16.95	2.834 to 31.06	Yes	*	0.0146	B	Rot10nM 3h	
CTR vs. Rot10nM 6h	20.69	7.161 to 34.21	Yes	**	0.0016	C	Rot10nM 6h	
CTR vs. Rot10nM 24h	30.84	17.78 to 43.91	Yes	****	<0.0001	D	Rot10nM 24h	
CTR vs. Rot50nM 24h	37.82	23.71 to 51.94	Yes	****	<0.0001	E	Rot50nM 24h	
**Test details**	**Mean 1**	**Mean 2**	**Mean Diff.**	**SE of diff.**	**n1**	**n2**	**q**	**DF**
CTR vs. Rot10nM 3h	44.15	27.21	16.95	5.450	8	6	3.110	30
CTR vs. Rot10nM 6h	44.15	23.47	20.69	5.223	8	7	3.961	30
CTR vs. Rot10nM 24h	44.15	13.31	30.84	5.046	8	8	6.113	30
CTR vs. Rot50nM 24h	44.15	6.331	37.82	5.450	8	6	6.940	30

Statistics on bioenergetics – Coupling efficiency

Normality and Lognormality Tests<br>Tabular results	A<br>CTR	B<br>Rot10nM 3h	C<br>Rot10nM 6h	D<br>Rot10nM 24h	E<br>Rot50nM 24h
**Shapiro-Wilk test**					
W	0.9259	0.8881	0.8559	0.9138	0.9391
P value	0.5486	0.3081	0.1757	0.4619	0.6521
Passed normality test (alpha=0.05)?	Yes	Yes	Yes	Yes	Yes
P value summary	ns	ns	ns	ns	ns
**Kolmogorov-Smirnov test**					
KS distance	0.2498	0.2029	0.2388	0.2011	0.1908
P value	>0.1000	>0.1000	>0.1000	>0.1000	>0.1000
Passed normality test (alpha=0.05)?	Yes	Yes	Yes	Yes	Yes
P value summary	ns	ns	ns	ns	ns
**Number of values**	6	6	6	6	6

Ordinary one-way ANOVA<br>Multiple comparisons								
Number of families	1							
Number of comparisons per family	4							
Alpha	0.05							
**Dunnett's multiple comparisons test**	**Mean Diff.**	**95.00% CI of diff.**	**Significant?**	**Summary**	**Adjusted P Value**	**A-?**		
CTR vs. Rot10nM 3h	6.292	-20.30 to 32.88	No	ns	0.9264	B	Rot10nM 3h	
CTR vs. Rot10nM 6h	6.633	-19.96 to 33.22	No	ns	0.9130	C	Rot10nM 6h	
CTR vs. Rot10nM 24h	39.20	12.61 to 65.79	Yes	**	0.0027	D	Rot10nM 24h	
CTR vs. Rot50nM 24h	33.41	6.815 to 60.00	Yes	*	0.0109	E	Rot50nM 24h	
**Test details**	**Mean 1**	**Mean 2**	**Mean Diff.**	**SE of diff.**	**n1**	**n2**	**q**	**DF**
CTR vs. Rot10nM 3h	61.54	55.25	6.292	10.20	6	6	0.6169	25
CTR vs. Rot10nM 6h	61.54	54.91	6.633	10.20	6	6	0.6503	25
CTR vs. Rot10nM 24h	61.54	22.34	39.20	10.20	6	6	3.843	25
CTR vs. Rot50nM 24h	61.54	28.14	33.41	10.20	6	6	3.275	25

Statistics on bioenergetics – Spared respiratory capacity

Normality and Lognormality Tests<br>Tabular results	A<br>CTR	B<br>Rot10nM 3h	C<br>Rot10nM 6h	D<br>Rot10nM 24h	E<br>Rot50nM 24h
**Shapiro-Wilk test**					
W	0.8424	0.9445	0.9539	0.9153	0.9240
P value	0.1365	0.6960	0.7720	0.4722	0.5347
Passed normality test (alpha=0.05)?	Yes	Yes	Yes	Yes	Yes
P value summary	ns	ns	ns	ns	ns
**Kolmogorov-Smirnov test**					
KS distance	0.2580	0.2119	0.1738	0.1982	0.1929
P value	>0.1000	>0.1000	>0.1000	>0.1000	>0.1000
Passed normality test (alpha=0.05)?	Yes	Yes	Yes	Yes	Yes
P value summary	ns	ns	ns	ns	ns
**Number of values**	6	6	6	6	6

Ordinary one-way ANOVA<br>Multiple comparisons							
Number of families	1						
Number of comparisons per family	4						
Alpha	0.05						
**Dunnett's multiple comparisons test**	**Mean Diff.**	**95.00% CI of diff.**	**Significant?**	**Summary**	**Adjusted P Value**	**A-?**	
CTR vs. Rot10nM 3h	35.69	11.73 to 59.65	Yes	**	0.0024	B	Rot10nM 3h
CTR vs. Rot10nM 6h	46.65	22.69 to 70.60	Yes	***	0.0001	C	Rot10nM 6h
CTR vs. Rot10nM 24h	43.50	19.54 to 67.46	Yes	***	0.0003	D	Rot10nM 24h
CTR vs. Rot50nM 24h	45.78	21.82 to 69.74	Yes	***	0.0002	E	Rot50nM 24h
**Test details**	**Mean 1**	**Mean 2**	**Mean Diff.**	**SE of diff.**	**n1**	**n2**	**q**	**DF**
CTR vs. Rot10nM 3h	89.73	54.04	35.69	9.190	6	6	3.883	25
CTR vs. Rot10nM 6h	89.73	43.08	46.65	9.190	6	6	5.076	25
CTR vs. Rot10nM 24h	89.73	46.23	43.50	9.190	6	6	4.734	25
CTR vs. Rot50nM 24h	89.73	43.95	45.78	9.190	6	6	4.981	25

Statistics on bioenergetics – Proton Leak respiration

Normality and Lognormality Tests<br>Tabular results	A<br>CTR	B<br>Rot10nM 3h	C<br>Rot10nM 6h	D<br>Rot10nM 24h	E<br>Rot50nM 24h
**Shapiro-Wilk test**					
W	0.8427	0.8414	0.8512	0.8696	0.8699
P value	0.0802	0.1340	0.0981	0.1494	0.2257
Passed normality test (alpha=0.05)?	Yes	Yes	Yes	Yes	Yes
P value summary	ns	ns	ns	ns	ns
**Kolmogorov-Smirnov test**					
KS distance	0.2805	0.2649	0.2263	0.2154	0.2660
P value	0.0634	>0.1000	>0.1000	>0.1000	>0.1000
Passed normality test (alpha=0.05)?	Yes	Yes	Yes	Yes	Yes
P value summary	ns	ns	ns	ns	ns
**Number of values**	8	6	8	8	6

Ordinary one-way ANOVA<br>Multiple comparisons								
Number of families	1							
Number of comparisons per family	4							
Alpha	0.05							
**Dunnett's multiple comparisons test**	**Mean Diff.**	**95.00% CI of diff.**	**Significant?**	**Summary**	**Adjusted P Value**	**A-?**		
CTR vs. Rot10nM 3h	9.494	-8.704 to 27.69	No	ns	0.4873	B	Rot10nM 3h	
CTR vs. Rot10nM 6h	11.18	-5.665 to 28.03	No	ns	0.2800	C	Rot10nM 6h	
CTR vs. Rot10nM 24h	21.10	4.254 to 37.95	Yes	*	0.0104	D	Rot10nM 24h	
CTR vs. Rot50nM 24h	28.79	10.59 to 46.99	Yes	**	0.0011	E	Rot50nM 24h	
**Test details**	**Mean 1**	**Mean 2**	**Mean Diff.**	**SE of diff.**	**n1**	**n2**	**q**	**DF**
CTR vs. Rot10nM 3h	42.72	33.22	9.494	7.045	8	6	1.348	31
CTR vs. Rot10nM 6h	42.72	31.53	11.18	6.523	8	8	1.715	31
CTR vs. Rot10nM 24h	42.72	21.61	21.10	6.523	8	8	3.235	31
CTR vs. Rot50nM 24h	42.72	13.93	28.79	7.045	8	6	4.086	31

Statistics on bioenergetics – non-mitochondrial respiration

Normality and Lognormality Tests<br>Tabular results	A<br>CTR	B<br>Rot10nM 3h	C<br>Rot10nM 6h	D<br>Rot10nM 24h	E<br>Rot50nM 24h
**Shapiro-Wilk test**					
W	0.9348	0.9625	0.7430	0.7341	0.9416
P value	0.6174	0.8391	0.0170	0.0139	0.6722
Passed normality test (alpha=0.05)?	Yes	Yes	No	No	Yes
P value summary	ns	ns	*	*	ns
**Kolmogorov-Smirnov test**					
KS distance	0.1975	0.1466	0.2777	0.3427	0.1903
P value	>0.1000	>0.1000	>0.1000	0.0264	>0.1000
Passed normality test (alpha=0.05)?	Yes	Yes	Yes	No	Yes
P value summary	ns	ns	ns	*	ns
**Number of values**	6	6	6	6	6

Ordinary one-way ANOVA<br>Multiple comparisons							
Number of families	1						
Number of comparisons per family	4						
Alpha	0.05						
**Dunnett's multiple comparisons test**	**Mean Diff.**	**95.00% CI of diff.**	**Significant?**	**Summary**	**Adjusted P Value**	**A-?**	
CTR vs. Rot10nM 3h	2.426	-5.880 to 10.73	No	ns	0.8602	B	Rot10nM 3h
CTR vs. Rot10nM 6h	0.8137	-7.492 to 9.119	No	ns	0.9969	C	Rot10nM 6h
CTR vs. Rot10nM 24h	6.238	-2.068 to 14.54	No	ns	0.1826	D	Rot10nM 24h
CTR vs. Rot50nM 24h	6.527	-1.778 to 14.83	No	ns	0.1545	E	Rot50nM 24h
**Test details**	**Mean 1**	**Mean 2**	**Mean Diff.**	**SE of diff.**	**n1**	**n2**	**q**	**DF**
CTR vs. Rot10nM 3h	24.71	22.28	2.426	3.186	6	6	0.7614	25
CTR vs. Rot10nM 6h	24.71	23.89	0.8137	3.186	6	6	0.2554	25
CTR vs. Rot10nM 24h	24.71	18.47	6.238	3.186	6	6	1.958	25
CTR vs. Rot50nM 24h	24.71	18.18	6.527	3.186	6	6	2.049	25

Statistics on ATP levels

Normality and Lognormality Tests<br>Tabular results	A<br>CTR	B<br>10-3h	C<br>50-3h	D<br>200-3h	E<br>800-3h	F<br>10-6h	G<br>50-6h	H<br>200-6h	I<br>800-6h	J<br>10-24h	K<br>50-24h	L<br>200-24h	M<br>800-24h
**Shapiro-Wilk test**													
W	0.9523	0.8037	0.8985	0.7997	0.8672	0.9270	0.8969	0.9591	0.9381	0.8822	0.8925	0.9439	0.9171	0.9166
P value	0.7587	0.0634	0.3649	0.0584	0.2154	0.5573	0.3558	0.8132	0.6441	0.2793	0.3315	0.6909	0.4845	0.4809
Passed normality test (alpha=0.05)'	Yes	Yes	Yes	Yes	Yes	Yes	Yes	Yes	Yes	Yes	Yes	Yes	Yes	Yes
P value summary	ns	ns	ns	ns	ns	ns	ns	ns	ns	ns	ns	ns	ns	ns
**Kolmogorov-Smirnov test**														
KS distance	0.2035	0.2941	0.1943	0.2995	0.2753	0.1969	0.2263	0.2381	0.2364	0.2256	0.2847	0.2488	0.1800	0.2447
P value	>0.1000	>0.1000	>0.1000	0.0993	>0.1000	>0.1000	>0.1000	>0.1000	>0.1000	>0.1000	>0.1000	>0.1000	>0.1000	>0.1000
Passed normality test (alpha=0.05)'	Yes	Yes	Yes	Yes	Yes	Yes	Yes	Yes	Yes	Yes	Yes	Yes	Yes	Yes
P value summary	ns	ns	ns	ns	ns	ns	ns	ns	ns	ns	ns	ns	ns	ns
**Number of values**	6	6	6	6	6	6	6	6	6	6	6	6	6	6

	N<br>10-48h	O<br>50-48h	P<br>200-48h	Q<br>800-48h
W	0.9166	0.9149	0.8352	0.8408
P value	0.4809	0.4694	0.1189	0.1324
Passed normality test (alpha=0.05)	Yes	Yes	Yes	Yes
P value summary	ns	ns	ns	ns
KS distance	0.2447	0.1969	0.2760	0.2619
P value	>0.1000	>0.1000	>0.1000	>0.1000
Passed normality test (alpha=0.05)	Yes	Yes	Yes	Yes
P value summary	ns	ns	ns	ns
Number of values	6	6	6	6

Ordinary one-way ANOVA<br>Multiple comparisons							
Number of families	1						
Number of comparisons per family	16						
Alpha	0.05						
**Dunnett's multiple comparisons test**	**Mean Diff.**	**95.00% CI of diff.**	**Significant?**	**Summary**	**Adjusted P Value**	**A-?**	
CTR vs. 10-3h	11.02	1.847 to 20.19	Yes	**	0.0095	B	10-3h
CTR vs. 50-3h	12.46	3.292 to 21.64	Yes	**	0.0022	C	50-3h
CTR vs. 200-3h	56.01	46.84 to 65.19	Yes	****	<0.0001	D	200-3h
CTR vs. 800-3h	61.87	52.70 to 71.05	Yes	****	<0.0001	E	800-3h
CTR vs. 10-6h	12.65	3.478 to 21.82	Yes	**	0.0018	F	10-6h
CTR vs. 50-6h	49.04	39.87 to 58.21	Yes	****	<0.0001	G	50-6h
CTR vs. 200-6h	61.85	52.68 to 71.02	Yes	****	<0.0001	H	200-6h
CTR vs. 800-6h	63.35	54.18 to 72.53	Yes	****	<0.0001	I	800-6h
CTR vs. 10-24h	51.75	42.58 to 60.92	Yes	****	<0.0001	J	10-24h
CTR vs. 50-24h	57.02	47.85 to 66.20	Yes	****	<0.0001	K	50-24h
CTR vs. 200-24h	66.49	57.31 to 75.66	Yes	****	<0.0001	L	200-24h
CTR vs. 800-24h	69.48	60.31 to 78.65	Yes	****	<0.0001	M	800-24h
CTR vs. 10-48h	66.02	56.84 to 75.19	Yes	****	<0.0001	N	10-48h
CTR vs. 50-48h	75.35	66.17 to 84.52	Yes	****	<0.0001	O	50-48h
CTR vs. 200-48h	85.92	76.75 to 95.09	Yes	****	<0.0001	P	200-48h
CTR vs. 800-48h	86.48	77.30 to 95.65	Yes	****	<0.0001	Q	800-48h

**Test details**	**Mean 1**	**Mean 2**	**Mean Diff.**	**SE of diff.**	**n1**	**n2**	**q**	**DF**
CTR vs. 10-3h	100.0	88.98	11.02	3.149	6	6	3.499	85
CTR vs. 50-3h	100.0	87.54	12.46	3.149	6	6	3.957	85
CTR vs. 200-3h	100.0	43.99	56.01	3.149	6	6	17.79	85
CTR vs. 800-3h	100.0	38.13	61.87	3.149	6	6	19.65	85
CTR vs. 10-6h	100.0	87.35	12.65	3.149	6	6	4.017	85
CTR vs. 50-6h	100.0	50.96	49.04	3.149	6	6	15.57	85
CTR vs. 200-6h	100.0	38.15	61.85	3.149	6	6	19.64	85
CTR vs. 800-6h	100.0	36.65	63.35	3.149	6	6	20.12	85
CTR vs. 10-24h	100.0	48.25	51.75	3.149	6	6	16.43	85
CTR vs. 50-24h	100.0	42.98	57.02	3.149	6	6	18.11	85
CTR vs. 200-24h	100.0	33.51	66.49	3.149	6	6	21.11	85
CTR vs. 800-24h	100.0	30.52	69.48	3.149	6	6	22.06	85
CTR vs. 10-48h	100.0	33.99	66.02	3.149	6	6	20.96	85
CTR vs. 50-48h	100.0	24.66	75.35	3.149	6	6	23.92	85
CTR vs. 200-48h	100.0	14.08	85.92	3.149	6	6	27.28	85
CTR vs. 800-48h	100.0	13.53	86.48	3.149	6	6	27.46	85

Statistics on metabolomics data - DHAP

Normality and Lognormality Tests<br>Tabular results	A<br>CTR	B<br>Rot 1d	C<br>Rot 3d	D<br>Rot 7d
**Shapiro-Wilk test**				
W	0.8675	0.9777	0.9099	0.9242
P value	0.2165	0.9394	0.4355	0.5359
Passed normality test (alpha=0.05)?	Yes	Yes	Yes	Yes
P value summary	ns	ns	ns	ns
**Kolmogorov-Smirnov test**				
KS distance	0.2671	0.1646	0.2503	0.2601
P value	>0.1000	>0.1000	>0.1000	>0.1000
Passed normality test (alpha=0.05)?	Yes	Yes	Yes	Yes
P value summary	ns	ns	ns	ns
**Number of values**	6	6	6	6

ANOVA results — Multiple comparisons

ANOVA results ry one-way ANOVA<br>Multiple comparisons								
Number of families	1							
Number of comparisons per family	3							
Alpha	0.05							
**Dunnett's multiple comparisons test**	**Mean Diff.**	**95.00% CI of diff.**	**Significant?**	**Summary**	**Adjusted P Value**	**A-?**		
CTR vs. Rot 1d	-112.6	-185.9 to -39.37	Yes	**	0.0024	B	Rot 1d	
CTR vs. Rot 3d	82.39	9.149 to 155.6	Yes	*	0.0256	C	Rot 3d	
CTR vs. Rot 7d	87.39	14.15 to 160.6	Yes	*	0.0176	D	Rot 7d	
**Test details**	**Mean 1**	**Mean 2**	**Mean Diff.**	**SE of diff.**	**n1**	**n2**	**q**	**DF**
CTR vs. Rot 1d	178.8	291.4	-112.6	28.83	6	6	3.906	20
CTR vs. Rot 3d	178.8	96.39	82.39	28.83	6	6	2.858	20
CTR vs. Rot 7d	178.8	91.39	87.39	28.83	6	6	3.031	20

Statistics on metabolomics data – Gly-3P

Normality and Lognormality Tests<br>Tabular results	A<br>CTR	B<br>Rot 1d	C<br>Rot 3d	D<br>Rot 7d
**Shapiro-Wilk test**				
W	0.8957	0.8999	0.9446	0.9751
P value	0.3490	0.3736	0.6961	0.9246
Passed normality test (alpha=0.05)?	Yes	Yes	Yes	Yes
P value summary	ns	ns	ns	ns
**Kolmogorov-Smirnov test**				
KS distance	0.2091	0.2512	0.2078	0.1576
P value	>0.1000	>0.1000	>0.1000	>0.1000
Passed normality test (alpha=0.05)?	Yes	Yes	Yes	Yes
P value summary	ns	ns	ns	ns
**Number of values**	6	6	6	6

ANOVA results — Multiple comparisons

Ordinary one-way ANOVA<br>Multiple comparisons								
Number of families	1							
Number of comparisons per family	3							
Alpha	0.05							
**Dunnett's multiple comparisons test**	**Mean Diff.**	**95.00% CI of diff.**	**Significant?**	**Summary**	**Adjusted P Value**	**A-?**		
CTR vs. Rot 1d	-740.3	-1150 to -330.7	Yes	***	0.0005	B	Rot 1d	
CTR vs. Rot 3d	431.9	22.35 to 841.5	Yes	*	0.0375	C	Rot 3d	
CTR vs. Rot 7d	525.0	115.5 to 934.6	Yes	*	0.0107	D	Rot 7d	
**Test details**	**Mean 1**	**Mean 2**	**Mean Diff.**	**SE of diff.**	**n1**	**n2**	**q**	**DF**
CTR vs. Rot 1d	792.1	1532	-740.3	161.2	6	6	4.592	20
CTR vs. Rot 3d	792.1	360.2	431.9	161.2	6	6	2.679	20
CTR vs. Rot 7d	792.1	267.1	525.0	161.2	6	6	3.257	20

Statistics on metabolomics data – cis-Aconitate

Normality and Lognormality Tests<br>Tabular results	A<br>CTR	B<br>Rot 1d	C<br>Rot 3d	D<br>Rot 7d
**Shapiro-Wilk test**				
W	0.8115	0.8697	0.9856	0.8862
P value	0.0744	0.2252	0.9759	0.2985
Passed normality test (alpha=0.05)?	Yes	Yes	Yes	Yes
P value summary	ns	ns	ns	ns
**Kolmogorov-Smirnov test**				
KS distance	0.2634	0.3217	0.1649	0.1982
P value	>0.1000	0.0518	>0.1000	>0.1000
Passed normality test (alpha=0.05)?	Yes	Yes	Yes	Yes
P value summary	ns	ns	ns	ns
**Number of values**	6	6	6	6

ANOVA results | Multiple comparisons

Ordinary one-way ANOVA<br>Multiple comparisons								
Number of families	1							
Number of comparisons per family	3							
Alpha	0.05							
**Dunnett's multiple comparisons test**	**Mean Diff.**	**95.00% CI of diff.**	**Significant?**	**Summary**	**Adjusted P Value**	**A-?**		
CTR vs. Rot 1d	-396.2	-643.1 to -149.3	Yes	**	0.0016	B	Rot 1d	
CTR vs. Rot 3d	63.44	-183.4 to 310.3	No	ns	0.8517	C	Rot 3d	
CTR vs. Rot 7d	257.7	10.84 to 504.6	Yes	*	0.0397	D	Rot 7d	
**Test details**	**Mean 1**	**Mean 2**	**Mean Diff.**	**SE of diff.**	**n1**	**n2**	**q**	**DF**
CTR vs. Rot 1d	364.9	761.1	-396.2	97.18	6	6	4.077	20
CTR vs. Rot 3d	364.9	301.4	63.44	97.18	6	6	0.6529	20
CTR vs. Rot 7d	364.9	107.2	257.7	97.18	6	6	2.652	20

Statistics on metabolomics data – Fumarate

Normality and Lognormality Tests<br>Tabular results	A<br>CTR	B<br>Rot 1d	C<br>Rot 3d	D<br>Rot 7d
**Shapiro-Wilk test**				
W	0.9298	0.9476	0.9431	0.8879
P value	0.5783	0.7207	0.6844	0.3072
Passed normality test (alpha=0.05)?	Yes	Yes	Yes	Yes
P value summary	ns	ns	ns	ns
**Kolmogorov-Smirnov test**				
KS distance	0.1747	0.1682	0.1703	0.3139
P value	>0.1000	>0.1000	>0.1000	0.0655
Passed normality test (alpha=0.05)?	Yes	Yes	Yes	Yes
P value summary	ns	ns	ns	ns
**Number of values**	6	6	6	6

ANOVA results | Multiple comparisons

Ordinary one-way ANOVA<br>Multiple comparisons								
Number of families	1							
Number of comparisons per family	3							
Alpha	0.05							
**Dunnett's multiple comparisons test**	**Mean Diff.**	**95.00% CI of diff.**	**Significant?**	**Summary**	**Adjusted P Value**	**A-?**		
CTR vs. Rot 1d	-284.0	-624.6 to 56.58	No	ns	0.1149	B	Rot 1d	
CTR vs. Rot 3d	257.6	-82.97 to 598.2	No	ns	0.1647	C	Rot 3d	
CTR vs. Rot 7d	453.6	113.0 to 794.2	Yes	**	0.0080	D	Rot 7d	
**Test details**	**Mean 1**	**Mean 2**	**Mean Diff.**	**SE of diff.**	**n1**	**n2**	**q**	**DF**
CTR vs. Rot 1d	1014	1298	-284.0	134.1	6	6	2.119	20
CTR vs. Rot 3d	1014	756.5	257.6	134.1	6	6	1.922	20
CTR vs. Rot 7d	1014	560.5	453.6	134.1	6	6	3.384	20

Statistics on metabolomics data – Pyruvate

Normality and Lognormality Tests<br>Tabular results	A<br>CTR	B<br>10nM 1gg	C<br>10nM 3gg	D<br>10nM 7gg
**Shapiro-Wilk test**				
W	0.9298	0.9589	0.9668	0.9262
P value	0.5784	0.8114	0.8704	0.5513
Passed normality test (alpha=0.05)?	Yes	Yes	Yes	Yes
P value summary	ns	ns	ns	ns
**Kolmogorov-Smirnov test**				
KS distance	0.1802	0.1664	0.1667	0.2426
P value	>0.1000	>0.1000	>0.1000	>0.1000
Passed normality test (alpha=0.05)?	Yes	Yes	Yes	Yes
P value summary	ns	ns	ns	ns
**Number of values**	6	6	6	6

ANOVA results | Multiple comparisons

Ordinary one-way ANOVA<br>Multiple comparisons								
Number of families	1							
Number of comparisons per family	3							
Alpha	0.05							
**Dunnett's multiple comparisons test**	**Mean Diff.**	**95.00% CI of diff.**	**Significant?**	**Summary**	**Adjusted P Value**	**A-?**		
CTR vs. 10nM 1gg	-4230	-7917 to -542.4	Yes	*	0.0227	B	10nM 1gg	
CTR vs. 10nM 3gg	1758	-1929 to 5446	No	ns	0.4941	C	10nM 3gg	
CTR vs. 10nM 7gg	4081	393.1 to 7768	Yes	*	0.0283	D	10nM 7gg	
**Test details**	**Mean 1**	**Mean 2**	**Mean Diff.**	**SE of diff.**	**n1**	**n2**	**q**	**DF**
CTR vs. 10nM 1gg	10956	15186	-4230	1451	6	6	2.914	20
CTR vs. 10nM 3gg	10956	9198	1758	1451	6	6	1.211	20
CTR vs. 10nM 7gg	10956	6876	4081	1451	6	6	2.811	20

Statistics on metabolomics data –Citrate

Normality and Lognormality Tests<br>Tabular results	A<br>CTR	B<br>10nM 1gg	C<br>10nM 3gg	D<br>10nM 7gg
**Shapiro-Wilk test**				
W	0.8515	0.8921	0.9472	0.8844
P value	0.1620	0.3294	0.7176	0.2901
Passed normality test (alpha=0.05)?	Yes	Yes	Yes	Yes
P value summary	ns	ns	ns	ns
**Kolmogorov-Smirnov test**				
KS distance	0.2535	0.2094	0.2153	0.2145
P value	>0.1000	>0.1000	>0.1000	>0.1000
Passed normality test (alpha=0.05)?	Yes	Yes	Yes	Yes
P value summary	ns	ns	ns	ns
**Number of values**	6	6	6	6

ANOVA results | Multiple comparisons

Ordinary one-way ANOVA<br>Multiple comparisons								
Number of families	1							
Number of comparisons per family	3							
Alpha	0.05							
**Dunnett's multiple comparisons test**	**Mean Diff.**	**95.00% CI of diff.**	**Significant?**	**Summary**	**Adjusted P Value**	**A-?**		
CTR vs. 10nM 1gg	-5892	-10826 to -957.5	Yes	*	0.0175	B	10nM 1gg	
CTR vs. 10nM 3gg	5381	446.3 to 10315	Yes	*	0.0309	C	10nM 3gg	
CTR vs. 10nM 7gg	6595	1660 to 11529	Yes	**	0.0078	D	10nM 7gg	
**Test details**	**Mean 1**	**Mean 2**	**Mean Diff.**	**SE of diff.**	**n1**	**n2**	**q**	**DF**
CTR vs. 10nM 1gg	9366	15258	-5892	1942	6	6	3.034	20
CTR vs. 10nM 3gg	9366	3985	5381	1942	6	6	2.770	20
CTR vs. 10nM 7gg	9366	2771	6595	1942	6	6	3.395	20

Statistics on metabolomics data – αKG

Normality and Lognormality Tests<br>Tabular results	A<br>CTR	B<br>10nM 1gg	C<br>10nM 3gg	D<br>10nM 7gg
**Shapiro-Wilk test**				
W	0.8002	0.8923	0.9248	0.8613
P value	0.0590	0.3301	0.5403	0.1938
Passed normality test (alpha=0.05)?	Yes	Yes	Yes	Yes
P value summary	ns	ns	ns	ns
**Kolmogorov-Smirnov test**				
KS distance	0.2811	0.2087	0.2556	0.2390
P value	>0.1000	>0.1000	>0.1000	>0.1000
Passed normality test (alpha=0.05)?	Yes	Yes	Yes	Yes
P value summary	ns	ns	ns	ns
**Number of values**	6	6	6	6

ANOVA results — Multiple comparisons

Ordinary one-way ANOVA<br>Multiple comparisons								
Number of families	1							
Number of comparisons per family	3							
Alpha	0.05							
**Dunnett's multiple comparisons test**	**Mean Diff.**	**95.00% CI of diff.**	**Significant?**	**Summary**	**Adjusted P Value**	**A-?**		
CTR vs. 10nM 1gg	-6152	-10985 to -1319	Yes	*	0.0112	B	10nM 1gg	
CTR vs. 10nM 3gg	4949	116.5 to 9783	Yes	*	0.0440	C	10nM 3gg	
CTR vs. 10nM 7gg	6282	1449 to 11115	Yes	**	0.0096	D	10nM 7gg	
**Test details**	**Mean 1**	**Mean 2**	**Mean Diff.**	**SE of diff.**	**n1**	**n2**	**q**	**DF**
CTR vs. 10nM 1gg	9102	15254	-6152	1902	6	6	3.234	20
CTR vs. 10nM 3gg	9102	4152	4949	1902	6	6	2.602	20
CTR vs. 10nM 7gg	9102	2820	6282	1902	6	6	3.302	20

Statistics on metabolomics data – L-Malate

Normality and Lognormality Tests<br>Tabular results	A<br>CTR	B<br>10nM 1gg	C<br>10nM 3gg	D<br>10nM 7gg
**Shapiro-Wilk test**				
W	0.9439	0.9563	0.9674	0.9313
P value	0.6905	0.7908	0.8746	0.5905
Passed normality test (alpha=0.05)?	Yes	Yes	Yes	Yes
P value summary	ns	ns	ns	ns
**Kolmogorov-Smirnov test**				
KS distance	0.1992	0.1667	0.1666	0.1683
P value	>0.1000	>0.1000	>0.1000	>0.1000
Passed normality test (alpha=0.05)?	Yes	Yes	Yes	Yes
P value summary	ns	ns	ns	ns
**Number of values**	6	6	6	6

ANOVA results — Multiple comparisons

Ordinary one-way ANOVA<br>Multiple comparisons								
Number of families	1							
Number of comparisons per family	3							
Alpha	0.05							
**Dunnett's multiple comparisons test**	**Mean Diff.**	**95.00% CI of diff.**	**Significant?**	**Summary**	**Adjusted P Value**	**A-?**		
CTR vs. 10nM 1gg	-4551	-8093 to -1008	Yes	*	0.0105	B	10nM 1gg	
CTR vs. 10nM 3gg	1412	-2130 to 4955	No	ns	0.6240	C	10nM 3gg	
CTR vs. 10nM 7gg	2899	-643.0 to 6442	No	ns	0.1236	D	10nM 7gg	
**Test details**	**Mean 1**	**Mean 2**	**Mean Diff.**	**SE of diff.**	**n1**	**n2**	**q**	**DF**
CTR vs. 10nM 1gg	10587	15138	-4551	1394	6	6	3.264	20
CTR vs. 10nM 3gg	10587	9175	1412	1394	6	6	1.013	20
CTR vs. 10nM 7gg	10587	7688	2899	1394	6	6	2.079	20

Statistics on metabolomics data – Ac-CoA

Normality and Lognormality Tests<br>Tabular results	A<br>CTR	B<br>10nM 1gg	C<br>10nM 3gg	D<br>10nM 7gg
**Shapiro-Wilk test**				
W	0.9606	0.8593	0.9422	0.8565
P value	0.8246	0.1868	0.6771	0.1774
Passed normality test (alpha=0.05)?	Yes	Yes	Yes	Yes
P value summary	ns	ns	ns	ns
**Kolmogorov-Smirnov test**				
KS distance	0.2215	0.3054	0.2533	0.2576
P value	>0.1000	0.0841	>0.1000	>0.1000
Passed normality test (alpha=0.05)?	Yes	Yes	Yes	Yes
P value summary	ns	ns	ns	ns
**Number of values**	6	6	6	6

ANVOA results — Multiple comparisons

Ordinary one-way ANOVA<br>Multiple comparisons								
Number of families	1							
Number of comparisons per family	3							
Alpha	0.05							
**Dunnett's multiple comparisons test**	**Mean Diff.**	**95.00% CI of diff.**	**Significant?**	**Summary**	**Adjusted P Value**	**A-?**		
CTR vs. 10nM 1gg	24.81	7.836 to 41.77	Yes	**	0.0038	B	10nM 1gg	
CTR vs. 10nM 3gg	21.84	4.869 to 38.81	Yes	*	0.0104	C	10nM 3gg	
CTR vs. 10nM 7gg	23.00	6.031 to 39.97	Yes	**	0.0070	D	10nM 7gg	
**Test details**	**Mean 1**	**Mean 2**	**Mean Diff.**	**SE of diff.**	**n1**	**n2**	**q**	**DF**
CTR vs. 10nM 1gg	58.06	33.25	24.81	6.679	6	6	3.714	20
CTR vs. 10nM 3gg	58.06	36.22	21.84	6.679	6	6	3.270	20
CTR vs. 10nM 7gg	58.06	35.06	23.00	6.679	6	6	3.443	20

Statistics on metabolomics data – Succinate

Normality and Lognormality Tests<br>Tabular results	A<br>CTR	B<br>10nM 1gg	C<br>10nM 3gg	D<br>10nM 7gg
**Shapiro-Wilk test**				
W	0.9530	0.9663	0.9245	0.9204
P value	0.7642	0.8670	0.5387	0.5080
Passed normality test (alpha=0.05)?	Yes	Yes	Yes	Yes
P value summary	ns	ns	ns	ns
**Kolmogorov-Smirnov test**				
KS distance	0.1924	0.2016	0.2682	0.2390
P value	>0.1000	>0.1000	>0.1000	>0.1000
Passed normality test (alpha=0.05)?	Yes	Yes	Yes	Yes
P value summary	ns	ns	ns	ns
**Number of values**	6	6	6	6

ANOVA results — Multiple comparisons

Ordinary one-way ANOVA<br>Multiple comparisons								
Number of families	1							
Number of comparisons per family	3							
Alpha	0.05							
**Dunnett's multiple comparisons test**	**Mean Diff.**	**95.00% CI of diff.**	**Significant?**	**Summary**	**Adjusted P Value**	**A-?**		
CTR vs. 10nM 1gg	19.45	-5.384 to 44.28	No	ns	0.1458	B	10nM 1gg	
CTR vs. 10nM 3gg	52.89	28.06 to 77.72	Yes	****	<0.0001	C	10nM 3gg	
CTR vs. 10nM 7gg	55.17	30.34 to 80.00	Yes	****	<0.0001	D	10nM 7gg	
**Test details**	**Mean 1**	**Mean 2**	**Mean Diff.**	**SE of diff.**	**n1**	**n2**	**q**	**DF**
CTR vs. 10nM 1gg	101.7	82.28	19.45	9.773	6	6	1.990	20
CTR vs. 10nM 3gg	101.7	48.83	52.89	9.773	6	6	5.412	20
CTR vs. 10nM 7gg	101.7	46.56	55.17	9.773	6	6	5.645	20

Statistics on metabolomics data – Ru5P

Normality and Lognormality Tests<br>Tabular results	CTR	10nM 1gg	10nM 3gg	10nM 7gg
P value				
Passed normality test (alpha=0.05)?				
P value summary				
**Shapiro-Wilk test**				
W	0.9083	0.8953	0.8084	0.9218
P value	0.4250	0.3469	0.0698	0.5181
Passed normality test (alpha=0.05)?	Yes	Yes	Yes	Yes
P value summary	ns	ns	ns	ns
**Kolmogorov-Smirnov test**				
KS distance	0.2602	0.2753	0.3161	0.2039
P value	>0.1000	>0.1000	0.0614	>0.1000
Passed normality test (alpha=0.05)?	Yes	Yes	Yes	Yes
P value summary	ns	ns	ns	ns
**Number of values**	6	6	6	6

ANOVA results | Multiple comparisons

Ordinary one-way ANOVA<br>Multiple comparisons								
Number of families	1							
Number of comparisons per family	3							
Alpha	0.05							
**Dunnett's multiple comparisons test**	**Mean Diff.**	**95.00% CI of diff.**	**Significant?**	**Summary**	**Adjusted P Value**	**A-?**		
CTR vs. 10nM 1gg	-55.78	-138.4 to 26.82	No	ns	0.2348	B	10nM 1gg	
CTR vs. 10nM 3gg	87.61	5.012 to 170.2	Yes	*	0.0363	C	10nM 3gg	
CTR vs. 10nM 7gg	85.56	2.957 to 168.2	Yes	*	0.0414	D	10nM 7gg	
**Test details**	**Mean 1**	**Mean 2**	**Mean Diff.**	**SE of diff.**	**n1**	**n2**	**q**	**DF**
CTR vs. 10nM 1gg	146.2	201.9	-55.78	32.51	6	6	1.716	20
CTR vs. 10nM 3gg	146.2	58.56	87.61	32.51	6	6	2.695	20
CTR vs. 10nM 7gg	146.2	60.61	85.56	32.51	6	6	2.632	20

Statistics on metabolomics data – D-ribose

Normality and Lognormality Tests<br>Tabular results	CTR	10nM 1gg	10nM 3gg	10nM 7gg
**Shapiro-Wilk test**				
W	0.9666	0.9577	0.9036	0.9372
P value	0.8688	0.8020	0.3958	0.6368
Passed normality test (alpha=0.05)?	Yes	Yes	Yes	Yes
P value summary	ns	ns	ns	ns
**Kolmogorov-Smirnov test**				
KS distance	0.1582	0.2270	0.1995	0.1753
P value	>0.1000	>0.1000	>0.1000	>0.1000
Passed normality test (alpha=0.05)?	Yes	Yes	Yes	Yes
P value summary	ns	ns	ns	ns
**Number of values**	6	6	6	6

ANOVA results | Multiple comparisons

Ordinary one-way ANOVA<br>Multiple comparisons								
Number of families	1							
Number of comparisons per family	3							
Alpha	0.05							
**Dunnett's multiple comparisons test**	**Mean Diff.**	**95.00% CI of diff.**	**Significant?**	**Summary**	**Adjusted P Value**	**A-?**		
CTR vs. 10nM 1gg	1.108	-59.33 to 61.55	No	ns	>0.9999	B	10nM 1gg	
CTR vs. 10nM 3gg	118.9	58.45 to 179.3	Yes	***	0.0002	C	10nM 3gg	
CTR vs. 10nM 7gg	125.8	65.33 to 186.2	Yes	***	0.0001	D	10nM 7gg	
**Test details**	**Mean 1**	**Mean 2**	**Mean Diff.**	**SE of diff.**	**n1**	**n2**	**q**	**DF**
CTR vs. 10nM 1gg	242.1	241.0	1.108	23.79	6	6	0.04656	20
CTR vs. 10nM 3gg	242.1	123.2	118.9	23.79	6	6	4.997	20
CTR vs. 10nM 7gg	242.1	116.3	125.8	23.79	6	6	5.287	20

Statistics on metabolomics data – S7P

Normality and Lognormality Tests Tabular results	A CTR	B 10nM 1gg	C 10nM 3gg	D 10nM 7gg
**Shapiro-Wilk test**				
W	0.8756	0.9847	0.7993	0.8760
P value	0.2494	0.9722	0.0580	0.2512
Passed normality test (alpha=0.05)?	Yes	Yes	Yes	Yes
P value summary	ns	ns	ns	ns
**Kolmogorov-Smirnov test**				
KS distance	0.2474	0.1708	0.3206	0.2075
P value	>0.1000	>0.1000	0.0536	>0.1000
Passed normality test (alpha=0.05)?	Yes	Yes	Yes	Yes
P value summary	ns	ns	ns	ns
**Number of values**	6	6	6	6

ANOVA results | Multiple comparisons

Ordinary one-way ANOVA Multiple comparisons								
Number of families	1							
Number of comparisons per family	3							
Alpha	0.05							
**Dunnett's multiple comparisons test**	**Mean Diff.**	**95.00% CI of diff.**	**Significant?**	**Summary**	**Adjusted P Value**	**A-?**		
CTR vs. 10nM 1gg	-50.94	-238.7 to 136.8	No	ns	0.8315	B	10nM 1gg	
CTR vs. 10nM 3gg	236.4	48.69 to 424.2	Yes	*	0.0121	C	10nM 3gg	
CTR vs. 10nM 7gg	253.9	66.20 to 441.7	Yes	**	0.0071	D	10nM 7gg	
**Test details**	**Mean 1**	**Mean 2**	**Mean Diff.**	**SE of diff.**	**n1**	**n2**	**q**	**DF**
CTR vs. 10nM 1gg	352.7	403.6	-50.94	73.89	6	6	0.6894	20
CTR vs. 10nM 3gg	352.7	116.2	236.4	73.89	6	6	3.200	20
CTR vs. 10nM 7gg	352.7	98.73	253.9	73.89	6	6	3.436	20

Statistics on metabolomics data – NAD^+/NADH

Tabular results

Normality and Lognormality Tests Tabular results	A CTR	B 10nM 6h	C 10nM 1gg	D 10nM 3gg	E 10nM 7gg
**Shapiro-Wilk test**					
W	0.9629	0.9076	0.9103	0.9555	0.9672
P value	0.8418	0.4207	0.4381	0.7847	0.8731
Passed normality test (alpha=0.05)?	Yes	Yes	Yes	Yes	Yes
P value summary	ns	ns	ns	ns	ns
**Kolmogorov-Smirnov test**					
KS distance	0.1893	0.2918	0.2073	0.1677	0.1983
P value	>0.1000	>0.1000	>0.1000	>0.1000	>0.1000
Passed normality test (alpha=0.05)?	Yes	Yes	Yes	Yes	Yes
P value summary	ns	ns	ns	ns	ns
**Number of values**	6	6	6	6	6

ANOVA results | Multiple comparisons

Ordinary one-way ANOVA Multiple comparisons								
Number of families	1							
Number of comparisons per family	4							
Alpha	0.05							
**Dunnett's multiple comparisons test**	**Mean Diff.**	**95.00% CI of diff.**	**Significant?**	**Summary**	**Adjusted P Value**	**A-?**		
CTR vs. 10nM 6h	0.5917	-0.3119 to 1.495	No	ns	0.2814	B	10nM 6h	
CTR vs. 10nM 1gg	1.115	0.2115 to 2.019	Yes	*	0.0125	C	10nM 1gg	
CTR vs. 10nM 3gg	1.345	0.4415 to 2.249	Yes	**	0.0025	D	10nM 3gg	
CTR vs. 10nM 7gg	1.308	0.4048 to 2.212	Yes	**	0.0032	E	10nM 7gg	
**Test details**	**Mean 1**	**Mean 2**	**Mean Diff.**	**SE of diff.**	**n1**	**n2**	**q**	**DF**
CTR vs. 10nM 6h	3.165	2.573	0.5917	0.3466	6	6	1.707	25
CTR vs. 10nM 1gg	3.165	2.050	1.115	0.3466	6	6	3.217	25
CTR vs. 10nM 3gg	3.165	1.820	1.345	0.3466	6	6	3.881	25
CTR vs. 10nM 7gg	3.165	1.857	1.308	0.3466	6	6	3.775	25

Statistics on metabolomics data – MVA

Normality and Lognormality Tests Tabular results	A CTR	B 10nM 1gg	C 10nM 3gg	D 10nM 7gg
**Shapiro-Wilk test**				
W	0.9171	0.8976	0.8219	0.9537
P value	0.4845	0.3600	0.0917	0.7703
Passed normality test (alpha=0.05)?	Yes	Yes	Yes	Yes
P value summary	ns	ns	ns	ns
**Kolmogorov-Smirnov test**				
KS distance	0.2348	0.1909	0.2555	0.2349
P value	>0.1000	>0.1000	>0.1000	>0.1000
Passed normality test (alpha=0.05)?	Yes	Yes	Yes	Yes
P value summary	ns	ns	ns	ns
**Number of values**	6	6	6	6

ANOVA results | Multiple comparisons

Ordinary one-way ANOVA Multiple comparisons								
Number of families	1							
Number of comparisons per family	3							
Alpha	0.05							
**Dunnett's multiple comparisons test**	**Mean Diff.**	**95.00% CI of diff.**	**Significant?**	**Summary**	**Adjusted P Value**	**A-?**		
CTR vs. 10nM 1gg	2.416	-15.56 to 20.39	No	ns	0.9732	B	10nM 1gg	
CTR vs. 10nM 3gg	21.17	3.192 to 39.14	Yes	*	0.0192	C	10nM 3gg	
CTR vs. 10nM 7gg	29.69	11.72 to 47.67	Yes	**	0.0012	D	10nM 7gg	
**Test details**	**Mean 1**	**Mean 2**	**Mean Diff.**	**SE of diff.**	**n1**	**n2**	**q**	**DF**
CTR vs. 10nM 1gg	58.03	55.61	2.416	7.075	6	6	0.3414	20
CTR vs. 10nM 3gg	58.03	36.86	21.17	7.075	6	6	2.992	20
CTR vs. 10nM 7gg	58.03	28.33	29.69	7.075	6	6	4.197	20

Statistics on metabolomics data – MVA-P

Normality and Lognormality Tests Tabular results	A CTR	B 10nM 1gg	C 10nM 3gg	D 10nM 7gg
**Shapiro-Wilk test**				
W	0.8601	0.7932	0.8528	0.8524
P value	0.1895	0.0510	0.1657	0.1645
Passed normality test (alpha=0.05)?	Yes	Yes	Yes	Yes
P value summary	ns	ns	ns	ns
**Kolmogorov-Smirnov test**				
KS distance	0.2789	0.3127	0.2271	0.2619
P value	>0.1000	0.0679	>0.1000	>0.1000
Passed normality test (alpha=0.05)?	Yes	Yes	Yes	Yes
P value summary	ns	ns	ns	ns
**Number of values**	6	6	6	6

ANOVA results | Multiple comparisons

Ordinary one-way ANOVA Multiple comparisons								
Number of families	1							
Number of comparisons per family	3							
Alpha	0.05							
**Dunnett's multiple comparisons test**	**Mean Diff.**	**95.00% CI of diff.**	**Significant?**	**Summary**	**Adjusted P Value**	**A-?**		
CTR vs. 10nM 1gg	-8.527	-33.24 to 16.18	No	ns	0.7150	B	10nM 1gg	
CTR vs. 10nM 3gg	29.03	4.318 to 53.74	Yes	*	0.0195	C	10nM 3gg	
CTR vs. 10nM 7gg	36.83	12.12 to 61.54	Yes	**	0.0032	D	10nM 7gg	
**Test details**	**Mean 1**	**Mean 2**	**Mean Diff.**	**SE of diff.**	**n1**	**n2**	**q**	**DF**
CTR vs. 10nM 1gg	68.08	76.61	-8.527	9.726	6	6	0.8767	20
CTR vs. 10nM 3gg	68.08	39.06	29.03	9.726	6	6	2.985	20
CTR vs. 10nM 7gg	68.08	31.25	36.83	9.726	6	6	3.787	20

Statistics on metabolomics data – GPP

Normality and Lognormality Tests<br>Tabular results	A<br>CTR	B<br>10nM 1gg	C<br>10nM 3gg	D<br>10nM 7gg
**Shapiro-Wilk test**				
W	0.8766	0.9864	0.8249	0.8604
P value	0.2540	0.9787	0.0973	0.1906
Passed normality test (alpha=0.05)?	Yes	Yes	Yes	Yes
P value summary	ns	ns	ns	ns
**Kolmogorov-Smirnov test**				
KS distance	0.2017	0.1592	0.2776	0.2118
P value	>0.1000	>0.1000	>0.1000	>0.1000
Passed normality test (alpha=0.05)?	Yes	Yes	Yes	Yes
P value summary	ns	ns	ns	ns
**Number of values**	6	6	6	6

ANOVA results | Multiple comparisons

Ordinary one-way ANOVA<br>Multiple comparisons								
Number of families	1							
Number of comparisons per family	3							
Alpha	0.05							
**Dunnett's multiple comparisons test**	**Mean Diff.**	**95.00% CI of diff.**	**Significant?**	**Summary**	**Adjusted P Value**	**A-?**		
CTR vs. 10nM 1gg	20.89	-42.09 to 83.87	No	ns	0.7373	B	10nM 1gg	
CTR vs. 10nM 3gg	74.94	11.97 to 137.9	Yes	*	0.0179	C	10nM 3gg	
CTR vs. 10nM 7gg	86.03	23.05 to 149.0	Yes	**	0.0066	D	10nM 7gg	
**Test details**	**Mean 1**	**Mean 2**	**Mean Diff.**	**SE of diff.**	**n1**	**n2**	**q**	**DF**
CTR vs. 10nM 1gg	122.6	101.7	20.89	24.79	6	6	0.8427	20
CTR vs. 10nM 3gg	122.6	47.61	74.94	24.79	6	6	3.023	20
CTR vs. 10nM 7gg	122.6	36.53	86.03	24.79	6	6	3.470	20

Statistics on metabolomics data – GGPP

Normality and Lognormality Tests<br>Tabular results	A<br>CTR	B<br>10nM 1gg	C<br>10nM 3gg	D<br>10nM 7gg
**Shapiro-Wilk test**				
W	0.9390	0.8651	0.8600	0.9726
P value	0.6513	0.2073	0.1892	0.9093
Passed normality test (alpha=0.05)?	Yes	Yes	Yes	Yes
P value summary	ns	ns	ns	ns
**Kolmogorov-Smirnov test**				
KS distance	0.1928	0.2528	0.2966	0.1815
P value	>0.1000	>0.1000	>0.1000	>0.1000
Passed normality test (alpha=0.05)?	Yes	Yes	Yes	Yes
P value summary	ns	ns	ns	ns
**Number of values**	6	6	6	6

ANOVA results | Multiple comparisons

Ordinary one-way ANOVA<br>Multiple comparisons								
Number of families	1							
Number of comparisons per family	3							
Alpha	0.05							
**Sidak's multiple comparisons test**	**Mean Diff.**	**95.00% CI of diff.**	**Significant?**	**Summary**	**Adjusted P Value**	**A-?**		
CTR vs. 10nM 1gg	59.58	48.10 to 71.07	Yes	****	<0.0001	B	10nM 1gg	
CTR vs. 10nM 3gg	47.08	35.60 to 58.57	Yes	****	<0.0001	C	10nM 3gg	
CTR vs. 10nM 7gg	70.33	58.85 to 81.82	Yes	****	<0.0001	D	10nM 7gg	
**Test details**	**Mean 1**	**Mean 2**	**Mean Diff.**	**SE of diff.**	**n1**	**n2**	**t**	**DF**
CTR vs. 10nM 1gg	113.6	53.97	59.58	4.409	6	6	13.52	20
CTR vs. 10nM 3gg	113.6	66.47	47.08	4.409	6	6	10.68	20
CTR vs. 10nM 7gg	113.6	43.22	70.33	4.409	6	6	15.95	20

Statistics on metabolomics data – Gluc/L-Lact

Normality and Lognormality Tests<br>Tabular results	A<br>CTR	B<br>10nM 1d	C<br>10nM 3d	D<br>10nM 7d
**Shapiro-Wilk test**				
W	0.8639	0.8579	0.9596	0.9308
P value	0.2028	0.1821	0.8163	0.5863
Passed normality test (alpha=0.05)?	Yes	Yes	Yes	Yes
P value summary	ns	ns	ns	ns
**Kolmogorov-Smirnov test**				
KS distance	0.2535	0.2376	0.1960	0.1954
P value	>0.1000	>0.1000	>0.1000	>0.1000
Passed normality test (alpha=0.05)?	Yes	Yes	Yes	Yes
P value summary	ns	ns	ns	ns
**Number of values**	6	6	6	6

ANOVA results | Multiple comparisons

Ordinary one-way ANOVA<br>Multiple comparisons								
Number of families	1							
Number of comparisons per family	3							
Alpha	0.05							
**Dunnett's multiple comparisons test**	**Mean Diff.**	**95.00% CI of diff.**	**Significant?**	**Summary**	**Adjusted P Value**	**A-?**		
CTR vs. 10nM 1d	0.06895	-0.5557 to 0.6936	No	ns	0.9846	B	10nM 1d	
CTR vs. 10nM 3d	0.4233	-0.2014 to 1.048	No	ns	0.2325	C	10nM 3d	
CTR vs. 10nM 7d	0.8083	0.1836 to 1.433	Yes	**	0.0100	D	10nM 7d	
**Test details**	**Mean 1**	**Mean 2**	**Mean Diff.**	**SE of diff.**	**n1**	**n2**	**q**	**DF**
CTR vs. 10nM 1d	1.442	1.373	0.06895	0.2459	6	6	0.2804	20
CTR vs. 10nM 3d	1.442	1.018	0.4233	0.2459	6	6	1.722	20
CTR vs. 10nM 7d	1.442	0.6333	0.8083	0.2459	6	6	3.287	20

Statistics on metabolomics data – Gluc/Pyr

Normality and Lognormality Tests<br>Tabular results	A<br>CTR	B<br>10nM 1d	C<br>10nM 3d	D<br>10nM 7d
**Shapiro-Wilk test**				
W	0.9156	0.7965	0.9155	0.9375
P value	0.4745	0.0547	0.4733	0.6390
Passed normality test (alpha=0.05)?	Yes	Yes	Yes	Yes
P value summary	ns	ns	ns	ns
**Kolmogorov-Smirnov test**				
KS distance	0.2744	0.3196	0.2932	0.2321
P value	>0.1000	0.0553	>0.1000	>0.1000
Passed normality test (alpha=0.05)?	Yes	Yes	Yes	Yes
P value summary	ns	ns	ns	ns
**Number of values**	6	6	6	6

ANOVA results | Multiple comparisons

Ordinary one-way ANOVA<br>Multiple comparisons								
Number of families	1							
Number of comparisons per family	3							
Alpha	0.05							
**Dunnett's multiple comparisons test**	**Mean Diff.**	**95.00% CI of diff.**	**Significant?**	**Summary**	**Adjusted P Value**	**A-?**		
CTR vs. 10nM 1d	0.3100	0.1455 to 0.4745	Yes	***	0.0003	B	10nM 1d	
CTR vs. 10nM 3d	0.3803	0.2159 to 0.5448	Yes	****	<0.0001	C	10nM 3d	
CTR vs. 10nM 7d	0.3537	0.1892 to 0.5181	Yes	****	<0.0001	D	10nM 7d	
**Test details**	**Mean 1**	**Mean 2**	**Mean Diff.**	**SE of diff.**	**n1**	**n2**	**q**	**DF**
CTR vs. 10nM 1d	0.8937	0.5837	0.3100	0.06473	6	6	4.789	20
CTR vs. 10nM 3d	0.8937	0.5133	0.3803	0.06473	6	6	5.876	20
CTR vs. 10nM 7d	0.8937	0.5400	0.3537	0.06473	6	6	5.464	20

Statistics on metabolomics data – Pyr/L-Mal

Normality and Lognormality Tests<br>Tabular results	A<br>CTR	B<br>10nM 1d	C<br>10nM 3d	D<br>10nM 7d
**Shapiro-Wilk test**				
W	0.7791	0.9124	0.8589	0.8379
P value	0.0541	0.4522	0.1856	0.1253
Passed normality test (alpha=0.05)?	Yes	Yes	Yes	Yes
P value summary	ns	ns	ns	ns
**Kolmogorov-Smirnov test**				
KS distance	0.3290	0.1724	0.2638	0.2502
P value	0.0815	>0.1000	>0.1000	>0.1000
Passed normality test (alpha=0.05)?	Yes	Yes	Yes	Yes
P value summary	ns	ns	ns	ns
**Number of values**	5	6	6	6

ANOVA results | Multiple comparisons

Ordinary one-way ANOVA<br>Multiple comparisons								
Number of families	1							
Number of comparisons per family	3							
Alpha	0.05							
**Dunnett's multiple comparisons test**	**Mean Diff.**	**95.00% CI of diff.**	**Significant?**	**Summary**	**Adjusted P Value**	**A-?**		
CTR vs. 10nM 1d	0.004133	-0.007522 to 0.01579	No	ns	0.6888	B	10nM 1d	
CTR vs. 10nM 3d	-0.002367	-0.01402 to 0.009288	No	ns	0.9129	C	10nM 3d	
CTR vs. 10nM 7d	0.0008000	-0.01086 to 0.01246	No	ns	0.9959	D	10nM 7d	
**Test details**	**Mean 1**	**Mean 2**	**Mean Diff.**	**SE of diff.**	**n1**	**n2**	**q**	**DF**
CTR vs. 10nM 1d	1.007	1.003	0.004133	0.004589	5	6	0.9006	19
CTR vs. 10nM 3d	1.007	1.010	-0.002367	0.004589	5	6	0.5157	19
CTR vs. 10nM 7d	1.007	1.007	0.0008000	0.004589	5	6	0.1743	19

Statistics on metabolomics data – Pyr+L-Mal

Normality and Lognormality Tests<br>Tabular results	A<br>CTR	B<br>10nM 1d	C<br>10nM 3d	D<br>10nM 7d
**Shapiro-Wilk test**				
W	0.9091	0.9577	0.9334	0.9008
P value	0.4305	0.8019	0.6067	0.3784
Passed normality test (alpha=0.05)?	Yes	Yes	Yes	Yes
P value summary	ns	ns	ns	ns
**Kolmogorov-Smirnov test**				
KS distance	0.2459	0.1663	0.1881	0.2364
P value	>0.1000	>0.1000	>0.1000	>0.1000
Passed normality test (alpha=0.05)?	Yes	Yes	Yes	Yes
P value summary	ns	ns	ns	ns
**Number of values**	6	6	6	6

ANOVA results | Multiple comparisons

Ordinary one-way ANOVA<br>Multiple comparisons								
Number of families	1							
Number of comparisons per family	3							
Alpha	0.05							
**Dunnett's multiple comparisons test**	**Mean Diff.**	**95.00% CI of diff.**	**Significant?**	**Summary**	**Adjusted P Value**	**A-?**		
CTR vs. 10nM 1d	-5113	-15054 to 4827	No	ns	0.4358	B	10nM 1d	
CTR vs. 10nM 3d	10066	125.9 to 20006	Yes	*	0.0468	C	10nM 3d	
CTR vs. 10nM 7d	10824	883.6 to 20764	Yes	*	0.0312	D	10nM 7d	
**Test details**	**Mean 1**	**Mean 2**	**Mean Diff.**	**SE of diff.**	**n1**	**n2**	**q**	**DF**
CTR vs. 10nM 1d	25210	30323	-5113	3913	6	6	1.307	20
CTR vs. 10nM 3d	25210	15144	10066	3913	6	6	2.573	20
CTR vs. 10nM 7d	25210	14386	10824	3913	6	6	2.766	20

Statistics on metabolomics data – Riboflavin

Normality and Lognormality Tests<br>Tabular results	A<br>CTR	B<br>10nM 1gg	C<br>10nM 3gg	D<br>10nM 7gg
**Shapiro-Wilk test**				
W	0.9809	0.9459	0.8894	0.9198
P value	0.9557	0.7073	0.3149	0.5043
Passed normality test (alpha=0.05)?	Yes	Yes	Yes	Yes
P value summary	ns	ns	ns	ns
**Kolmogorov-Smirnov test**				
KS distance	0.1376	0.2410	0.2083	0.2422
P value	>0.1000	>0.1000	>0.1000	>0.1000
Passed normality test (alpha=0.05)?	Yes	Yes	Yes	Yes
P value summary	ns	ns	ns	ns
**Number of values**	6	6	6	6

ANVOA results | Multiple comparisons

Ordinary one-way ANOVA<br>Multiple comparisons								
Number of families	1							
Number of comparisons per family	3							
Alpha	0.05							
**Dunnett's multiple comparisons test**	**Mean Diff.**	**95.00% CI of diff.**	**Significant?**	**Summary**	**Adjusted P Value**	**A-?**		
CTR vs. 10nM 1gg	8.367	-17.82 to 34.55	No	ns	0.7572	B	10nM 1gg	
CTR vs. 10nM 3gg	31.28	5.096 to 57.47	Yes	*	0.0174	C	10nM 3gg	
CTR vs. 10nM 7gg	47.80	21.61 to 73.98	Yes	***	0.0005	D	10nM 7gg	
**Test details**	**Mean 1**	**Mean 2**	**Mean Diff.**	**SE of diff.**	**n1**	**n2**	**q**	**DF**
CTR vs. 10nM 1gg	106.7	98.30	8.367	10.31	6	6	0.8117	20
CTR vs. 10nM 3gg	106.7	75.38	31.28	10.31	6	6	3.035	20
CTR vs. 10nM 7gg	106.7	58.87	47.80	10.31	6	6	4.637	20

Statistics on metabolomics data – Reduced Riboflavin

Normality and Lognormality Tests<br>Tabular results	A<br>CTR	B<br>10nM 1gg	C<br>10nM 3gg	D<br>10nM 7gg
**Shapiro-Wilk test**				
W	0.8782	0.9586	0.8998	0.9651
P value	0.2610	0.8088	0.3725	0.8580
Passed normality test (alpha=0.05)?	Yes	Yes	Yes	Yes
P value summary	ns	ns	ns	ns
**Kolmogorov-Smirnov test**				
KS distance	0.2523	0.1658	0.2255	0.1879
P value	>0.1000	>0.1000	>0.1000	>0.1000
Passed normality test (alpha=0.05)?	Yes	Yes	Yes	Yes
P value summary	ns	ns	ns	ns
**Number of values**	6	6	6	6

ANOVA results | Multiple comparisons

Ordinary one-way ANOVA<br>Multiple comparisons								
Number of families	1							
Number of comparisons per family	3							
Alpha	0.05							
**Dunnett's multiple comparisons test**	**Mean Diff.**	**95.00% CI of diff.**	**Significant?**	**Summary**	**Adjusted P Value**	**A-?**		
CTR vs. 10nM 1gg	164.1	63.26 to 264.9	Yes	**	0.0014	B	10nM 1gg	
CTR vs. 10nM 3gg	195.2	94.40 to 296.0	Yes	***	0.0002	C	10nM 3gg	
CTR vs. 10nM 7gg	224.4	123.6 to 325.2	Yes	****	<0.0001	D	10nM 7gg	
**Test details**	**Mean 1**	**Mean 2**	**Mean Diff.**	**SE of diff.**	**n1**	**n2**	**q**	**DF**
CTR vs. 10nM 1gg	340.2	176.2	164.1	39.67	6	6	4.135	20
CTR vs. 10nM 3gg	340.2	145.0	195.2	39.67	6	6	4.920	20
CTR vs. 10nM 7gg	340.2	115.8	224.4	39.67	6	6	5.656	20

Statistics on metabolomics data – Glutathione

Normality and Lognormality Tests Tabular results	A CTR	B 10nM 1gg	C 10nM 3gg	D 10nM 7gg
**Shapiro-Wilk test**				
W	0.9054	0.9312	0.9696	0.9504
P value	0.4066	0.5894	0.8895	0.7435
Passed normality test (alpha=0.05)?	Yes	Yes	Yes	Yes
P value summary	ns	ns	ns	ns
**Kolmogorov-Smirnov test**				
KS distance	0.2338	0.1744	0.1676	0.2127
P value	>0.1000	>0.1000	>0.1000	>0.1000
Passed normality test (alpha=0.05)?	Yes	Yes	Yes	Yes
P value summary	ns	ns	ns	ns
**Number of values**	6	6	6	6

ANVA results / Multiple comparisons

Ordinary one-way ANOVA Multiple comparisons								
Number of families	1							
Number of comparisons per family	3							
Alpha	0.05							
**Dunnett's multiple comparisons test**	**Mean Diff.**	**95.00% CI of diff.**	**Significant?**	**Summary**	**Adjusted P Value**	**A-?**		
CTR vs. 10nM 1gg	-1464	-14247 to 11320	No	ns	0.9829	B	10nM 1gg	
CTR vs. 10nM 3gg	13470	686.2 to 26253	Yes	*	0.0377	C	10nM 3gg	
CTR vs. 10nM 7gg	13369	585.8 to 26152	Yes	*	0.0393	D	10nM 7gg	
**Test details**	**Mean 1**	**Mean 2**	**Mean Diff.**	**SE of diff.**	**n1**	**n2**	**q**	**DF**
CTR vs. 10nM 1gg	43084	44548	-1464	5032	6	6	0.2909	20
CTR vs. 10nM 3gg	43084	29615	13470	5032	6	6	2.677	20
CTR vs. 10nM 7gg	43084	29715	13369	5032	6	6	2.657	20

Statistics on metabolomics data – Reduced Glutathione

Normality and Lognormality Tests Tabular results	A CTR	B 10nM 1gg	C 10nM 3gg	D 10nM 7gg
**Shapiro-Wilk test**				
W	0.7923	0.8871	0.9805	0.9555
P value	0.0500	0.3032	0.9540	0.7847
Passed normality test (alpha=0.05)?	Yes	Yes	Yes	Yes
P value summary	ns	ns	ns	ns
**Kolmogorov-Smirnov test**				
KS distance	0.3273	0.2982	0.1772	0.1558
P value	0.0436	>0.1000	>0.1000	>0.1000
Passed normality test (alpha=0.05)?	No	Yes	Yes	Yes
P value summary	*	ns	ns	ns
**Number of values**	6	6	6	6

ANOVA results / Multiple comparisons

Ordinary one-way ANOVA Multiple comparisons								
Number of families	1							
Number of comparisons per family	3							
Alpha	0.05							
**Dunnett's multiple comparisons test**	**Mean Diff.**	**95.00% CI of diff.**	**Significant?**	**Summary**	**Adjusted P Value**	**A-?**		
CTR vs. 10nM 1gg	-560.3	-2363 to 1243	No	ns	0.7712	B	10nM 1gg	
CTR vs. 10nM 3gg	1641	-161.9 to 3444	No	ns	0.0791	C	10nM 3gg	
CTR vs. 10nM 7gg	1656	-146.5 to 3459	No	ns	0.0758	D	10nM 7gg	
**Test details**	**Mean 1**	**Mean 2**	**Mean Diff.**	**SE of diff.**	**n1**	**n2**	**q**	**DF**
CTR vs. 10nM 1gg	4431	4991	-560.3	709.6	6	6	0.7896	20
CTR vs. 10nM 3gg	4431	2790	1641	709.6	6	6	2.312	20
CTR vs. 10nM 7gg	4431	2774	1656	709.6	6	6	2.334	20

Statistics on metabolomics data – L-Glu

Normality and Lognormality Tests<br>Tabular results	A<br>CTR	B<br>10nM 1gg	C<br>10nM 3gg	D<br>10nM 7gg
**Shapiro-Wilk test**				
W	0.8995	0.9717	0.9452	0.9273
P value	0.3708	0.9037	0.7013	0.5595
Passed normality test (alpha=0.05)?	Yes	Yes	Yes	Yes
P value summary	ns	ns	ns	ns
**Kolmogorov-Smirnov test**				
KS distance	0.3023	0.1625	0.2258	0.2186
P value	0.0918	>0.1000	>0.1000	>0.1000
Passed normality test (alpha=0.05)?	Yes	Yes	Yes	Yes
P value summary	ns	ns	ns	ns
**Number of values**	6	6	6	6

ANOVA results | Multiple comparisons

Ordinary one-way ANOVA<br>Multiple comparisons								
Number of families	1							
Number of comparisons per family	3							
Alpha	0.05							
**Dunnett's multiple comparisons test**	**Mean Diff.**	**95.00% CI of diff.**	**Significant?**	**Summary**	**Adjusted P Value**	**A-?**		
CTR vs. 10nM 1gg	341.8	-984.3 to 1668	No	ns	0.8506	B	10nM 1gg	
CTR vs. 10nM 3gg	1835	509.1 to 3161	Yes	**	0.0060	C	10nM 3gg	
CTR vs. 10nM 7gg	2373	1047 to 3699	Yes	***	0.0006	D	10nM 7gg	
**Test details**	**Mean 1**	**Mean 2**	**Mean Diff.**	**SE of diff.**	**n1**	**n2**	**q**	**DF**
CTR vs. 10nM 1gg	3710	3368	341.8	522.0	6	6	0.6549	20
CTR vs. 10nM 3gg	3710	1875	1835	522.0	6	6	3.516	20
CTR vs. 10nM 7gg	3710	1337	2373	522.0	6	6	4.546	20

Statistics on metabolomics data – L-Gln

Normality and Lognormality Tests<br>Tabular results	A<br>CTR	B<br>10nM 1gg	C<br>10nM 3gg	D<br>10nM 7gg
**Shapiro-Wilk test**				
W	0.8971	0.8271	0.8574	0.9278
P value	0.3572	0.1016	0.1805	0.5636
Passed normality test (alpha=0.05)?	Yes	Yes	Yes	Yes
P value summary	ns	ns	ns	ns
**Kolmogorov-Smirnov test**				
KS distance	0.2466	0.2540	0.2977	0.2030
P value	>0.1000	>0.1000	>0.1000	>0.1000
Passed normality test (alpha=0.05)?	Yes	Yes	Yes	Yes
P value summary	ns	ns	ns	ns
**Number of values**	6	6	6	6

ANOVA results | Multiple comparisons

Ordinary one-way ANOVA<br>Multiple comparisons								
Number of families	1							
Number of comparisons per family	3							
Alpha	0.05							
**Dunnett's multiple comparisons test**	**Mean Diff.**	**95.00% CI of diff.**	**Significant?**	**Summary**	**Adjusted P Value**	**A-?**		
CTR vs. 10nM 1gg	-3077	-6603 to 448.3	No	ns	0.0952	B	10nM 1gg	
CTR vs. 10nM 3gg	4254	728.0 to 7779	Yes	*	0.0163	C	10nM 3gg	
CTR vs. 10nM 7gg	4259	733.6 to 7785	Yes	*	0.0162	D	10nM 7gg	
**Test details**	**Mean 1**	**Mean 2**	**Mean Diff.**	**SE of diff.**	**n1**	**n2**	**q**	**DF**
CTR vs. 10nM 1gg	6334	9412	-3077	1388	6	6	2.218	20
CTR vs. 10nM 3gg	6334	2081	4254	1388	6	6	3.065	20
CTR vs. 10nM 7gg	6334	2075	4259	1388	6	6	3.069	20

Statistics on metabolomics data – L-Cys

Normality and Lognormality Tests<br>Tabular results	A<br>CTR	B<br>10nM 1gg	C<br>10nM 3gg	D<br>10nM 7gg
**Shapiro-Wilk test**				
W	0.8271	0.8124	0.9068	0.9735
P value	0.1017	0.0758	0.4156	0.9153
Passed normality test (alpha=0.05)?	Yes	Yes	Yes	Yes
P value summary	ns	ns	ns	ns
**Kolmogorov-Smirnov test**				
KS distance	0.3563	0.2970	0.2306	0.1647
P value	0.0166	>0.1000	>0.1000	>0.1000
Passed normality test (alpha=0.05)?	No	Yes	Yes	Yes
P value summary	*	ns	ns	ns
**Number of values**	6	6	6	6

ANOVA results | Multiple comparisons

Ordinary one-way ANOVA<br>Multiple comparisons								
Number of families	1							
Number of comparisons per family	3							
Alpha	0.05							
**Dunnett's multiple comparisons test**	**Mean Diff.**	**95.00% CI of diff.**	**Significant?**	**Summary**	**Adjusted P Value**	**A-?**		
CTR vs. 10nM 1gg	-28.81	-51.10 to -6.527	Yes	*	0.0100	B	10nM 1gg	
CTR vs. 10nM 3gg	-5.077	-27.36 to 17.21	No	ns	0.8895	C	10nM 3gg	
CTR vs. 10nM 7gg	0.2178	-22.07 to 22.50	No	ns	>0.9999	D	10nM 7gg	
**Test details**	**Mean 1**	**Mean 2**	**Mean Diff.**	**SE of diff.**	**n1**	**n2**	**q**	**DF**
CTR vs. 10nM 1gg	45.28	74.09	-28.81	8.772	6	6	3.285	20
CTR vs. 10nM 3gg	45.28	50.35	-5.077	8.772	6	6	0.5788	20
CTR vs. 10nM 7gg	45.28	45.06	0.2178	8.772	6	6	0.02483	20

Statistics on metabolomics data – γGC

Normality and Lognormality Tests<br>Tabular results	A<br>CTR	B<br>10nM 1gg	C<br>10nM 3gg	D<br>10nM 7gg
**Shapiro-Wilk test**				
W	0.8492	0.9629	0.9439	0.9283
P value	0.1551	0.8416	0.6911	0.5667
Passed normality test (alpha=0.05)?	Yes	Yes	Yes	Yes
P value summary	ns	ns	ns	ns
**Kolmogorov-Smirnov test**				
KS distance	0.2990	0.1953	0.1789	0.1978
P value	>0.1000	>0.1000	>0.1000	>0.1000
Passed normality test (alpha=0.05)?	Yes	Yes	Yes	Yes
P value summary	ns	ns	ns	ns
**Number of values**	6	6	6	6

ANOVA results | Multiple comparisons

Ordinary one-way ANOVA<br>Multiple comparisons								
Number of families	1							
Number of comparisons per family	3							
Alpha	0.05							
**Dunnett's multiple comparisons test**	**Mean Diff.**	**95.00% CI of diff.**	**Significant?**	**Summary**	**Adjusted P Value**	**A-?**		
CTR vs. 10nM 1gg	107.3	22.00 to 192.7	Yes	*	0.0122	B	10nM 1gg	
CTR vs. 10nM 3gg	135.8	50.49 to 221.2	Yes	**	0.0018	C	10nM 3gg	
CTR vs. 10nM 7gg	98.80	13.45 to 184.1	Yes	*	0.0214	D	10nM 7gg	
**Test details**	**Mean 1**	**Mean 2**	**Mean Diff.**	**SE of diff.**	**n1**	**n2**	**q**	**DF**
CTR vs. 10nM 1gg	281.9	174.5	107.3	33.59	6	6	3.195	20
CTR vs. 10nM 3gg	281.9	146.1	135.8	33.59	6	6	4.044	20
CTR vs. 10nM 7gg	281.9	183.1	98.80	33.59	6	6	2.941	20

Statistics on metabolomics data – L-Ser

Normality and Lognormality Tests<br>Tabular results	A<br>CTR	B<br>10nM 1gg	C<br>10nM 3gg	D<br>10nM 7gg
**Shapiro-Wilk test**				
W	0.8985	0.9596	0.8769	0.9087
P value	0.3650	0.8167	0.2553	0.4282
Passed normality test (alpha=0.05)?	Yes	Yes	Yes	Yes
P value summary	ns	ns	ns	ns
**Kolmogorov-Smirnov test**				
KS distance	0.2227	0.2340	0.2318	0.2491
P value	>0.1000	>0.1000	>0.1000	>0.1000
Passed normality test (alpha=0.05)?	Yes	Yes	Yes	Yes
P value summary	ns	ns	ns	ns
**Number of values**	6	6	6	6

ANOVA results | Multiple comparisons

Ordinary one-way ANOVA<br>Multiple comparisons	
Number of families	1
Number of comparisons per family	3
Alpha	0.05

**Dunnett's multiple comparisons test**	**Mean Diff.**	**95.00% CI of diff.**	**Significant?**	**Summary**	**Adjusted P Value**	**A-?**		
CTR vs. 10nM 1gg	-514.7	-1216 to 186.6	No	ns	0.1822	B	10nM 1gg	
CTR vs. 10nM 3gg	836.4	135.2 to 1538	Yes	*	0.0176	C	10nM 3gg	
CTR vs. 10nM 7gg	860.5	159.2 to 1562	Yes	*	0.0145	D	10nM 7gg	
**Test details**	**Mean 1**	**Mean 2**	**Mean Diff.**	**SE of diff.**	**n1**	**n2**	**q**	**DF**
CTR vs. 10nM 1gg	1326	1840	-514.7	276.0	6	6	1.865	20
CTR vs. 10nM 3gg	1326	489.2	836.4	276.0	6	6	3.030	20
CTR vs. 10nM 7gg	1326	465.1	860.5	276.0	6	6	3.117	20

Statistics on metabolomics data – L-Gly

Normality and Lognormality Tests<br>Tabular results	A<br>CTR	B<br>10nM 1gg	C<br>10nM 3gg	D<br>10nM 7gg
**Shapiro-Wilk test**				
W	0.9707	0.9663	0.8404	0.8925
P value	0.8972	0.8665	0.1314	0.3315
Passed normality test (alpha=0.05)?	Yes	Yes	Yes	Yes
P value summary	ns	ns	ns	ns
**Kolmogorov-Smirnov test**				
KS distance	0.1369	0.2073	0.3259	0.2099
P value	>0.1000	>0.1000	0.0455	>0.1000
Passed normality test (alpha=0.05)?	Yes	Yes	No	Yes
P value summary	ns	ns	*	ns
**Number of values**	6	6	6	6

ANOVA results | Multiple comparisons

Ordinary one-way ANOVA<br>Multiple comparisons	
Number of families	1
Number of comparisons per family	3
Alpha	0.05

**Dunnett's multiple comparisons test**	**Mean Diff.**	**95.00% CI of diff.**	**Significant?**	**Summary**	**Adjusted P Value**	**A-?**		
CTR vs. 10nM 1gg	-100.9	-349.6 to 147.8	No	ns	0.6119	B	10nM 1gg	
CTR vs. 10nM 3gg	265.0	16.27 to 513.7	Yes	*	0.0354	C	10nM 3gg	
CTR vs. 10nM 7gg	273.8	25.14 to 522.5	Yes	*	0.0292	D	10nM 7gg	
**Test details**	**Mean 1**	**Mean 2**	**Mean Diff.**	**SE of diff.**	**n1**	**n2**	**q**	**DF**
CTR vs. 10nM 1gg	416.9	517.9	-100.9	97.89	6	6	1.031	20
CTR vs. 10nM 3gg	416.9	152.0	265.0	97.89	6	6	2.707	20
CTR vs. 10nM 7gg	416.9	143.1	273.8	97.89	6	6	2.797	20

Statistics on metabolomics data – L-Pro

Normality and Lognormality Tests<br>Tabular results	A<br>CTR	B<br>10nM 1d	C<br>10nM 3d	D<br>10nM 7d
**Shapiro-Wilk test**				
W	0.7946	0.8869	0.8033	0.9900
P value	0.0526	0.3021	0.0630	0.9892
Passed normality test (alpha=0.05)?	Yes	Yes	Yes	Yes
P value summary	ns	ns	ns	ns
**Kolmogorov-Smirnov test**				
KS distance	0.3217	0.2427	0.2739	0.1349
P value	0.0518	>0.1000	>0.1000	>0.1000
Passed normality test (alpha=0.05)?	Yes	Yes	Yes	Yes
P value summary	ns	ns	ns	ns
**Number of values**	6	6	6	6

ANOVA results — Multiple comparisons

Ordinary one-way ANOVA<br>Multiple comparisons								
Number of families	1							
Number of comparisons per family	3							
Alpha	0.05							
**Dunnett's multiple comparisons test**	**Mean Diff.**	**95.00% CI of diff.**	**Significant?**	**Summary**	**Adjusted P Value**	**A-?**		
CTR vs. 10nM 1d	2.571	-22.12 to 27.26	No	ns	0.9871	B	10nM 1d	
CTR vs. 10nM 3d	33.17	8.477 to 57.86	Yes	**	0.0075	C	10nM 3d	
CTR vs. 10nM 7d	31.75	7.055 to 56.43	Yes	*	0.0104	D	10nM 7d	
**Test details**	**Mean 1**	**Mean 2**	**Mean Diff.**	**SE of diff.**	**n1**	**n2**	**q**	**DF**
CTR vs. 10nM 1d	87.22	84.65	2.571	9.718	6	6	0.2646	20
CTR vs. 10nM 3d	87.22	54.06	33.17	9.718	6	6	3.413	20
CTR vs. 10nM 7d	87.22	55.48	31.75	9.718	6	6	3.267	20

Statistics on metabolomics data – L-Arg

Normality and Lognormality Tests<br>Tabular results	A<br>CTR	B<br>10nM 1d	C<br>10nM 3d	D<br>10nM 7d
**Shapiro-Wilk test**				
W	0.9631	0.9497	0.9604	0.9133
P value	0.8431	0.7377	0.8231	0.4581
Passed normality test (alpha=0.05)?	Yes	Yes	Yes	Yes
P value summary	ns	ns	ns	ns
**Kolmogorov-Smirnov test**				
KS distance	0.1760	0.2140	0.1963	0.1962
P value	>0.1000	>0.1000	>0.1000	>0.1000
Passed normality test (alpha=0.05)?	Yes	Yes	Yes	Yes
P value summary	ns	ns	ns	ns
**Number of values**	6	6	6	6

ANOVA results — Multiple comparisons

Ordinary one-way ANOVA<br>Multiple comparisons								
Number of families	1							
Number of comparisons per family	3							
Alpha	0.05							
**Dunnett's multiple comparisons test**	**Mean Diff.**	**95.00% CI of diff.**	**Significant?**	**Summary**	**Adjusted P Value**	**A-?**		
CTR vs. 10nM 1d	-36.11	-107.2 to 34.97	No	ns	0.4454	B	10nM 1d	
CTR vs. 10nM 3d	84.83	13.75 to 155.9	Yes	*	0.0176	C	10nM 3d	
CTR vs. 10nM 7d	87.34	16.25 to 158.4	Yes	*	0.0144	D	10nM 7d	
**Test details**	**Mean 1**	**Mean 2**	**Mean Diff.**	**SE of diff.**	**n1**	**n2**	**q**	**DF**
CTR vs. 10nM 1d	263.0	299.1	-36.11	27.98	6	6	1.291	20
CTR vs. 10nM 3d	263.0	178.2	84.83	27.98	6	6	3.032	20
CTR vs. 10nM 7d	263.0	175.7	87.34	27.98	6	6	3.122	20

Statistics on metabolomics data – Folate

Normality and Lognormality Tests<br>Tabular results	A<br>CTR	B<br>10nM 1gg	C<br>10nM 3gg	D<br>10nM 7gg
**Shapiro-Wilk test**				
W	0.8947	0.9210	0.9033	0.9461
P value	0.3435	0.5123	0.3939	0.7082
Passed normality test (alpha=0.05)?	Yes	Yes	Yes	Yes
P value summary	ns	ns	ns	ns
**Kolmogorov-Smirnov test**				
KS distance	0.2429	0.2099	0.2181	0.1594
P value	>0.1000	>0.1000	>0.1000	>0.1000
Passed normality test (alpha=0.05)?	Yes	Yes	Yes	Yes
P value summary	ns	ns	ns	ns
**Number of values**	6	6	6	6

ANOVA results | Multiple comparisons

Ordinary one-way ANOVA<br>Multiple comparisons								
Number of families	1							
Number of comparisons per family	3							
Alpha	0.05							
**Sidak's multiple comparisons test**	**Mean Diff.**	**95.00% CI of diff.**	**Significant?**	**Summary**	**Adjusted P Value**	**A-?**		
CTR vs. 10nM 1gg	297.9	120.3 to 475.5	Yes	***	0.0009	B	10nM 1gg	
CTR vs. 10nM 3gg	240.6	63.05 to 418.2	Yes	**	0.0063	C	10nM 3gg	
CTR vs. 10nM 7gg	425.3	247.7 to 602.9	Yes	****	<0.0001	D	10nM 7gg	
**Test details**	**Mean 1**	**Mean 2**	**Mean Diff.**	**SE of diff.**	**n1**	**n2**	**t**	**DF**
CTR vs. 10nM 1gg	883.3	585.4	297.9	68.18	6	6	4.369	20
CTR vs. 10nM 3gg	883.3	642.7	240.6	68.18	6	6	3.529	20
CTR vs. 10nM 7gg	883.3	458.0	425.3	68.18	6	6	6.238	20

Statistics on metabolomics data – 5,10-Methylene-THF

Normality and Lognormality Tests<br>Tabular results	A<br>CTR	B<br>10nM 1gg	C<br>10nM 3gg	D<br>10nM 7gg
**Shapiro-Wilk test**				
W	0.9036	0.8718	0.9819	0.9629
P value	0.3959	0.2334	0.9607	0.8420
Passed normality test (alpha=0.05)?	Yes	Yes	Yes	Yes
P value summary	ns	ns	ns	ns
**Kolmogorov-Smirnov test**				
KS distance	0.2063	0.2913	0.1506	0.1874
P value	>0.1000	>0.1000	>0.1000	>0.1000
Passed normality test (alpha=0.05)?	Yes	Yes	Yes	Yes
P value summary	ns	ns	ns	ns
**Number of values**	6	6	6	6

ANOVA results | Multiple comparisons

Ordinary one-way ANOVA<br>Multiple comparisons								
Number of families	1							
Number of comparisons per family	3							
Alpha	0.05							
**Dunnett's multiple comparisons test**	**Mean Diff.**	**95.00% CI of diff.**	**Significant?**	**Summary**	**Adjusted P Value**	**A-?**		
CTR vs. 10nM 1gg	0.4989	-20.71 to 21.71	No	ns	0.9999	B	10nM 1gg	
CTR vs. 10nM 3gg	0.000	-21.21 to 21.21	No	ns	>0.9999	C	10nM 3gg	
CTR vs. 10nM 7gg	-0.3333	-21.54 to 20.88	No	ns	>0.9999	D	10nM 7gg	
**Test details**	**Mean 1**	**Mean 2**	**Mean Diff.**	**SE of diff.**	**n1**	**n2**	**q**	**DF**
CTR vs. 10nM 1gg	48.67	48.17	0.4989	8.348	6	6	0.05976	20
CTR vs. 10nM 3gg	48.67	48.67	0.000	8.348	6	6	0.000	20
CTR vs. 10nM 7gg	48.67	49.00	-0.3333	8.348	6	6	0.03993	20

Statistics on metabolomics data – DMG

Normality and Lognormality Tests<br>Tabular results	A<br>CTR	B<br>10nM 1gg	C<br>10nM 3gg	D<br>10nM 7gg
**Shapiro-Wilk test**				
W	0.8969	0.8951	0.9592	0.8926
P value	0.3561	0.3460	0.8135	0.3321
Passed normality test (alpha=0.05)?	Yes	Yes	Yes	Yes
P value summary	ns	ns	ns	ns
**Kolmogorov-Smirnov test**				
KS distance	0.2154	0.2674	0.1889	0.2483
P value	>0.1000	>0.1000	>0.1000	>0.1000
Passed normality test (alpha=0.05)?	Yes	Yes	Yes	Yes
P value summary	ns	ns	ns	ns
**Number of values**	6	6	6	6

ANVOA results — Multiple comparisons

Ordinary one-way ANOVA<br>Multiple comparisons								
Number of families	1							
Number of comparisons per family	3							
Alpha	0.05							
**Dunnett's multiple comparisons test**	**Mean Diff.**	**95.00% CI of diff.**	**Significant?**	**Summary**	**Adjusted P Value**	**A-?**		
CTR vs. 10nM 1gg	-5.945	-84.85 to 72.96	No	ns	0.9949	B	10nM 1gg	
CTR vs. 10nM 3gg	99.67	20.76 to 178.6	Yes	*	0.0119	C	10nM 3gg	
CTR vs. 10nM 7gg	120.8	41.87 to 199.7	Yes	**	0.0025	D	10nM 7gg	
**Test details**	**Mean 1**	**Mean 2**	**Mean Diff.**	**SE of diff.**	**n1**	**n2**	**q**	**DF**
CTR vs. 10nM 1gg	203.9	209.8	-5.945	31.06	6	6	0.1914	20
CTR vs. 10nM 3gg	203.9	104.2	99.67	31.06	6	6	3.209	20
CTR vs. 10nM 7gg	203.9	83.11	120.8	31.06	6	6	3.889	20

Statistics on metabolomics data – L-Meth/Hcy

Normality and Lognormality Tests<br>Tabular results	A<br>CTR	B<br>10nM 1gg	C<br>10nM 3gg	D<br>10nM 7gg
**Shapiro-Wilk test**				
W	0.7996	0.8863	0.8923	0.9395
P value	0.0582	0.2993	0.3307	0.6548
Passed normality test (alpha=0.05)?	Yes	Yes	Yes	Yes
P value summary	ns	ns	ns	ns
**Kolmogorov-Smirnov test**				
KS distance	0.3314	0.2530	0.2146	0.2597
P value	0.0382	>0.1000	>0.1000	>0.1000
Passed normality test (alpha=0.05)?	No	Yes	Yes	Yes
P value summary	*	ns	ns	ns
**Number of values**	6	6	6	6

ANOVA results — Multiple comparisons

Ordinary one-way ANOVA<br>Multiple comparisons								
Number of families	1							
Number of comparisons per family	3							
Alpha	0.05							
**Dunnett's multiple comparisons test**	**Mean Diff.**	**95.00% CI of diff.**	**Significant?**	**Summary**	**Adjusted P Value**	**A-?**		
CTR vs. 10nM 1gg	2.763	-7.261 to 12.79	No	ns	0.8252	B	10nM 1gg	
CTR vs. 10nM 3gg	1.811	-8.213 to 11.83	No	ns	0.9394	C	10nM 3gg	
CTR vs. 10nM 7gg	11.52	1.492 to 21.54	Yes	*	0.0225	D	10nM 7gg	
**Test details**	**Mean 1**	**Mean 2**	**Mean Diff.**	**SE of diff.**	**n1**	**n2**	**q**	**DF**
CTR vs. 10nM 1gg	27.69	24.93	2.763	3.945	6	6	0.7003	20
CTR vs. 10nM 3gg	27.69	25.88	1.811	3.945	6	6	0.4590	20
CTR vs. 10nM 7gg	27.69	16.18	11.52	3.945	6	6	2.919	20

Statistics on metabolomics data – L-Meth

Normality and Lognormality Tests<br>Tabular results	A<br>CTR	B<br>Rot 1d	C<br>Rot 3d	D<br>Rot 7d
**Shapiro-Wilk test**				
W	0.9623	0.9708	0.9367	0.9468
P value	0.8376	0.8979	0.6324	0.7140
Passed normality test (alpha=0.05)?	Yes	Yes	Yes	Yes
P value summary	ns	ns	ns	ns
**Kolmogorov-Smirnov test**				
KS distance	0.1530	0.1667	0.2496	0.1665
P value	>0.1000	>0.1000	>0.1000	>0.1000
Passed normality test (alpha=0.05)?	Yes	Yes	Yes	Yes
P value summary	ns	ns	ns	ns
**Number of values**	6	6	6	6

ANOVA results × Multiple comparisons

Ordinary one-way ANOVA<br>Multiple comparisons								
Number of families	1							
Number of comparisons per family	3							
Alpha	0.05							
**Dunnett's multiple comparisons test**	**Mean Diff.**	**95.00% CI of diff.**	**Significant?**	**Summary**	**Adjusted P Value**	**A-?**		
CTR vs. Rot 1d	218.7	-47.07 to 484.5	No	ns	0.1210	B	Rot 1d	
CTR vs. Rot 3d	446.2	180.4 to 712.0	Yes	**	0.0011	C	Rot 3d	
CTR vs. Rot 7d	641.3	375.5 to 907.1	Yes	****	<0.0001	D	Rot 7d	
**Test details**	**Mean 1**	**Mean 2**	**Mean Diff.**	**SE of diff.**	**n1**	**n2**	**q**	**DF**
CTR vs. Rot 1d	1041	821.9	218.7	104.6	6	6	2.091	20
CTR vs. Rot 3d	1041	594.4	446.2	104.6	6	6	4.265	20
CTR vs. Rot 7d	1041	399.3	641.3	104.6	6	6	6.129	20

Statistics on metabolomics data – Homocysteine

Normality and Lognormality Tests<br>Tabular results	A<br>CTR	B<br>Rot 1d	C<br>Rot 3d	D<br>Rot 7d
**Shapiro-Wilk test**				
W	0.8016	0.9051	0.9531	0.9734
P value	0.0607	0.4052	0.7650	0.9147
Passed normality test (alpha=0.05)?	Yes	Yes	Yes	Yes
P value summary	ns	ns	ns	ns
**Kolmogorov-Smirnov test**				
KS distance	0.3148	0.2574	0.1753	0.1849
P value	0.0638	>0.1000	>0.1000	>0.1000
Passed normality test (alpha=0.05)?	Yes	Yes	Yes	Yes
P value summary	ns	ns	ns	ns
**Number of values**	6	6	6	6

ANOVA results × Multiple comparisons

Ordinary one-way ANOVA<br>Multiple comparisons								
Number of families	1							
Number of comparisons per family	3							
Alpha	0.05							
**Dunnett's multiple comparisons test**	**Mean Diff.**	**95.00% CI of diff.**	**Significant?**	**Summary**	**Adjusted P Value**	**A-?**		
CTR vs. Rot 1d	4.206	-6.774 to 15.18	No	ns	0.6507	B	Rot 1d	
CTR vs. Rot 3d	10.25	-0.7293 to 21.23	No	ns	0.0703	C	Rot 3d	
CTR vs. Rot 7d	7.723	-3.256 to 18.70	No	ns	0.2082	D	Rot 7d	
**Test details**	**Mean 1**	**Mean 2**	**Mean Diff.**	**SE of diff.**	**n1**	**n2**	**q**	**DF**
CTR vs. Rot 1d	40.40	36.19	4.206	4.322	6	6	0.9731	20
CTR vs. Rot 3d	40.40	30.15	10.25	4.322	6	6	2.372	20
CTR vs. Rot 7d	40.40	32.68	7.723	4.322	6	6	1.787	20

Statistics on metabolomics data – Glucosamine-6-phosphate (GlcN6P)

Normality and Lognormality Tests<br>Tabular results	A<br>CTR	B<br>10nM 1gg	C<br>10nM 3gg	D<br>10nM 7gg
**Shapiro-Wilk test**				
W	0.8879	0.9541	0.9794	0.8878
P value	0.3073	0.7736	0.9485	0.3070
Passed normality test (alpha=0.05)?	Yes	Yes	Yes	Yes
P value summary	ns	ns	ns	ns
**Kolmogorov-Smirnov test**				
KS distance	0.2265	0.1773	0.1781	0.2622
P value	>0.1000	>0.1000	>0.1000	>0.1000
Passed normality test (alpha=0.05)?	Yes	Yes	Yes	Yes
P value summary	ns	ns	ns	ns
**Number of values**	6	6	6	6

ANOVA results | Multiple comparisons

Ordinary one-way ANOVA<br>Multiple comparisons								
Number of families	1							
Number of comparisons per family	3							
Alpha	0.05							
**Dunnett's multiple comparisons test**	**Mean Diff.**	**95.00% CI of diff.**	**Significant?**	**Summary**	**Adjusted P Value**	**A-?**		
CTR vs. 10nM 1gg	0.1822	-102.2 to 102.5	No	ns	>0.9999	B	10nM 1gg	
CTR vs. 10nM 3gg	128.1	25.78 to 230.5	Yes	*	0.0126	C	10nM 3gg	
CTR vs. 10nM 7gg	124.8	22.44 to 227.1	Yes	*	0.0152	D	10nM 7gg	
**Test details**	**Mean 1**	**Mean 2**	**Mean Diff.**	**SE of diff.**	**n1**	**n2**	**q**	**DF**
CTR vs. 10nM 1gg	255.9	255.8	0.1822	40.28	6	6	0.004523	20
CTR vs. 10nM 3gg	255.9	127.8	128.1	40.28	6	6	3.181	20
CTR vs. 10nM 7gg	255.9	131.2	124.8	40.28	6	6	3.098	20

Statistics on metabolomics data – N-acetylglucosamine 6-phosphate (GlcNAc-6P)

Normality and Lognormality Tests<br>Tabular results	A<br>CTR	B<br>10nM 1gg	C<br>10nM 3gg	D<br>10nM 7gg
**Shapiro-Wilk test**				
W	0.8092	0.9320	0.9347	0.8352
P value	0.0710	0.5957	0.6168	0.1189
Passed normality test (alpha=0.05)?	Yes	Yes	Yes	Yes
P value summary	ns	ns	ns	ns
**Kolmogorov-Smirnov test**				
KS distance	0.3009	0.1863	0.2091	0.3193
P value	0.0957	>0.1000	>0.1000	0.0557
Passed normality test (alpha=0.05)?	Yes	Yes	Yes	Yes
P value summary	ns	ns	ns	ns
**Number of values**	6	6	6	6

ANOVA results | Multiple comparisons

Ordinary one-way ANOVA<br>Multiple comparisons								
Number of families	1							
Number of comparisons per family	3							
Alpha	0.05							
**Dunnett's multiple comparisons test**	**Mean Diff.**	**95.00% CI of diff.**	**Significant?**	**Summary**	**Adjusted P Value**	**A-?**		
CTR vs. 10nM 1gg	-247.6	-631.3 to 136.1	No	ns	0.2659	B	10nM 1gg	
CTR vs. 10nM 3gg	469.0	85.29 to 852.7	Yes	*	0.0149	C	10nM 3gg	
CTR vs. 10nM 7gg	385.4	1.693 to 769.1	Yes	*	0.0489	D	10nM 7gg	
**Test details**	**Mean 1**	**Mean 2**	**Mean Diff.**	**SE of diff.**	**n1**	**n2**	**q**	**DF**
CTR vs. 10nM 1gg	1189	1437	-247.6	151.0	6	6	1.639	20
CTR vs. 10nM 3gg	1189	720.0	469.0	151.0	6	6	3.105	20
CTR vs. 10nM 7gg	1189	803.6	385.4	151.0	6	6	2.552	20

Statistics on metabolomics data –Uridine diphosphate N-acetylglucosamine (UDP-GlcNAc)

Normality and Lognormality Tests<br>Tabular results	A<br>CTR	B<br>10nM 1gg	C<br>10nM 3gg	D<br>10nM 7gg
**Shapiro-Wilk test**				
W	0.9362	0.8580	0.8991	0.8994
P value	0.6286	0.1825	0.3687	0.3701
Passed normality test (alpha=0.05)?	Yes	Yes	Yes	Yes
P value summary	ns	ns	ns	ns
**Kolmogorov-Smirnov test**				
KS distance	0.1815	0.2749	0.2444	0.1874
P value	>0.1000	>0.1000	>0.1000	>0.1000
Passed normality test (alpha=0.05)?	Yes	Yes	Yes	Yes
P value summary	ns	ns	ns	ns
**Number of values**	6	6	6	6

Ordinary one-way ANOVA<br>Multiple comparisons								
Number of families	1							
Number of comparisons per family	3							
Alpha	0.05							
**Dunnett's multiple comparisons test**	**Mean Diff.**	**95.00% CI of diff.**	**Significant?**	**Summary**	**Adjusted P Value**	**A-?**		
CTR vs. 10nM 1gg	4057	-589.4 to 8703	No	ns	0.0951	B	10nM 1gg	
CTR vs. 10nM 3gg	4239	-407.3 to 8885	No	ns	0.0783	C	10nM 3gg	
CTR vs. 10nM 7gg	5519	873.0 to 10165	Yes	*	0.0181	D	10nM 7gg	
**Test details**	**Mean 1**	**Mean 2**	**Mean Diff.**	**SE of diff.**	**n1**	**n2**	**q**	**DF**
CTR vs. 10nM 1gg	15263	11206	4057	1829	6	6	2.218	20
CTR vs. 10nM 3gg	15263	11024	4239	1829	6	6	2.318	20
CTR vs. 10nM 7gg	15263	9744	5519	1829	6	6	3.018	20

Statistics on metabolomics data – N-acetylglucosamine 6-phosphate (GlcNAc-6P)

Normality and Lognormality Tests<br>Tabular results	A<br>CTR	B<br>10nM 1gg	C<br>10nM 3gg	D<br>10nM 7gg
**Shapiro-Wilk test**				
W	0.8959	0.9518	0.8800	0.9192
P value	0.3504	0.7548	0.2692	0.4999
Passed normality test (alpha=0.05)?	Yes	Yes	Yes	Yes
P value summary	ns	ns	ns	ns
**Kolmogorov-Smirnov test**				
KS distance	0.1803	0.1630	0.2195	0.2022
P value	>0.1000	>0.1000	>0.1000	>0.1000
Passed normality test (alpha=0.05)?	Yes	Yes	Yes	Yes
P value summary	ns	ns	ns	ns
**Number of values**	6	6	6	6

Ordina **ANOVA results** IOVA<br>Multiple comparisons								
Number of families	1							
Number of comparisons per family	3							
Alpha	0.05							
**Dunnett's multiple comparisons test**	**Mean Diff.**	**95.00% CI of diff.**	**Significant?**	**Summary**	**Adjusted P Value**	**A-?**		
CTR vs. 10nM 1gg	-0.09600	-0.4052 to 0.2132	No	ns	0.7717	B	10nM 1gg	
CTR vs. 10nM 3gg	0.2528	-0.05633 to 0.5620	No	ns	0.1240	C	10nM 3gg	
CTR vs. 10nM 7gg	0.3262	0.01700 to 0.6353	Yes	*	0.0374	D	10nM 7gg	
**Test details**	**Mean 1**	**Mean 2**	**Mean Diff.**	**SE of diff.**	**n1**	**n2**	**q**	**DF**
CTR vs. 10nM 1gg	0.7595	0.8555	-0.09600	0.1217	6	6	0.7889	20
CTR vs. 10nM 3gg	0.7595	0.5067	0.2528	0.1217	6	6	2.078	20
CTR vs. 10nM 7gg	0.7595	0.4333	0.3262	0.1217	6	6	2.680	20

Statistics on mitochondrial proteins –P(Ser 616)-Drp1

Normality and Lognormality Tests<br>Tabular results	A<br>CTR	B<br>200nM 3h	C<br>400nM 3h	D<br>800nM 3h	E<br>200nM 6h	F<br>400nM 6h	G<br>800nM 6h	H<br>200nM 24h	I<br>400nM 24h	J<br>800nM 24h
**Shapiro-Wilk test**										
W	0.9564	0.8818	0.9562	0.9133	0.9732	0.9357	0.9831	0.8458	0.9085	0.8085
P value	0.7830	0.3176	0.7816	0.4877	0.8954	0.6357	0.9505	0.1816	0.4589	0.0948
Passed normality test (alpha=0.05)?	Yes	Yes	Yes	Yes	Yes	Yes	Yes	Yes	Yes	Yes
P value summary	ns	ns	ns	ns	ns	ns	ns	ns	ns	ns
**Kolmogorov-Smirnov test**										
KS distance	0.1732	0.2682	0.1711	0.2754	0.1535	0.2428	0.1759	0.2771	0.2747	0.3259
P value	>0.1000	>0.1000	>0.1000	>0.1000	>0.1000	>0.1000	>0.1000	>0.1000	>0.1000	0.0888
Passed normality test (alpha=0.05)?	Yes	Yes	Yes	Yes	Yes	Yes	Yes	Yes	Yes	Yes
P value summary	ns	ns	ns	ns	ns	ns	ns	ns	ns	ns
**Number of values**	5	5	5	5	5	5	5	5	5	5

ANOVA results | Multiple comparisons

Ordinary one-way ANOVA<br>Multiple comparisons								
Number of families	1							
Number of comparisons per family	9							
Alpha	0.05							
**Dunnett's multiple comparisons test**	**Mean Diff.**	**95.00% CI of diff.**	**Significant?**	**Summary**	**Adjusted P Value**	**A-?**		
CTR vs. 200nM 3h	-7.000	-33.41 to 19.41	No	ns	0.9810	B	200nM 3h	
CTR vs. 400nM 3h	20.98	-5.432 to 47.40	No	ns	0.1755	C	400nM 3h	
CTR vs. 800nM 3h	31.14	4.730 to 57.56	Yes	*	0.0141	D	800nM 3h	
CTR vs. 200nM 6h	11.82	-14.59 to 38.24	No	ns	0.7524	E	200nM 6h	
CTR vs. 400nM 6h	31.10	4.688 to 57.52	Yes	*	0.0143	F	400nM 6h	
CTR vs. 800nM 6h	59.66	33.25 to 86.07	Yes	****	<0.0001	G	800nM 6h	
CTR vs. 200nM 24h	35.00	8.588 to 61.42	Yes	**	0.0046	H	200nM 24h	
CTR vs. 400nM 24h	70.85	44.44 to 97.26	Yes	****	<0.0001	I	400nM 24h	
CTR vs. 800nM 24h	83.60	57.18 to 110.0	Yes	****	<0.0001	J	800nM 24h	
**Test details**	**Mean 1**	**Mean 2**	**Mean Diff.**	**SE of diff.**	**n1**	**n2**	**q**	**DF**
CTR vs. 200nM 3h	100.0	107.0	-7.000	9.394	5	5	0.7452	40
CTR vs. 400nM 3h	100.0	79.02	20.98	9.394	5	5	2.234	40
CTR vs. 800nM 3h	100.0	68.86	31.14	9.394	5	5	3.315	40
CTR vs. 200nM 6h	100.0	88.18	11.82	9.394	5	5	1.259	40
CTR vs. 400nM 6h	100.0	68.90	31.10	9.394	5	5	3.311	40
CTR vs. 800nM 6h	100.0	40.34	59.66	9.394	5	5	6.351	40
CTR vs. 200nM 24h	100.0	65.00	35.00	9.394	5	5	3.726	40
CTR vs. 400nM 24h	100.0	29.15	70.85	9.394	5	5	7.542	40
CTR vs. 800nM 24h	100.0	16.40	83.60	9.394	5	5	8.899	40

Statistics on mitochondrial proteins –P(Ser 637)-Drp1

Normality and Lognormality Tests<br>Tabular results	A<br>CTR	B<br>800nM 3h	C<br>200nM 6h	D<br>400nM 6h
**Shapiro-Wilk test**				
W	0.8972	0.9667	0.8464	0.8620
P value	0.3946	0.8535	0.1833	0.2355
Passed normality test (alpha=0.05)?	Yes	Yes	Yes	Yes
P value summary	ns	ns	ns	ns
**Kolmogorov-Smirnov test**				
KS distance	0.2115	0.1638	0.2881	0.2267
P value	>0.1000	>0.1000	>0.1000	>0.1000
Passed normality test (alpha=0.05)?	Yes	Yes	Yes	Yes
P value summary	ns	ns	ns	ns
**Number of values**	5	5	5	5

ANVOA results — Multiple comparisons

Ordinary one-way ANOVA<br>Multiple comparisons								
Number of families	1							
Number of comparisons per family	3							
Alpha	0.05							
**Dunnett's multiple comparisons test**	**Mean Diff.**	**95.00% CI of diff.**	**Significant?**	**Summary**	**Adjusted P Value**	**A-?**		
CTR vs. 800nM 3h	-31.47	-54.18 to -8.769	Yes	**	0.0066	B	800nM 3h	
CTR vs. 200nM 6h	-24.95	-47.66 to -2.250	Yes	*	0.0301	C	200nM 6h	
CTR vs. 400nM 6h	-27.13	-49.84 to -4.430	Yes	*	0.0182	D	400nM 6h	
**Test details**	**Mean 1**	**Mean 2**	**Mean Diff.**	**SE of diff.**	**n1**	**n2**	**q**	**DF**
CTR vs. 800nM 3h	100.0	131.5	-31.47	8.757	5	5	3.594	16
CTR vs. 200nM 6h	100.0	125.0	-24.95	8.757	5	5	2.849	16
CTR vs. 400nM 6h	100.0	127.1	-27.13	8.757	5	5	3.098	16

Statistics on mitochondrial proteins – total Opa1

Normality and Lognormality Tests<br>Tabular results	A<br>CTR	B<br>200nM 3h	C<br>400nM 3h	D<br>800nM 3h	E<br>200nM 6h	F<br>400nM 6h	G<br>800nM 6h	H<br>200nM 24h	I<br>400nM 24h	J<br>800nM 24h
**Shapiro-Wilk test**										
W	0.9999	0.7752	0.8232	0.9026	0.8967	0.9818	0.9495	0.8386	0.7949	0.8457
P value	>0.9999	0.0501	0.1236	0.4244	0.3921	0.9442	0.7339	0.1610	0.0736	0.1812
Passed normality test (alpha=0.05)?	Yes	Yes	Yes	Yes	Yes	Yes	Yes	Yes	Yes	Yes
P value summary	ns	ns	ns	ns	ns	ns	ns	ns	ns	ns
**Kolmogorov-Smirnov test**										
KS distance	0.1097	0.2700	0.3113	0.2766	0.2500	0.1517	0.1878	0.3036	0.3310	0.3091
P value	>0.1000	>0.1000	>0.1000	>0.1000	>0.1000	>0.1000	>0.1000	>0.1000	0.0771	>0.1000
Passed normality test (alpha=0.05)?	Yes	Yes	Yes	Yes	Yes	Yes	Yes	Yes	Yes	Yes
P value summary	ns	ns	ns	ns	ns	ns	ns	ns	ns	ns
**Number of values**	5	5	5	5	5	5	5	5	5	5

Multiple comparisons

Ordinary one-way ANOVA<br>Multiple comparisons							
Number of families	1						
Number of comparisons per family	9						
Alpha	0.05						
**Dunnett's multiple comparisons test**	**Mean Diff.**	**95.00% CI of diff.**	**Significant?**	**Summary**	**Adjusted P Value**	**A-?**	
CTR vs. 200nM 3h	-9.400	-33.90 to 15.10	No	ns	0.8643	B	200nM 3h
CTR vs. 400nM 3h	-14.08	-38.58 to 10.42	No	ns	0.4971	C	400nM 3h
CTR vs. 800nM 3h	2.378	-22.12 to 26.87	No	ns	0.9996	D	800nM 3h
CTR vs. 200nM 6h	8.414	-16.08 to 32.91	No	ns	0.9186	E	200nM 6h
CTR vs. 400nM 6h	58.94	34.44 to 83.44	Yes	****	<0.0001	F	400nM 6h
CTR vs. 800nM 6h	49.04	24.55 to 73.54	Yes	****	<0.0001	G	800nM 6h
CTR vs. 200nM 24h	14.13	-10.36 to 38.63	No	ns	0.4929	H	200nM 24h
CTR vs. 400nM 24h	19.95	-4.550 to 44.44	No	ns	0.1571	I	400nM 24h
CTR vs. 800nM 24h	44.20	19.70 to 68.70	Yes	****	<0.0001	J	800nM 24h
**Test details**	**Mean 1**	**Mean 2**	**Mean Diff.**	**SE of diff.**	**n1**	**n2**	**q**	**DF**
CTR vs. 200nM 3h	100.0	109.4	-9.400	8.711	5	5	1.079	40
CTR vs. 400nM 3h	100.0	114.1	-14.08	8.711	5	5	1.616	40
CTR vs. 800nM 3h	100.0	97.62	2.378	8.711	5	5	0.2730	40
CTR vs. 200nM 6h	100.0	91.59	8.414	8.711	5	5	0.9658	40
CTR vs. 400nM 6h	100.0	41.06	58.94	8.711	5	5	6.766	40
CTR vs. 800nM 6h	100.0	50.96	49.04	8.711	5	5	5.630	40
CTR vs. 200nM 24h	100.0	85.87	14.13	8.711	5	5	1.622	40
CTR vs. 400nM 24h	100.0	80.05	19.95	8.711	5	5	2.290	40
CTR vs. 800nM 24h	100.0	55.80	44.20	8.711	5	5	5.074	40

Statistics on mitochondrial proteins – 100 kDa Opa1 isoform

Tabular results

Normality and Lognormality Tests<br>Tabular results	A<br>CTR	B<br>200nM 3h	C<br>400nM 3h	D<br>800nM 3h	E<br>200nM 6h	F<br>400nM 6h	G<br>800nM 6h	H<br>200nM 24h	I<br>400nM 24h	J<br>800nM 24h
13										
14 **Shapiro-Wilk test**										
15 W	0.9645	0.7752	0.9857	0.9400	0.9576	0.7666	0.8188	0.8273	0.7363	0.8788
16 P value	0.8389	0.0501	0.9625	0.6661	0.7915	0.0421	0.1143	0.1327	0.0221	0.3040
17 Passed normality test (alpha=0.05)?	Yes	Yes	Yes	Yes	Yes	No	Yes	Yes	No	Yes
18 P value summary	ns	ns	ns	ns	ns	*	ns	ns	*	ns
19										
20 **Kolmogorov-Smirnov test**										
21 KS distance	0.1671	0.2700	0.1580	0.2057	0.2097	0.3303	0.3247	0.2795	0.4031	0.2255
22 P value	>0.1000	>0.1000	>0.1000	>0.1000	>0.1000	0.0786	0.0917	>0.1000	0.0079	>0.1000
23 Passed normality test (alpha=0.05)?	Yes	Yes	Yes	Yes	Yes	Yes	Yes	Yes	No	Yes
24 P value summary	ns	ns	ns	ns	ns	ns	ns	ns	**	ns
25										
26 **Number of values**	5	5	5	5	5	5	5	5	5	5
27										

ANOVA results | Multiple comparisons

Ordinary one-way ANOVA<br>Multiple comparisons								
1 Number of families	1							
2 Number of comparisons per family	9							
3 Alpha	0.05							
4								
5 **Dunnett's multiple comparisons test**	**Mean Diff.**	**95.00% CI of diff.**	**Significant?**	**Summary**	**Adjusted P Value**	**A-?**		
6 CTR vs. 200nM 3h	-9.380	-46.71 to 27.95	No	ns	0.9868	B	200nM 3h	
7 CTR vs. 400nM 3h	-23.20	-60.53 to 14.13	No	ns	0.4107	C	400nM 3h	
8 CTR vs. 800nM 3h	-19.91	-57.24 to 17.42	No	ns	0.5793	D	800nM 3h	
9 CTR vs. 200nM 6h	13.69	-23.64 to 51.02	No	ns	0.8890	E	200nM 6h	
10 CTR vs. 400nM 6h	56.49	19.16 to 93.82	Yes	***	0.0010	F	400nM 6h	
11 CTR vs. 800nM 6h	52.84	15.51 to 90.17	Yes	**	0.0022	G	800nM 6h	
12 CTR vs. 200nM 24h	6.076	-31.26 to 43.41	No	ns	0.9978	H	200nM 24h	
13 CTR vs. 400nM 24h	38.53	1.196 to 75.86	Yes	*	0.0403	I	400nM 24h	
14 CTR vs. 800nM 24h	37.24	-0.09247 to 74.57	No	ns	0.0508	J	800nM 24h	
15								
16 **Test details**	**Mean 1**	**Mean 2**	**Mean Diff.**	**SE of diff.**	**n1**	**n2**	**q**	**DF**
17 CTR vs. 200nM 3h	100.0	109.4	-9.380	13.28	5	5	0.7065	40
18 CTR vs. 400nM 3h	100.0	123.2	-23.20	13.28	5	5	1.748	40
19 CTR vs. 800nM 3h	100.0	119.9	-19.91	13.28	5	5	1.500	40
20 CTR vs. 200nM 6h	100.0	86.33	13.69	13.28	5	5	1.031	40
21 CTR vs. 400nM 6h	100.0	43.53	56.49	13.28	5	5	4.255	40
22 CTR vs. 800nM 6h	100.0	47.18	52.84	13.28	5	5	3.980	40
23 CTR vs. 200nM 24h	100.0	93.94	6.076	13.28	5	5	0.4576	40
24 CTR vs. 400nM 24h	100.0	61.49	38.53	13.28	5	5	2.902	40
25 CTR vs. 800nM 24h	100.0	62.78	37.24	13.28	5	5	2.805	40

Statistics on mitochondrial proteins – Mfn2

Normality and Lognormality Tests<br>Tabular results	D<br>800nM 3h	E<br>200nM 6h	F<br>400nM 6h	G<br>800nM 6h	H<br>200nM 24h	I<br>400nM 24h	J<br>800nM 24h
**Shapiro-Wilk test**							
W	0.9205	0.9733	0.8979	0.8454	0.8256	0.8435	0.9338
P value	0.5329	0.8962	0.3984	0.1805	0.1289	0.1748	0.6228
Passed normality test (alpha=0.05)?	Yes	Yes	Yes	Yes	Yes	Yes	Yes
P value summary	ns	ns	ns	ns	ns	ns	ns
**Kolmogorov-Smirnov test**							
KS distance	0.2089	0.1870	0.2089	0.2602	0.3574	0.2675	0.2722
P value	>0.1000	>0.1000	>0.1000	>0.1000	0.0357	>0.1000	>0.1000
Passed normality test (alpha=0.05)?	Yes	Yes	Yes	Yes	No	Yes	Yes
P value summary	ns	ns	ns	ns	*	ns	ns
**Number of values**	5	5	5	5	5	5	5

ANVOA results | Multiple comparisons

Ordinary one-way ANOVA<br>Multiple comparisons							
Number of families	1						
Number of comparisons per family	9						
Alpha	0.05						
**Dunnett's multiple comparisons test**	**Mean Diff.**	**95.00% CI of diff.**	**Significant?**	**Summary**	**Adjusted P Value**	**A-?**	
CTR vs. 200nM 3h	3.914	-13.88 to 21.71	No	ns	0.9930	B	200nM 3h
CTR vs. 400nM 3h	9.372	-8.421 to 27.17	No	ns	0.5928	C	400nM 3h
CTR vs. 800nM 3h	16.96	-0.8331 to 34.75	No	ns	0.0679	D	800nM 3h
CTR vs. 200nM 6h	23.37	5.579 to 41.17	Yes	**	0.0050	E	200nM 6h
CTR vs. 400nM 6h	33.63	15.83 to 51.42	Yes	****	<0.0001	F	400nM 6h
CTR vs. 800nM 6h	49.30	31.50 to 67.09	Yes	****	<0.0001	G	800nM 6h
CTR vs. 200nM 24h	22.44	4.647 to 40.23	Yes	**	0.0076	H	200nM 24h
CTR vs. 400nM 24h	34.28	16.49 to 52.08	Yes	****	<0.0001	I	400nM 24h
CTR vs. 800nM 24h	39.68	21.88 to 57.47	Yes	****	<0.0001	J	800nM 24h

**Test details**	**Mean 1**	**Mean 2**	**Mean Diff.**	**SE of diff.**	**n1**	**n2**	**q**	**DF**
CTR vs. 200nM 3h	100.0	96.09	3.914	6.328	5	5	0.6185	40
CTR vs. 400nM 3h	100.0	90.63	9.372	6.328	5	5	1.481	40
CTR vs. 800nM 3h	100.0	83.04	16.96	6.328	5	5	2.680	40
CTR vs. 200nM 6h	100.0	76.63	23.37	6.328	5	5	3.694	40
CTR vs. 400nM 6h	100.0	66.37	33.63	6.328	5	5	5.314	40
CTR vs. 800nM 6h	100.0	50.70	49.30	6.328	5	5	7.791	40
CTR vs. 200nM 24h	100.0	77.56	22.44	6.328	5	5	3.546	40
CTR vs. 400nM 24h	100.0	65.72	34.28	6.328	5	5	5.418	40
CTR vs. 800nM 24h	100.0	60.32	39.68	6.328	5	5	6.270	40

Statistics on mitochondrial proteins –p62

Normality and Lognormality Tests<br>Tabular results	A<br>CTR	B<br>Rot 200nM	C<br>Rot 400nM	D<br>Rot 800nM
**Shapiro-Wilk test**				
W	0.9801	0.9138	0.8263	0.8678
P value	0.9350	0.4908	0.1304	0.2576
Passed normality test (alpha=0.05)?	Yes	Yes	Yes	Yes
P value summary	ns	ns	ns	ns
**Kolmogorov-Smirnov test**				
KS distance	0.1471	0.2006	0.3639	0.3112
P value	>0.1000	>0.1000	0.0292	>0.1000
Passed normality test (alpha=0.05)?	Yes	Yes	No	Yes
P value summary	ns	ns	*	ns
**Number of values**	5	5	5	5

ANVOA results | Multiple comparisons

Ordinary one-way ANOVA<br>Multiple comparisons								
**Dunnett's multiple comparisc**	**Mean Diff.**	**95.00% CI of diff.**	**Significant?**	**Summary**	**Adjusted P Value**	**A-?**		
CTR vs. Rot 200nM	-32.64	-51.43 to -13.85	Yes	**	0.0010	B	Rot 200nM	
CTR vs. Rot 400nM	-26.02	-44.81 to -7.226	Yes	**	0.0066	C	Rot 400nM	
CTR vs. Rot 800nM	-22.45	-41.24 to -3.664	Yes	*	0.0182	D	Rot 800nM	
**Test details**	**Mean 1**	**Mean 2**	**Mean Diff.**	**SE of diff.**	**n1**	**n2**	**q**	**DF**
CTR vs. Rot 200nM	100.0	132.6	-32.64	7.248	5	5	4.503	16
CTR vs. Rot 400nM	100.0	126.0	-26.02	7.248	5	5	3.590	16
CTR vs. Rot 800nM	100.0	122.5	-22.45	7.248	5	5	3.098	16

Statistics immunocytochemistry - P-Drp1/CoxIV colocalization – Pearson's coefficient of correlation (*r*) (% of CTR)

Tabular results

Normality and Lognormality Tests (Tabular results)	A: CTR	B: Rot 3h	C: Rot 6h	D
13				
14 **Shapiro-Wilk test**				
15 W	0.8743	0.9330	0.9389	
16 P value	0.2843	0.6167	0.6582	
17 Passed normality test (alpha=0.05)?	Yes	Yes	Yes	
18 P value summary	ns	ns	ns	
19				
20 **Kolmogorov-Smirnov test**				
21 KS distance	0.2874	0.2291	0.1863	
22 P value	>0.1000	>0.1000	>0.1000	
23 Passed normality test (alpha=0.05)?	Yes	Yes	Yes	
24 P value summary	ns	ns	ns	
25				
26 **Number of values**	5	5	5	

ANVOA results — Multiple comparisons

Ordinary one-way ANOVA (Multiple comparisons)								
1 Number of families	1							
2 Number of comparisons per family	2							
3 Alpha	0.05							
4								
5 **Dunnett's multiple comparisons test**	**Mean Diff.**	**95.00% CI of diff.**	**Significant?**	**Summary**	**Adjusted P Value**	**A-?**		
6 CTR vs. Rot 3h	-15.10	-30.11 to -0.08975	Yes	*	0.0487	B	Rot 3h	
7 CTR vs. Rot 6h	2.354	-12.66 to 17.36	No	ns	0.8952	C	Rot 6h	
8								
9 **Test details**	**Mean 1**	**Mean 2**	**Mean Diff.**	**SE of diff.**	**n1**	**n2**	**q**	**DF**
10 CTR vs. Rot 3h	100.0	115.1	-15.10	5.998	5	5	2.517	12
11 CTR vs. Rot 6h	100.0	97.65	2.354	5.998	5	5	0.3925	12
12								
13								

Supplementary M&M

All antibodies used in this study have been previously used by the corresponding author in Western blot experiments published in Martorana et al., CDD 2018; Goglia et al., FEBS J 2024).

List of Antibodies used

Phospho-DRP1 (Ser616) (D9A1) Rabbit Monoclonal Antibody, Cell Signaling Technology #4494

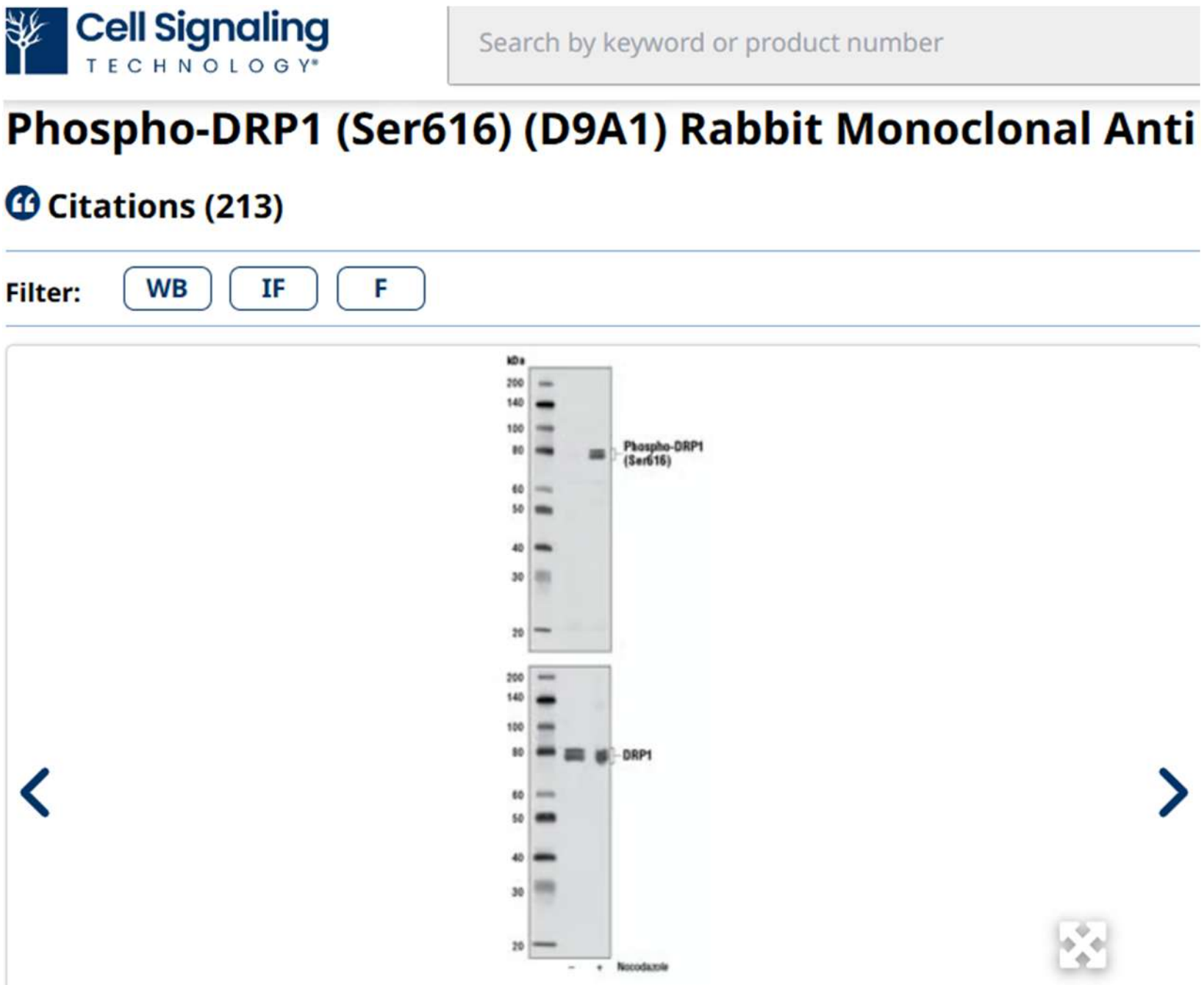

Western blot analysis of extracts from HeLa cells, untreated (-) or treated (+) with nocodazole (100 ng/ml, 17 hr), using Phospho-DRP1 (Ser616) (D9A1) Rabbit mAb (upper) and DRP1 (D6C7) Rabbit mAb #8570 (lower).

Phospho-DRP1 (Ser637) rabbit Antibody, Cell Signaling Technology #4867

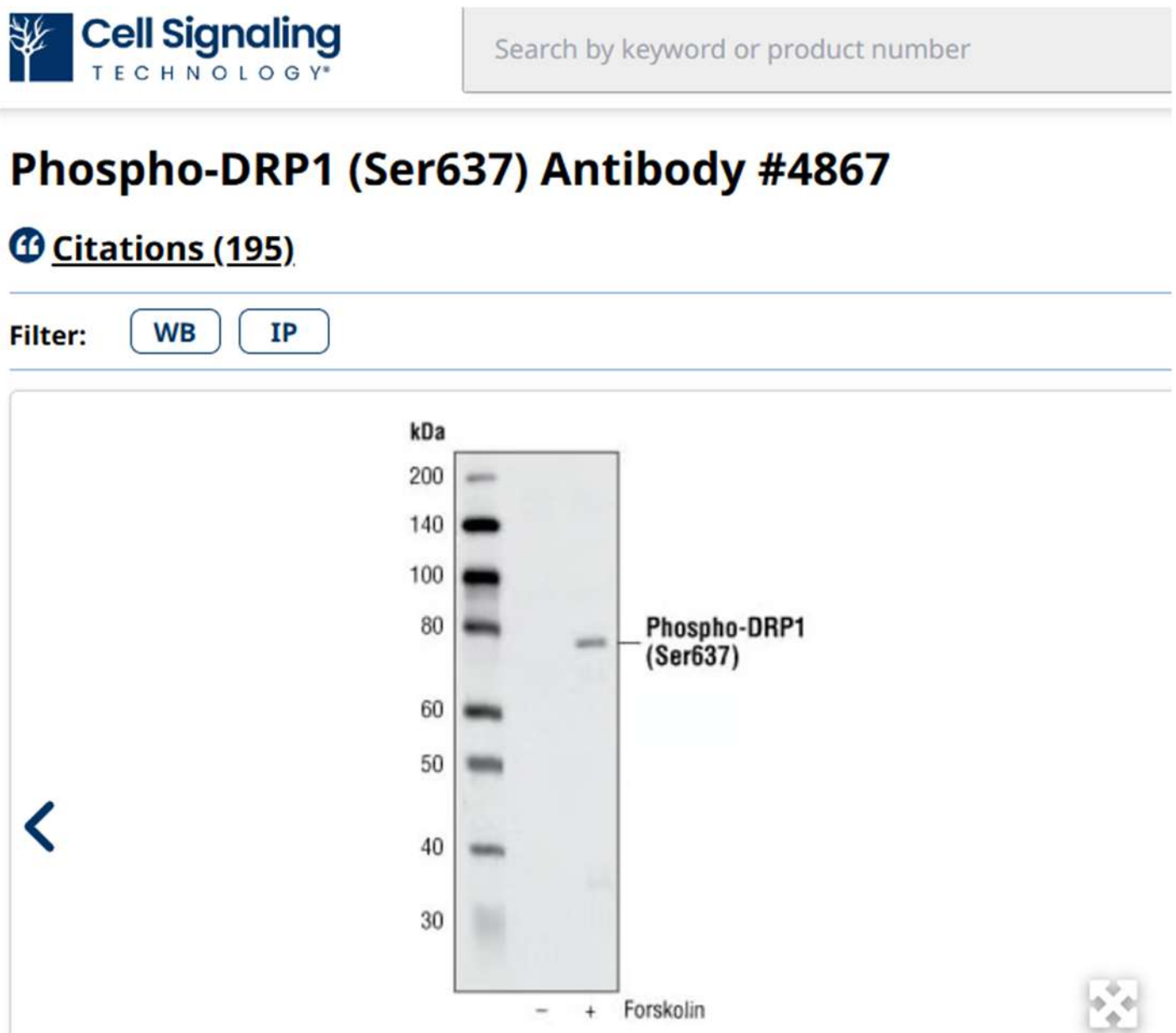

Western blot analysis of extracts from PC-12 cells, untreated or treated with 20uM Forskolin #3828 for 1 hour, using Phospho-DRP1 (Ser637) Antibody.

DRP1 (D6C7) Rabbit Monoclonal Antibody, Cell Signaling Technology #8570

Cell Signaling TECHNOLOGY

Search by keyword or product number

T 20 µL Trial Size Available R Recombinant

DRP1 (D6C7) Rabbit Monoclonal Antibody #8570

Citations (594)

Filter: WB IP IF

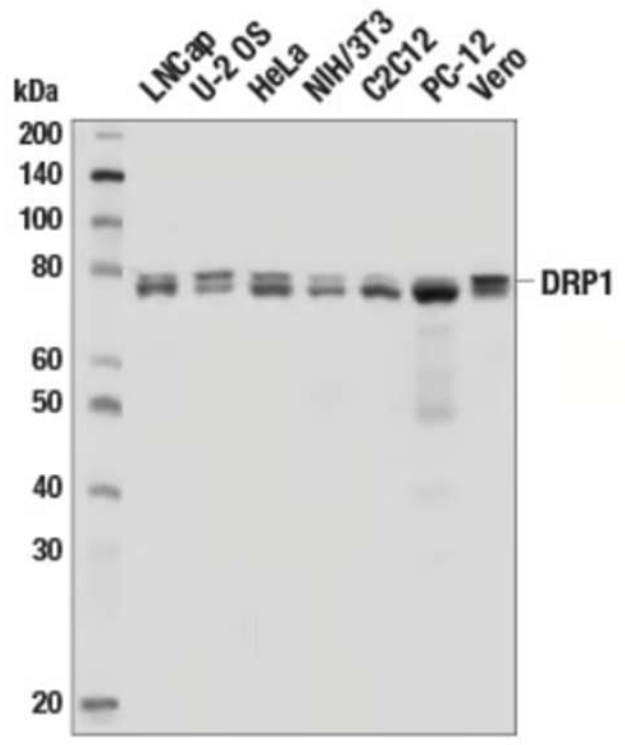

Western blot analysis of extracts from various cell lines using DRP1 (D6C7) Rabbit mAb.

Mitofusin-2 (D2D10) Rabbit Monoclonal Antibody, Cell Signaling Technology #9482

Cell Signaling TECHNOLOGY

Search by keyword or product number

Mitofusin-2 (D2D10) Rabbit Monoclonal Antibody #94

Citations (379)

Filter: WB IHC IF

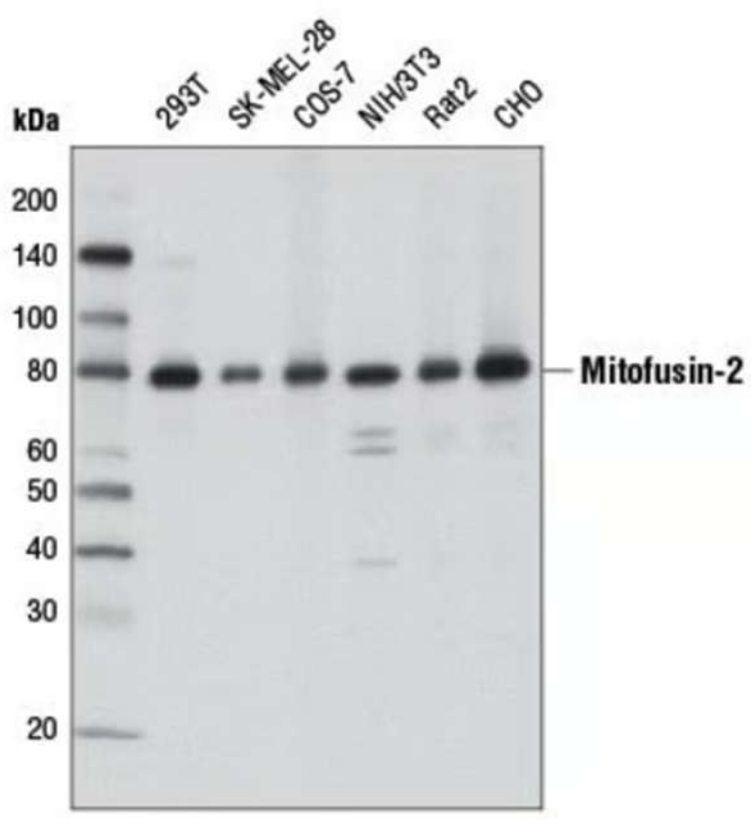

Western blot analysis of extracts from various cell lines using Mitofusin-2 (D2D10) Rabbit mAb.

(612607) BD Transduction Laboratories™ Purified Mouse Anti-OPA1

(612607) BD Transduction Laboratories™ Purified Mouse Anti-OPA1

A monoclonal mouse antibody, supplied by BD Biosciences, raised against Dynamin-like GTPase OPA1, mitochondrial (Human), cited in 166 publications, with 65 published images. Validated by knockdown. Applications used include WB, ICC, IHC, FC/FACS, and 1 other.

Go to: Supplier info | Comments | 65 images | 166 citations

General Information

**Supplier**	BD Biosciences	**Target**	Dynamin-like GTPase OPA1, mitochondrial (Human)
**Host**	Mouse	**UniProt**	O60313 - OPA1_HUMAN
**Type**	Primary	**Gene**	OPA1
**Clonality**	Monoclonal (18/OPA1)	**Modification**	Unmodified
**Conjugate**	Unconjugated		

SQSTM1/p62 Cell Signaling Technology, rabbit antibody #5114

Cell Signaling TECHNOLOGY® PRODUCTS | APPLICATIONS | RESEARCH

SQSTM1/p62 Antibody #5114

Citations (1745)

Filter: WB

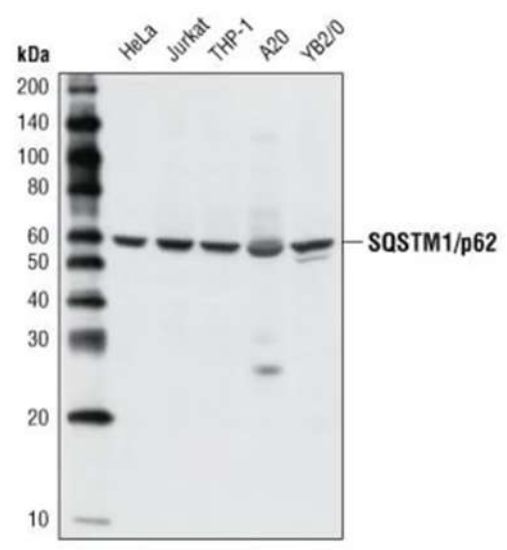

Western blot analysis of extracts from various cell lines using SQSTM1/p62 Antibody.

beta-Actin (8H10D10) Cell Signaling Technology Mouse Monoclonal Antibody #3700

Cell Signaling TECHNOLOGY® Search by keyword or product number

beta-Actin (8H10D10) Mouse Monoclonal Antibody #3700

Citations (5711)

Filter: WB | IHC | IF | F

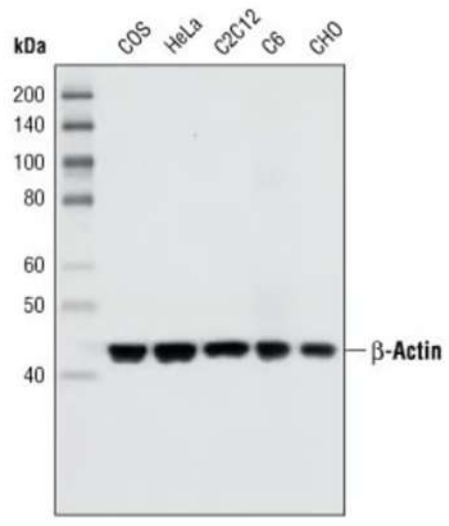

Western blot analysis of extracts from various cell types using β-Actin (8H10D10) Mouse mAb.

Western Blots in Figure 9A

Panel A1(right) shows the nitrocellulose membrane with the molecular weight markers and how the membrane was cut immediately above the band corresponding to 55 KDa. The upper membrane was probed for P(616)-Drp1, stripped and reprobed for Drp1, while the lower part of the membrane was probed for β-actin, to normalize for protein content (not shown in the main figure).

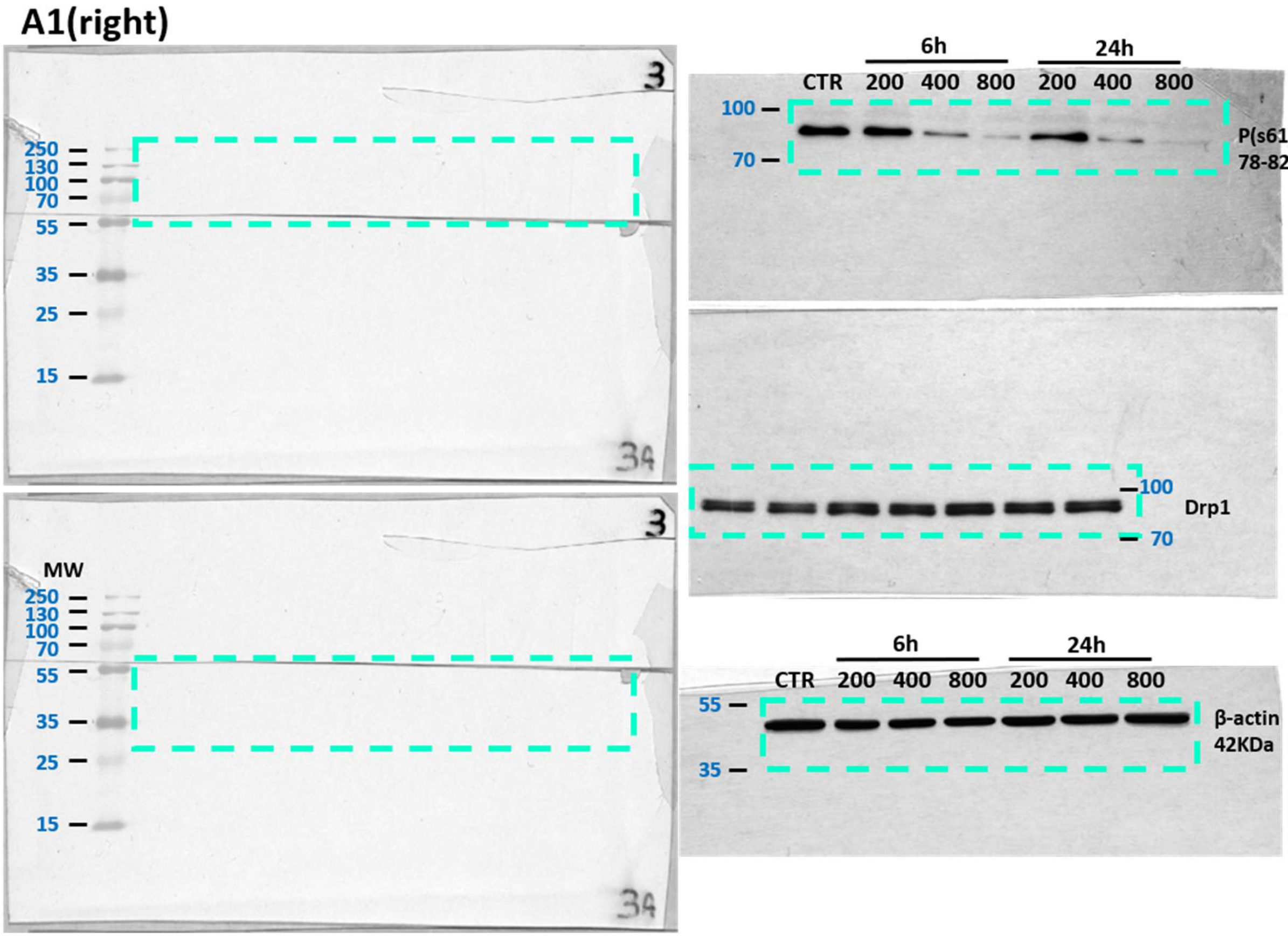

Panel A2(right) shows the nitrocellulose membrane with the molecular weight markers and how the membrane was cut immediately above the band corresponding to 55 KDa. The upper membrane (#2B) was probed for Opa1. The lower membrane (#2A) was probed for Mfn2. Please note that, although membranes are both labelled #2, they are distinctly labelled in the lower part and the markers are different. The lower parts of the membranes were probed for β-actin (β-actin for Mfn2 was used in the main figure 9A).

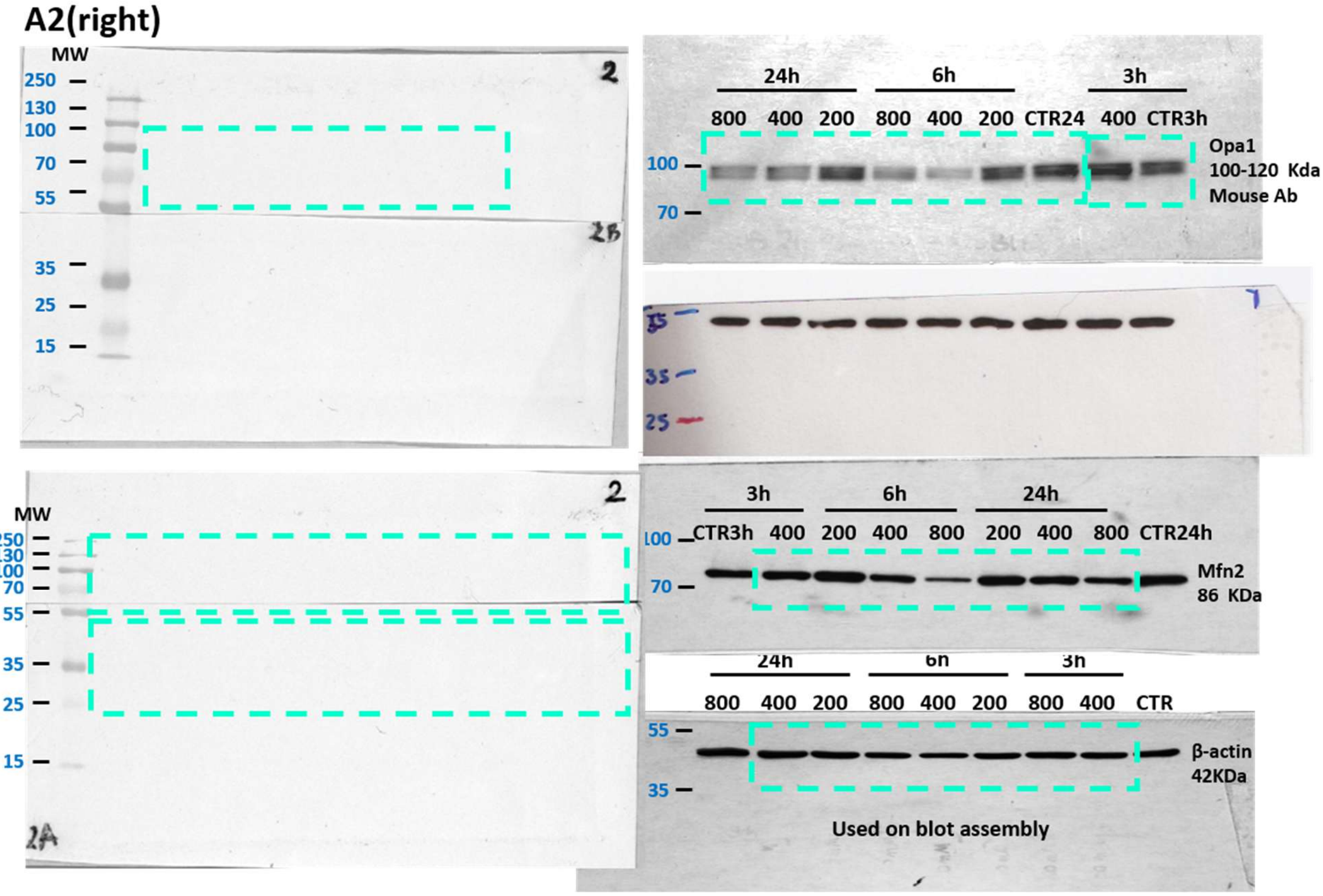

Panel A3(left) shows the two nitrocellulose membranes used for the 3h samples, showing the molecular weight markers and how both membranes were cut immediately above the band corresponding to 55 KDa.

The upper membrane 2 was probed for P(616)-Drp1, stripped and reprobed for Drp1, while the upper membrane 4 was probed for Opa1 (mouse antibody), stripped and reprobed for Mfn2 (rabbit antibody). The lower part of both membranes was probed for β-actin, to normalize for protein content. The β-actin for Opa1 and Mfn2 (lower films) has been used in main figure 9A.

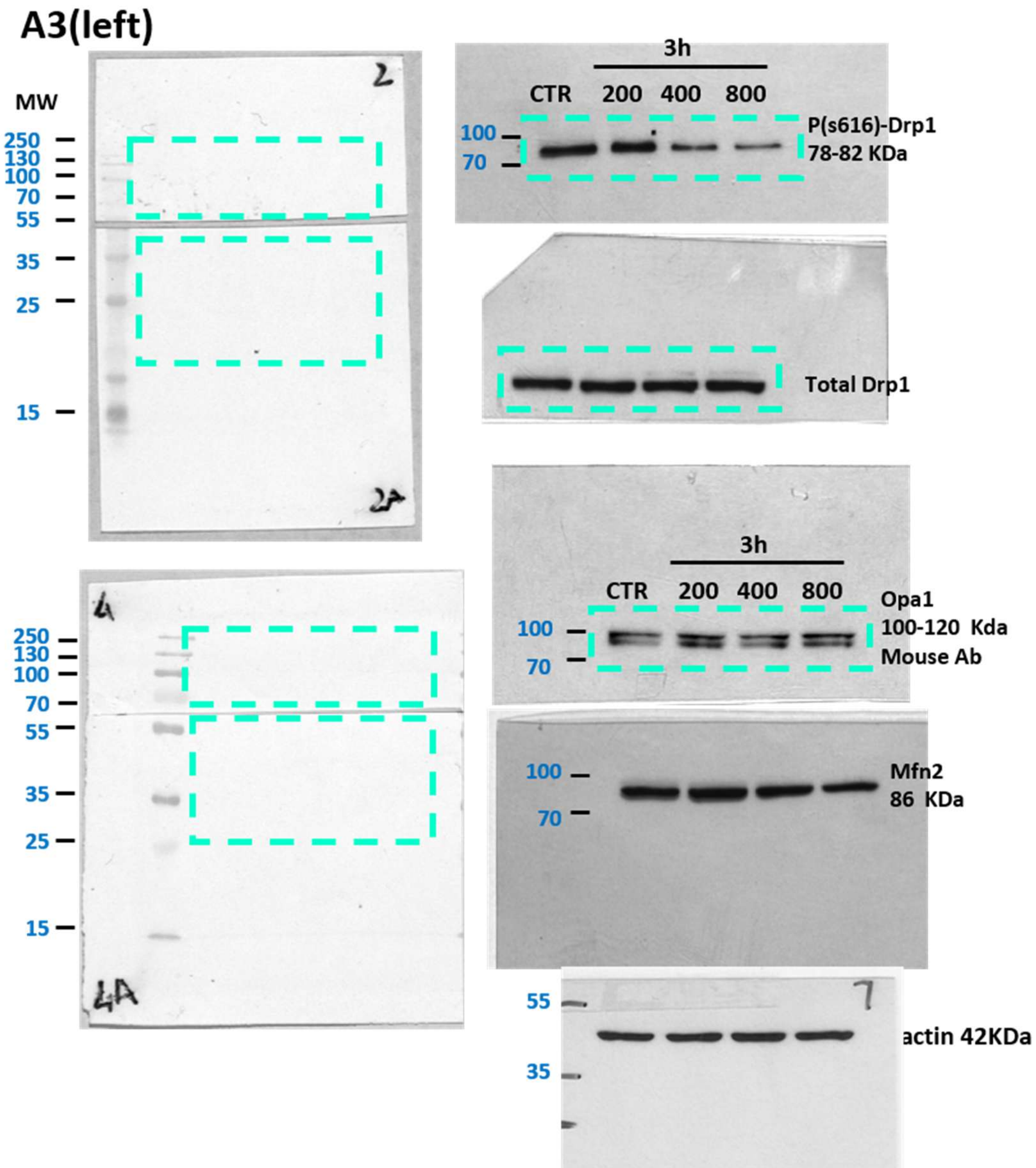

Western Blots in Figure 9D

The upper membrane (labelled 6) was cut immediately above the band corresponding to 55 KDa, probed for P(637)-Drp1, stripped and reprobed for Drp1, while the lower part of this membrane was probed for β-actin (used in the main figure). The lower membrane (labelled 1) was directly probed for p62, stripped and probed for β-actin (parallel lanes were loaded with other samples not showed in this work).

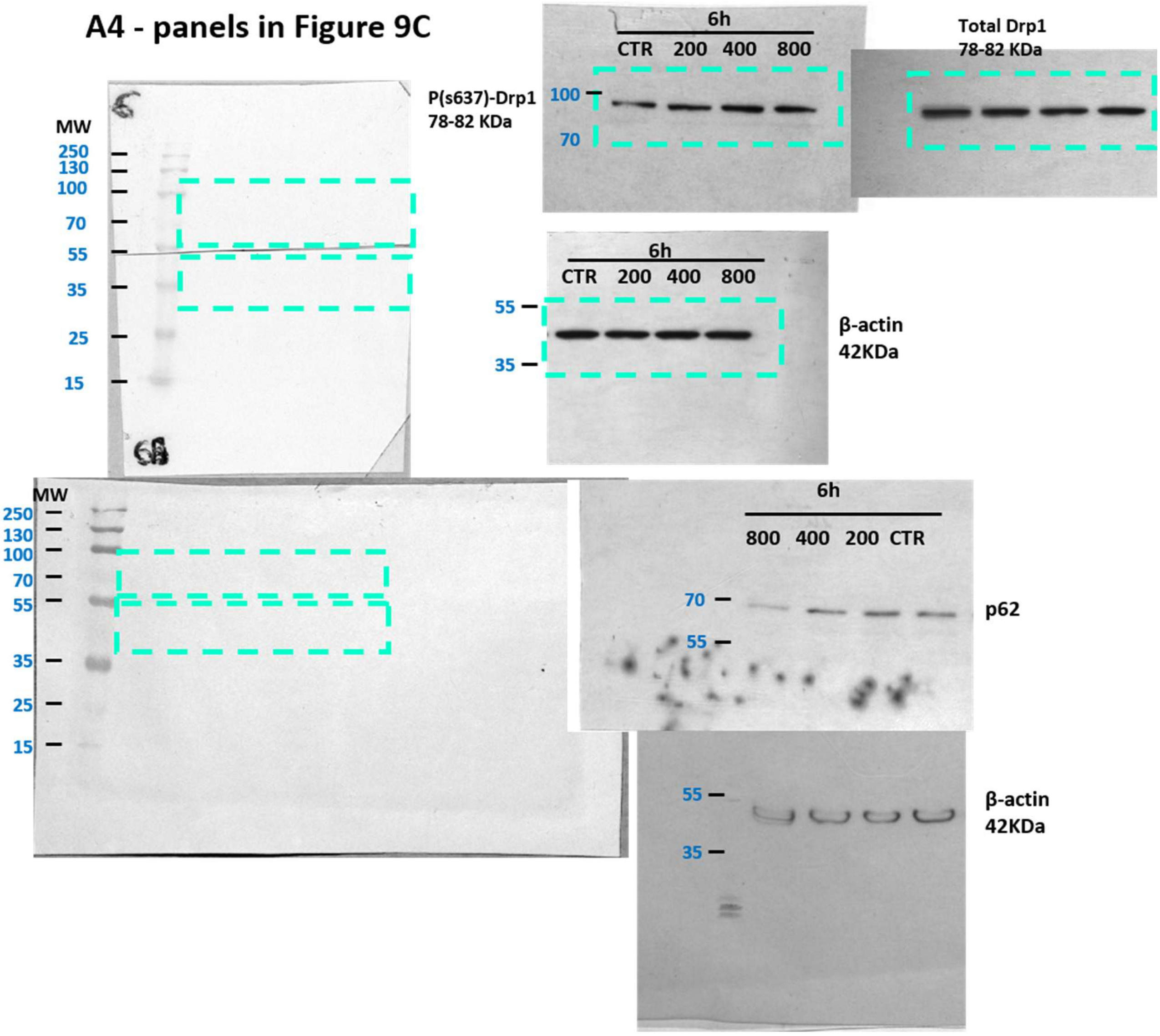

Full Blots for Supplementary Figure 3

Nitrocellulose membranes were cut immediately above the band corresponding to 55 KDa. Membrane 1 was probed for P(S616)-Drp1, stripped and reprobed for total Drp1. Membrane 3 was probed for Opa1 (mouse antibody), stripped and reprobed for Mfn2 (rabbit antibody). The lower parts of both membranes were probed for β-actin. Please note that while some markers (particularly OPA1) exhibit a dense exposure profile, they fully demonstrate a stable, unaltered expression pattern under sub-toxic treatments compared to the marked degradation cascades observed above the neurotoxic threshold (200 nM; Figure 9).

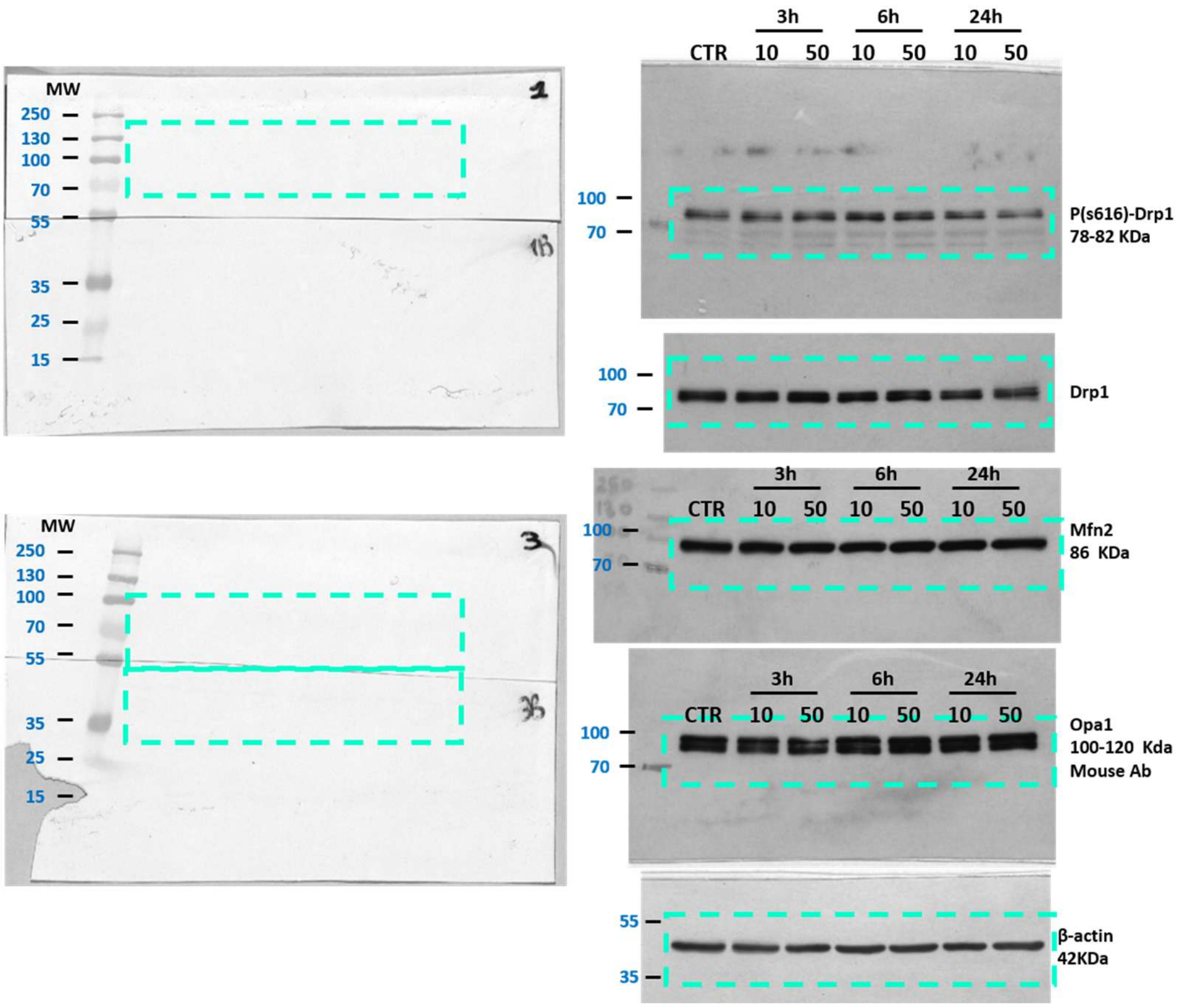

Supplement: Supplementary file 1 [file 10571_2026_1794_MOESM1_ESM.pdf]
